# A Survey of Function Analysis and Applied Dynamic Equations on Hybrid Time Scales

**DOI:** 10.3390/e23040450

**Published:** 2021-04-11

**Authors:** Chao Wang, Ravi P. Agarwal

**Affiliations:** 1 Department of Mathematics, Yunnan University, Kunming 650091, China; chaowang@ynu.edu.cn; 2 Department of Mathematics, Texas A&M University-Kingsville, Kingsville, TX 78363-8202, USA

**Keywords:** dynamic equations, time scales, general theory, 34N05, 26E70

## Abstract

As an effective tool to unify discrete and continuous analysis, time scale calculus have been widely applied to study dynamic systems in both theoretical and practical aspects. In addition to such a classical role of unification, the dynamic equations on time scales have their own unique features which the difference and differential equations do not possess and these advantages have been highlighted in describing some complicated dynamical behavior in the hybrid time process. In this review article, we conduct a survey of abstract analysis and applied dynamic equations on hybrid time scales, some recent main results and the related developments on hybrid time scales will be reported and the future research related to this research field is discussed. The results presented in this article can be extended and generalized to study both pure mathematical analysis and real applications such as mathematical physics, biological dynamical models and neural networks, etc.

## 1. Basic Knowledge on Time Scales

In 1988, S. Hilger initiated the theory of time scales in his PhD thesis [1] to unify continuous and discrete analysis. The theory is more general and versatile than the traditional theories of differential and difference equations since it is an optimal way to accurately depict the continuous-discrete hybrid processes under one framework and have been widely applied to physics, chemical technology, population dynamics, biotechnology and economics, neural networks, and social sciences. It is well-known that the dynamic equations with time scale form contains, links, and extends the classical theory of differential and difference equations. Since a time scale is an arbitrary nonempty closed subset of R, we will have a result for difference equations if T=Z and obtain a result for differential equations if T=R. This theory represents a powerful tool for applications to economics, population models, and quantum physics, among others. Not only does the new theory of the so-called “dynamic equations” unify the theories of differential equations and difference equations, but it also extends these classical cases to cases “in between,” e.g., to the so-called q-difference equations when $T=\bar{q^{N_{0}}}:=\left\{q^{t}:t\in N_{0}\;\mathrm{for}\;q>1\right\}\cup \left\{0\right\}$ or $T=\bar{q^{Z}}:=q^{Z}\cup \left\{0\right\}$ (which has important applications in quantum theory) and can be applied on different types of time scales like $T=hN,\;T=N^{2}$ and $T=T_{n}$ the space of the harmonic numbers. Therefore, dealing with problems of differential equations on time scales becomes very important and meaningful in function analysis and applied dynamic equations.

In the sequel, we will provide some necessary knowledge that will be used in this review article.

A time scale T is a closed subset of R. It follows that the jump operators σ,ϱ:T→T are defined by σ(t)=inf{s∈T:s>t} and ϱ(t)=sup{s∈T:s<t} with a stipulation that inf∅=supT (i.e., σ(t)=t if T has a maximum *t*) and sup∅=infT (i.e., ρ(t)=t if T has a minimum *t*), where *∅* denotes the empty set. If σ(t)>t, we say *t* is right scattered, while if ρ(t)<t we say *t* is left-scattered. Points that are right-scattered and left-scattered at the same time are called isolated. In addition, if t<supT and σ(t)=t, then *t* is called right-dense, and t>infT and ρ(t)=t, then *t* is called left-dense. Points that are right dense and left-dense at the same time are called dense. The mapping ν:T→[0,∞) such that ν(t)=t−ρ(t) is called the backward graininess function, the mapping μ:T→[0,∞) such that μ(t)=σ(t)−t is called the forward graininess function. Note that both σ(t) and ρ(t) are in T when t∈T, this is because T is a closed subset of R. Define

$$T^{\kappa }=\left\{\begin{array}{c} T\setminus \left\{\left(\rho (\sup(T)),\sup T\right]\cap T\right\}\;\;\mathrm{if}\;\sup T<\infty ,\\ T\;\;\;\;\;\;\;\;\;\;\;\;\;\;\mathrm{if}\;\sup T=\infty . \end{array}\right.$$

Likewise, $T_{\kappa }$ is defined as the set $T_{\kappa }=T\setminus \left\{\left[\inf T,\sigma (\inf T)\right)\cap T\right\}$ if |infT|<∞ and $T_{\kappa }=T$ if infT=−∞. If f:T→R is a function, then the function $f^{\sigma },f^{\rho }:T\to R$ is defined by $f^{\sigma }\left(t\right)=f\left(\sigma (t)\right)$ and $f^{\rho }\left(t\right)=f\left(\rho (t)\right)$ for all t∈T, respectively, i.e., $f^{\sigma }=f\circ \sigma$ and $f^{\rho }=f\circ \rho$.

Throughout the paper, for the intervals on time scales, we make the assumption that *a* and *b* are the points in T. For a≤b, we will denote the time scale interval

$${\left[a,b\right]}_{T}=\left\{t\in T:a\leq t\leq b\right\}.$$

Open intervals and half-open intervals, etc. are defined accordingly. Note that ${\left[a,b\right]}_{T}^{\kappa }={\left[a,b\right]}_{T}$ if *b* is left-dense and ${\left[a,b\right]}_{T}^{\kappa }={\left[a,b\right)}_{T}={\left[a,\rho \left(b\right)\right]}_{T}$ if *b* is left-scattered. Similarly, ${\left({\left[a,b\right]}_{T}\right)}_{\kappa }={\left[a,b\right]}_{T}$ if *a* is right-dense and ${\left({\left[a,b\right]}_{T}\right)}_{\kappa }={\left(a,b\right]}_{T}={\left[\sigma \left(a\right),b\right]}_{T}$ if *a* is right-scattered.

### 1.1. Some Basic Knowledge of Δ-Calculus

**Definition** **1** ([2,3])**.** *A function f:T→R is called regulated provided its right-sided limits exist (finite) at all right-dense points in T and its left-sided limits exist (finite) at all left-dense points in T.*

**Definition** **2** ([2,3])**.** *The function f:T→R is called rd-continuous provided that it is continuous at each right-dense point and has a left-sided limit at left dense points. The set of rd-continuous functions f:T→R will be denoted in this book by $C_{rd}\left(T\right)=C_{rd}\left(T,R\right)$. The set of functions f:T→R that are *Δ*-differentiable and whose derivative is rd-continuous is denoted by $C_{rd}^{1}\left(T\right)=C_{rd}^{1}\left(T,R\right)$.*

**Definition** **3** ([2,3])**.** *Assume f:T→R is a function and let $t\in T^{\kappa }$. Then, we define $f^{\Delta }\left(t\right)$ to be the number (provided it exists) with the property that given any ε>0, there exists a neighborhood U of t (i.e., U=(t−δ,t+δ)∩T for some δ>0) such that*

$$\begin{array}{c} |f\left(\sigma \left(t\right)\right)-f\left(s\right)-f^{\Delta }\left(t\right)\left[\sigma \left(t\right)-s\right]|\leq \varepsilon |\sigma \left(t\right)-s| \end{array}$$

*for all s∈U, we call $f^{\Delta }\left(t\right)$ the delta (or Hilger) derivative of f at t. A function F:T→R is called an antiderivative of f:T→R provided*

$$F^{\Delta }\left(t\right)=f\left(t\right)\;\;holds\;for\;all\;t\in T^{\kappa },$$

*and we define the Cauchy delta integral of f by*

$$\int_{a}^{t}f\left(s\right)\Delta s=F\left(t\right)-F\left(a\right)\;\;\;for\;all\;t,a\in T.$$

**Theorem** **1** ([2,3])**.** *Assume f,g:T→R are differentiable at $t\in T^{\kappa }$. Then:*

(*i*) *The sum f+g:T→R are differentiable at t with*
  $${\left(f+g\right)}^{\Delta }\left(t\right)=f^{\Delta }\left(t\right)+g^{\Delta }\left(t\right).$$
(*ii*) *For any constant α,αf:T→R is differentiable at t with*
  $${\left(\alpha f\right)}^{\Delta }=\alpha f^{\Delta }\left(t\right).$$
(*iii*) *The product fg:T→R is differentiable at t with*
  $${\left(fg\right)}^{\Delta }\left(t\right)=f^{\Delta }\left(t\right)g\left(t\right)+f\left(\sigma (t)\right)g^{\Delta }\left(t\right)=f\left(t\right)g^{\Delta }\left(t\right)+f^{\Delta }\left(t\right)g\left(\sigma (t)\right).$$
(*iv*) *If $f\left(t\right)f\left(\sigma (t)\right)\neq 0$, then $\frac{1}{f}$ is differentiable at t with*
  $${\left(\frac{1}{f}\right)}^{\Delta }\left(t\right)=-\frac{f^{\Delta }\left(t\right)}{f\left(t\right)f\left(\sigma (t)\right)}.$$
(*v*) *If $g\left(t\right)g\left(\sigma (t)\right)\neq 0$, then $\frac{f}{g}$ is differentiable at t and*
  $${\left(\frac{f}{g}\right)}^{\Delta }\left(t\right)=\frac{f^{\Delta }\left(t\right)g\left(t\right)-f\left(t\right)g^{\Delta }\left(t\right)}{g\left(t\right)g\left(\sigma (t)\right)}.$$

**Theorem** **2** ([2,3])**.** *If a,b,c∈T,α,β∈R, and $f,g\in C_{rd}$, then*

(*i*) *$\int_{a}^{b}\left[\alpha f(t)+\beta g(t)\right]\Delta t=\alpha \int_{a}^{b}f\left(t\right)\Delta t+\beta \int_{a}^{b}g\left(t\right)\Delta t$;*
(*ii*) *$\int_{a}^{b}f\left(t\right)\Delta t=-\int_{b}^{a}f\left(t\right)\Delta t$;*
(*iii*) *$\int_{a}^{c}f\left(t\right)\Delta t=\int_{a}^{b}f\left(t\right)\Delta t+\int_{b}^{c}f\left(t\right)\Delta t$;*
(*iv*) *$|\int_{a}^{b}f\left(t\right)\Delta t|\leq \int_{a}^{b}\left|f\left(t\right)\right|\Delta t$.*

**Definition** **4** ([2,3])**.** *For h>0, we define the Hilger complex numbers, the Hilger real axis, the Hilger alternating axis, and the Hilger imaginary circle as*

$$C_{h}:=\left\{z\in C:z\neq -\frac{1}{h}\right\},$$

$$R_{h}:=\left\{z\in C_{h}:z\in R\;and\;z>-\frac{1}{h}\right\},$$

$$A_{h}:=\left\{z\in C_{h}:z\in R\;and\;z<-\frac{1}{h}\right\},$$

$$I_{h}:=\left\{z\in C_{h}:|z+\frac{1}{h}|=\frac{1}{h}\right\},$$

*respectively. For h=0, let $C_{0}:=C,R_{0}:=R,I_{0}=iR$, and $A_{0}:=\emptyset$.*

**Definition** **5** ([2,3])**.** *Let h>0 and $z\in C_{h}$. We define the Hilger real part of z by*

$$Re_{h}\left(z\right):=\frac{|zh+1|-1}{h}$$

*and the Hilger imaginary part of z by*

$$Im_{h}\left(z\right):=\frac{Arg(zh+1)}{h},$$

*where Arg(z) denotes the principle argument of z (i.e., −π<Arg(z)≤π). Note that $Re_{h}\left(z\right)$ and $Im_{h}\left(z\right)$ satisfy*

$$-\frac{1}{h}<Re_{h}\left(z\right)<\infty \;\;\mathrm{and}\;\;-\frac{\pi }{h}<Im_{h}\left(z\right)\leq \frac{\pi }{h},$$

*respectively. In particular, $Re_{h}\left(z\right)\in R_{h}$.*

**Definition** **6** ([2,3])**.** *Let $-\frac{\pi }{h}<\omega \leq \frac{\pi }{h}$. We define the Hilger purely imaginary number $\overset{˚}{\iota }\omega$ by*

$$\overset{˚}{\iota }\omega =\frac{e^{i\omega h}-1}{h}.$$

*For $z\in C_{h}$, $\overset{˚}{\iota }Im_{h}\left(z\right)\in I_{h}$.*

**Theorem** **3** ([2,3])**.** *If the “circle plus” addition *⊕* is defined by z⊕ω:=z+ω+zωh, then $\left(C_{h},⊕\right)$ is an Abelian group. For $z\in C_{h}$, we have $z=Re_{h}\left(z\right)⊕\overset{˚}{\iota }Im_{h}\left(z\right)$.*

**Definition** **7** ([2,3])**.** *The “circle minus” substraction *⊖* on $C_{h}$ is defined by z⊖ω:=z⊕(⊖ω), where $⊖\omega :=\frac{-\omega }{1+\omega h}$.*

For h>0, let $Z_{h}$ be the strip

$$Z_{h}:=\left\{z\in C:-\frac{\pi }{h}<Im\left(z\right)\leq \frac{\pi }{h}\right\},$$

and for h=0, let $Z_{0}:=C$.

**Definition** **8** ([2,3])**.** *For h>0, the cylinder transformation $\xi_{h}:C_{h}\to Z_{h}$ by*

$$\xi_{h}\left(z\right)=\frac{1}{h}Log\left(1+zh\right),$$

*where Log is the principal logarithm function. For h=0, we define $\xi_{0}\left(z\right)=z$ for all z∈C.*

We define addition on $Z_{h}$ by

(1) $$z+\omega :=z+\omega \;\;\left(mod\frac{2\pi i}{h}\right)\;\;\mathrm{for}\;z,\omega \in Z_{h}.$$

**Theorem** **4** ([2,3])**.** *The inverse transformation of the cylinder transformation $\xi_{h}$ when h>0 is given by*

$$\xi_{h}^{-1}\left(z\right)=\frac{1}{h}\left(e^{zh}-1\right)$$

*for $z\in Z_{h}$. For h=0, $\xi_{0}^{-1}\left(z\right)=z$.*

**Theorem** **5** ([2,3])**.** *The cylinder transformation $\xi_{h}$ is a group homomorphism from $\left(C_{h},⊕\right)$ onto $\left(Z_{h},+\right)$, where the addition + on $Z_{h}$ is defined by* (1).

**Definition** **9** ([2,3])**.** *A function p:T→R is called μ- regressive provided 1+μ(t)p(t)≠0 for all $t\in T^{\kappa }$. The set of all regressive and rd-continuous functions p:T→R will be denoted by R=R(T)=R(T,R). We define the set $R^{+}=R^{+}\left(T,R\right)=\left\{p\in R:1+\mu \left(t\right)p\left(t\right)>0,\forall \;t\in T\right\}$. The set of all regressive functions on a time scale T forms an Abelian group under the addition *⊕* defined by p⊕q:=p+q+μpq.*

**Definition** **10** ([2,3])**.** *If r is a μ-regressive function, then the generalized exponential function $e_{r}$ is defined by*

$$e_{r}\left(t,s\right)=\exp\left\{\int_{s}^{t}\xi_{\mu (\tau )}\left(r\left(\tau \right)\right)\Delta \tau \right\}$$

*for all s,t∈T, where the μ-cylinder transformation is as in*

$$\xi_{h}\left(z\right):=\frac{1}{h}\mathrm{Log}\left(1+zh\right).$$

**Theorem** **6** ([2,3])**.** *Assume that p,q:T→R are two μ-regressive functions. Then,*

(i) $e_{0}\left(t,s\right)\equiv 1$ and $e_{p}\left(t,t\right)\equiv 1$;
(ii) $e_{p}\left(\sigma \left(t\right),s\right)=\left(1+\mu \left(t\right)p\left(t\right)\right)e_{p}\left(t,s\right)$;
(iii) $e_{p}\left(t,s\right)=\frac{1}{e_{p}\left(s,t\right)}=e_{⊖p}\left(s,t\right)$;
(iv) $e_{p}\left(t,s\right)e_{p}\left(s,r\right)=e_{p}\left(t,r\right)$;
(v) ${\left(e_{⊖p}\left(t,s\right)\right)}^{\Delta }=\left(⊖p\right)\left(t\right)e_{⊖p}\left(t,s\right)$.

### 1.2. Some Basic Knowledge of ∇-Calculus

In this subsection, we will introduce some basic knowledge of ∇-Calculus.

**Definition** **11** ([2,3])**.** *The function f:T→R is called ld-continuous provided that it is continuous at each left-dense point and has a right-sided limit at right-dense points. The set of ld-continuous functions f:T→R is denoted by $C_{ld}\left(T\right)=C_{ld}\left(T,R\right)$. The set of functions f:T→R that are *∇*-differentiable and whose derivative is ld-continuous is denoted by $C_{ld}^{1}\left(T\right)=C_{ld}^{1}\left(T,R\right)$.*

**Definition** **12** ([2,3])**.** *The function f:T→R is called ld-continuous provided that it is continuous at each left-dense point and has a right-sided limit at each point, write $f\in C_{ld}\left(T\right)=C_{ld}\left(T,R\right)$. Let $t\in T_{\kappa }$. Then, we define $f^{\nabla }\left(t\right)$ to be the number (provided it exists) with the property that given any ε>0, there exists a neighborhood U of t (i.e., U=(t−δ,t+δ)∩T for some δ>0) such that*

$$\begin{array}{c} |f\left(\rho \left(t\right)\right)-f\left(s\right)-f^{\nabla }\left(t\right)\left[\rho \left(t\right)-s\right]|\leq \varepsilon |\rho \left(t\right)-s| \end{array}$$

*for all s∈U, we call $f^{\nabla }\left(t\right)$ the nabla derivative of f at t. A function F:T→R is called an antiderivative of f:T→R provided*

$$F^{\nabla }\left(t\right)=f\left(t\right)\;\;holds\;for\;all\;t\in T_{\kappa },$$

*and we define the Cauchy nabla integral of f by*

$$\int_{a}^{t}f\left(s\right)\nabla s=F\left(t\right)-F\left(a\right)\;\;\;for\;all\;t,a\in T.$$

**Definition** **13** ([2,3])**.** *A function p:T→R is called ν- regressive provided 1−ν(t)p(t)≠0 for all $t\in T_{k}$. The set of all regressive and ld-continuous functions p:T→R will be denoted by $R_{\nu }=R_{\nu }\left(T\right)=R_{\nu }\left(T,R\right)$. We define the set $R_{\nu }^{+}=R_{\nu }^{+}\left(T,R\right)=\left\{p\in R_{\nu }:1-\nu \left(t\right)p\left(t\right)>0,\forall \;t\in T\right\}$. We define circle plus addition by $p{⊕}_{\nu }q=p\left(t\right)+q\left(t\right)-\nu \left(t\right)p\left(t\right)q\left(t\right)$ for all $t\in T_{\kappa }$.*

**Theorem** **7** ([2,3])**.** *The set $\left(R_{\nu },{⊕}_{\nu }\right)$ is an Abelian group.*

**Definition** **14** ([2,3])**.** *For $p\in R_{\nu }$, define circle minus by*

$${⊖}_{\nu }p=-\frac{p}{1-\nu p}.$$

**Definition** **15** ([2,3])**.** *If r is a regressive function, then the generalized exponential function ${\hat{e}}_{r}$ is defined by*

$${\hat{e}}_{r}\left(t,s\right)=\exp\left\{\int_{s}^{t}{\hat{\xi }}_{\nu (\tau )}\left(r\left(\tau \right)\right)\nabla \tau \right\}$$

*for all s,t∈T, where the ν-cylinder transformation is as in*

$${\hat{\xi }}_{h}\left(z\right):=-\frac{1}{h}\mathrm{Log}\left(1-zh\right).$$

**Lemma** **1** ([2,3])**.** *Assume that p,q:T→R are two ν-regressive functions. Then,*

(i) ${\hat{e}}_{0}\left(t,s\right)\equiv 1$ and ${\hat{e}}_{p}\left(t,t\right)\equiv 1$;
(ii) ${\hat{e}}_{p}\left(\varrho \left(t\right),s\right)=\left(1-\nu \left(t\right)p\left(t\right)\right){\hat{e}}_{p}\left(t,s\right)$;
(iii) ${\hat{e}}_{p}\left(t,s\right)=\frac{1}{{\hat{e}}_{p}\left(s,t\right)}=e_{{⊖}_{\nu }p}\left(s,t\right)$;
(iv) ${\hat{e}}_{p}\left(t,s\right){\hat{e}}_{p}\left(s,r\right)={\hat{e}}_{p}\left(t,r\right)$;
(v) ${\left({\hat{e}}_{{⊖}_{\nu }p}\left(t,s\right)\right)}^{\nabla }=\left({⊖}_{\nu }p\right)\left(t\right){\hat{e}}_{{⊖}_{\nu }p}\left(t,s\right)$.

## 2. Almost Periodic and Almost Automorphic Theory on Time Scales

Almost periodic phenomena are very common and almost periodic theory plays a significant role in natural science. Almost periodicity is an important feature of dynamical systems that will inaccurately retrace their paths through phase space, for example, for a planetary system, all the planets in orbits move in commensurable periods (i.e., a period vector is not proportional to a vector of integers). In mathematics, within any desired level of precision of periodicity, an almost periodic function is a real function with a suitably long, well-distributed “almost-periods”. The concept was first studied by H. Bohr and later generalized by V. Stepanov, H. Weyl and A.S. Besicovitch, and John von Neumann (see [4,5,6]), etc.

Compared with periodic phenomenon, almost periodic phenomenon can describe many regular changes in nature more accurately. Almost automorphic function, as a generalization of almost periodic function, has a wider range of applications. This notion was proposed by W.A. Veech (see [7,8]) and was found in the study of differential geometry related to physics, then more and more attention has been paid to the research on the generalization of corresponding concepts and their series (see [9,10]).

In this section, we will demonstrate some main results and recent developments of almost periodic and almost automorphic theory on translation time scales and extend the topic to more complicated hybrid time cases under the matched spaces of time scales.

### 2.1. Almost Periodic and Almost Automorphic Theory on Translation Time Scales

The theory of almost periodic and almost automorphic functions have wide applications in dynamic equations (see [9]). Through using the time scale theory initiated by Hilger (see [1]), many classical results of almost periodic and almost automorphic functions were extended to different time scales. The translation doublication of two time scales is the basic requirement of introducing the notions of almost periodic and almost automorphic functions. In 2016, Wang and Agarwal et al. (see [11,12,13]) proposed some equivalent concepts of periodic time scales as follows:

**Definition** **16** ([12,13])**.** *A time scale T is called a periodic time scale (or a translation invariant time scale) if $\Pi :=\left\{\tau \in R:T\cap T^{\tau }=T\right\}\notin \left\{\{0\},\emptyset \right\},$ where $T^{\tau }=\left\{t+\tau :t\in T\right\}$.*

We can obtain that, if we choose nonzero real number τ∈Π, then $T=T^{\tau }$ if and only if T is invariant under translations.

**Definition** **17** ([12,13])**.** *A time scale T is called a periodic time scale (or a translation invariant time scale) if $\Pi :=\left\{\tau \in R:T^{\tau }\cup T^{-\tau }\subset T\right\}\notin \left\{\{0\},\emptyset \right\}.$*

**Remark** **1.** *According to Definitions 16 and 17, the translation invariance of a time scale implies that the time scale T coincides with the obtained time scale $T^{\tau }$ through a translation number τ∈R.*

**Example** **1.** *The following time scales are invariant:*

(*i*) *T=hZ, where h>0, has period P=h.*
(*ii*) *$T=\{t=k-q^{m}:\;k\in Z,\;m\in N_{0}\}$, where 0<q<1, has period P=1.*
(*iii*) *T=R has an arbitrary period P∈R∖{0}.*
(*iv*) *${⋃}_{i=-\infty }^{\infty }\left[(2i-1)h,2ih\right]$, h>0, has period P=2h.*

Based on Definitions 16 and 17, some corrected concepts of almost periodic functions were proposed (see [11,14]). In [15], some sufficient conditions were obtained for the existence and exponential stability of piecewise mean-square almost periodic solutions of the impulsive stochastic Nicholson’s blowflies model on translation time scales. In [16,17,18,19,20], the authors firstly introduced the concept of piecewise almost periodic and almost automorphic functions on time scales with periodicity and applied them to analyze the almost periodic solutions to neural networks and biological dynamic models.

**Definition** **18** ([16,18])**.** *We say $\varphi :T\to R^{n}$ is piecewise rd-continuous with respect to a sequence $\{\tau_{i}\}\subset T$ which satisfies $\tau_{i}<\tau_{i+1},\;i\in Z$, if φ(t) is continuous on ${\left[\tau_{i},\tau_{i+1}\right)}_{T}$ and rd-continuous on $T\setminus \{\tau_{i}\}$. Furthermore, ${\left[\tau_{i},\tau_{i+1}\right)}_{T},i\in Z,$ are called intervals of continuity of the function φ(t).*

**Definition** **19** ([16,18])**.** *For any ε>0, let $\Gamma_{\varepsilon }\subset \Pi$ be a set of real numbers and $\{\tau_{i}\}\subset T$. We say $\{\tau_{i}^{j}\},\;i,j\in Z$ is equipotentially almost periodic on a periodic time scale T if for $r\in \Gamma_{\varepsilon }\subset \Pi$, there exists at least one integer k such that*

$$|\tau_{i}^{k}-r|<\varepsilon ,\;\;for\;all\;i\in Z.$$

In the following, we will give the definition of piecewise rd-continuous almost periodic functions with respect to the sequence ${\left\{\tau_{i},\right\}}_{i\in Z}$ on a periodic time scale T.

**Definition** **20** ([16,18])**.** *Let T be a periodic time scale and assume that $\{\tau_{i}\}\subset T$ satisfying the derived sequence $\{\tau_{i}^{j}\},\;i,j\in Z$, is equipotentially almost periodic. A function $\varphi \in PC_{rd}\left(T,R^{n}\right)$ is said to be piecewise rd-continuous almost periodic (short for rd-piecewise almost periodic) if:*

(*i*) *for any ε>0, there is a positive number δ=δ(ε) such that if the points $t^{{}^{\prime }}$ and $t^{{}^{\prime \prime }}$ belong to the same interval of continuity and $|t^{{}^{\prime }}-t^{{}^{\prime \prime }}|<\delta$, then $∥\varphi \left(t^{{}^{\prime }}\right)-\varphi \left(t^{{}^{\prime \prime }}\right)∥<\varepsilon ;$*
(*ii*) *for any ε>0, there is relative dense set $\Gamma_{\varepsilon }\subset \Pi$ of ε-almost periods such that if $\tau \in \Gamma_{\varepsilon }$, then ∥φ(t+τ)−φ(t)∥<ε for all t∈T, which satisfies the condition $|t-\tau_{i}|>\varepsilon ,\;i\in Z$.*

Based on Definitions 18–20, some basic properties of piecewise almost periodic functions were obtained.

**Theorem** **8** ([16,18])**.** *If $\varphi \in PC_{rd}\left(T,R^{n}\right)$ is rd-piecewise almost periodic, then, for any ε>0, there exists a relative dense set of intervals of a fixed length $\gamma_{\varepsilon }\in \Pi$, which consist of ε-almost periods of the function φ(t).*

**Theorem** **9** ([16,18])**.** *Let $\varphi \in PC_{rd}\left(T,R^{n}\right)$ be an rd-piecewise almost periodic function with values in the set $E\subset R^{n}$. If F(y) is an uniformly continuous function defined on the set E, then the function $F\left(\varphi (t)\right)$ is rd-piecewise almost periodic in t.*

**Theorem** **10** ([16,18])**.** *For any two rd-piecewise almost periodic functions with respect to the same sequence $\{\tau_{i}\}\subset T$, for any ε>0, there exists a relative dense set of their common ε-almost periods.*

In fact, the above Definitions 18–20 can be generalized to Banach spaces and some basic theorems can be established in Banach space.

Now, introduce the set

$$B=\left\{\left\{t_{k}\right\}:t_{k}\in T,\;t_{k}<t_{k+1},\;k\in Z,\;\lim_{k\to \pm \infty }t_{k}=\pm \infty \right\},$$

which denotes all unbounded increasing sequences of real numbers.

Let X be a Banach space, Ω be an open set in X or Ω=X, and *S* denotes an arbitrary compact subset of Ω.

**Definition** **21** ([16,18])**.** *The functions $f,g\in PC_{rd}\left(T\times \Omega ,X\right)$ are said to be ε-equivalent uniformly for x∈Ω or f,g possess uniform ε-equivalence for x∈Ω, and denote $f\overset{\varepsilon }{∼}g$, if for all ε>0 and for each compact subset S of *Ω*, the following conditions hold:*

(*i*) *The points of possible discontinuity of these functions can be enumerated $t_{k}^{f},\;t_{k}^{g}$, admitting a finite multiplicity by the order in T, so that $|t_{k}^{f}-t_{k}^{g}|<\varepsilon$.*
(*ii*) *There exist strictly increasing sequences of numbers $\left\{t_{k}^{{}^{\prime }}\right\},\;\left\{t_{k}^{{}^{\prime \prime }}\right\}$, $t_{k}^{{}^{\prime }}<t_{k+1}^{{}^{\prime }},\;t_{k}^{{}^{\prime \prime }}<t_{k+1}^{{}^{\prime \prime }},\;k\in Z$, for which we have*
  $$\underset{t\in {\left(t_{k}^{{}^{\prime }},t_{k+1}^{{}^{\prime }}\right)}_{T},t^{{}^{\prime }}\in {\left(t_{k}^{{}^{\prime \prime }},t_{k+1}^{{}^{\prime \prime }}\right)}_{T}}{\sup}∥f\left(t,x\right)-g\left(t^{{}^{\prime }},x\right)∥<\varepsilon ,\;|t_{k}^{{}^{\prime }}-t_{k}^{{}^{\prime \prime }}|<\varepsilon ,\;\forall x\in S,\;k\in Z.$$

**Theorem** **11** ([16,18])**.** *Let $\varphi \in PC_{rd}\left(T\times \Omega ,X\right)$ be rd-piecewise almost periodic in t uniformly for x∈Ω. Then, it is uniformly rd-continuous on T∖B and bounded on T×S.*

Let T,P∈B and let s(T∪P):B→B be a map such that the set s(T∪P) forms a strictly increasing sequence. For D⊂R and 0<h∈Π, we introduce the notations $\theta_{h}\left(D\right)=\left\{t+h:t\in D\right\},\;F_{h}\left(D\right)=D\cap \left\{\theta_{h}\left(D\right)\right\}$. Denote by $\phi =\left(\varphi (t),T\right)$ the element from the space $PC_{rd}\left(T\times \Omega ,X\right)\times B$ and, for every sequence of real numbers $\{s_{n}\},\;n=1,2,\ldots$ with $\theta_{s_{n}}\phi =\left(\varphi \left(t+s_{n},x\right),T+s_{n}\right)$, we shall consider the sets $\left\{\left(\varphi \left(t+s_{n},x\right),T+s_{n}\right)\right\}\subset PC_{rd}\times B$, where

$$T+s_{n}:=T^{s_{n}}=\left\{t_{k}+s_{n}:k\in Z,\;n=1,2,\ldots \right\}.$$

For convenience, we introduce the translation operator *S*, and let us denote by $S_{\alpha +\beta }\phi$ and $S_{\alpha }S_{\beta }\phi$ the limits $\lim_{n\to \infty }\theta_{\alpha_{n}+\beta_{n}}\left(\phi \right)$ and $\lim_{n\to \infty }\theta_{\alpha_{n}}\left(\lim_{m\to \infty }\theta_{\beta_{m}}\phi \right)$, respectively, and are written only when the limits exist.

**Theorem** **12** ([16,18])**.** *The function $\varphi \in PC_{rd}\left(T\times \Omega ,X\right)$ is rd-piecewise almost periodic in t uniformly for x∈Ω with respect to a sequence T∈B if and only if from every pair of sequence $\alpha^{{}^{\prime }},\beta^{{}^{\prime }}$, one can extract common subsequences $\alpha \subset \alpha^{{}^{\prime }},\;\beta \subset \beta^{{}^{\prime }}$ such that*

$$S_{\alpha +\beta }\phi =S_{\alpha }S_{\beta }\phi$$

*exists pointwise, where $\phi =\left(\varphi (t,x),T\right)$.*

We established the following piecewise almost periodic solution of the dynamic equations on hybrid time scales.

First, we shall consider the linear dynamic equations as follows:

(2) $$\left\{\begin{array}{c} x^{\Delta }=A\left(t\right)x,\;\;\;t\neq t_{k},\\ \tilde{\Delta }x\left(t_{k}\right)=B_{k}x\left(t_{k}\right),\;\;t=t_{k},\;k\in Z, \end{array}\right.$$

where $t\in T,\;\left\{t_{k}\right\}\in B,\;A\in PC_{rd}\left(T,R^{n\times n}\right),\;B_{k}\in R^{n\times n},\;k\in Z$.

By $x\left(t\right)=x\left(t;t_{0},x_{0}\right)$, we denote the solution of (2) with initial condition by $x\left(t_{0}^{+}\right)=x_{0},\;x_{0}\in R^{n}$. Assume the following conditions hold:

(*H*_1_) The matrix-valued function $A\in PC_{rd}\left(T,R^{n\times n}\right)$ is almost periodic.
(*H*_2_) $\{B_{k}\},\;k\in Z$ is an almost periodic sequence.
(*H*_3_) $\det(E+B_{k})\neq 0,\;k\in Z$, where *E* is the identity matrix.
(*H*_4_) The set of sequence $\left\{t_{k}^{j}\right\},\;t_{k}^{j}=t_{k+j}-t_{k},\;k\in Z,\;j\in Z$ is equipotentially almost periodic and $\inf_{k}t_{k}^{1}=\theta >0$.

Now, consider the following system:

(3) $$\left\{\begin{array}{c} x^{\Delta }=A\left(t\right)x+f\left(t\right),\;\;\;t\neq t_{k},\\ \tilde{\Delta }x\left(t_{k}\right)=B_{k}x\left(t_{k}\right)+I_{k},\;\;t=t_{k},\;k\in Z. \end{array}\right.$$

**Theorem** **13** ([16,18])**.** *If $\left(H_{1}\right)-\left(H_{4}\right)$ hold, (2) admits an exponential dichotomy on T with a projection P, then (3) admits a piecewise rd-continuous almost periodic solution as follows:*

$$\begin{array}{ccc} x(t) & = & \int_{-\infty }^{t}X\left(t\right)PX^{-1}\left(\sigma \left(s\right)\right)f\left(s\right)\Delta s-\int_{t}^{+\infty }X\left(t\right)\left(E-P\right)X^{-1}\left(\sigma \left(s\right)\right)f\left(s\right)\Delta s\\ & & +\sum_{-\infty <t_{k}<t}X\left(t\right)PX^{-1}\left(t_{k}\right)I_{k}-\sum_{t<t_{k}<+\infty }X\left(t\right)\left(E-P\right)X^{-1}\left(t_{k}\right)I_{k}, \end{array}$$

*where X(t) is a fundamental matrix solution of system (2).*

In the following part, based on the translation hybrid time scales, the definition of ld-piecewise continuous functions on time scales was introduced and some basic properties of piecewise ld-continuous weighted pseudo almost automorphic functions were established.

**Definition** **22** ([20])**.** *We say φ:T→X is piecewise ld-continuous with respect to a sequence $\{t_{k}\}\subset T$ which satisfies $t_{k}<t_{k+1},\;k\in Z$, if φ(t) is continuous on ${\left(t_{k},t_{k+1}\right]}_{T}$ and ld-continuous on $T\setminus \{t_{k}\}$. Furthermore, ${\left(t_{k},t_{k+1}\right]}_{T}$ are called intervals of continuity of the function φ(t).*

For simplicity, let $PC_{ld}\left(T,X\right)$ be the set of all piecewise ld-continuous functions with respect to a sequence $\{t_{k}\},\;k\in Z$ and X be a Banach space. For ${\left\{t_{k}\right\}}_{k\in Z}\in B$, the notation $BPC_{ld}\left(T,X\right)$ denotes the space constituted by all bounded piecewise ld-continuous functions ϕ:T→X with the property that ϕ(·) is ld-continuous at *t* for any $t\notin {\left\{t_{k}\right\}}_{k\in Z}$ and $\phi \left(t_{k}\right)=\phi \left(t_{k}^{-}\right)$ for all k∈Z. The symbol Ω denotes a subset of X and $BPC_{ld}\left(T\times \Omega ,X\right)$ denotes the space constituted by by all bounded piecewise functions which are ld-continuous in *t*, ϕ:T×Ω→X with the property that, for any $x\in \Omega ,\;\phi \left(\cdot ,x\right)\in BPC_{ld}\left(T\times X,X\right)$. Moreover, ϕ(t,·) is continuous at x∈Ω for any t∈T.

Now, we use the symbol $UPC_{ld}\left(T,X\right)$ to denote the space of all functions $\varphi \in PC_{ld}\left(T,X\right)$ with the property that for any ε>0, there exists a positive number δ=δ(ε) such that if the left-dense points $t^{{}^{\prime }},t^{{}^{\prime \prime }}$ belong to the same interval of continuity of φ and $|t^{{}^{\prime }}-t^{{}^{\prime \prime }}|<\delta$, then $∥\varphi \left(t^{{}^{\prime }}\right)-\varphi \left(t^{{}^{\prime \prime }}\right)∥<\varepsilon$.

Furthermore, T,P∈B and s(T∪P):B→B is a map with the property that the set s(T∪P) constitutes a strictly increasing sequence. For D⊂R and ε>0, the notations $\theta_{\varepsilon }\left(D\right)=\left\{t+\varepsilon :t\in D\right\},\;F_{\varepsilon }\left(D\right)=D\cap \left\{\theta_{\varepsilon }\left(D\right)\right\}$. We use the symbol $\tilde{\phi }=\left(\varphi \left(t\right),T\right)$ to denote the element from the space $PC_{ld}\left(T,X\right)\times B$. For every sequence of real numbers $\{s_{n}\},\;n=1,2,\ldots$ with $\theta_{s_{n}}\tilde{\phi }:=\left(\varphi \left(t+s_{n}\right),T-s_{n}\right)$, the sets $\left\{\varphi \left(t+s_{n}\right),T-s_{n}\right\}\subset PC_{ld}\times B$ will be considered, where

$$T-s_{n}=\left\{t_{k}-s_{n}:k\in Z,\;n=1,2,\ldots \right\}.$$

**Definition** **23** ([20])**.** *Let $\{t_{k}\}\in B,\;k\in Z$. We say $\left\{t_{k}^{j}\right\}$ is a derivative sequence of $\left\{t_{k}\right\}$ and*

$$t_{k}^{j}=t_{k+j}-t_{k},\;k,j\in Z.$$

**Definition** **24** ([20])**.** *Let $t_{k}^{j}=t_{k+j}-t_{k},k,j\in Z$. We say $\{t_{k}^{j}\},\;k,j\in Z$ is equipotentially almost automorphic on a periodic time scale T if for any sequence $\{s_{n}\}\subset Z$, there exists a subsequence $\left\{s_{n}^{{}^{\prime }}\right\}$ such that*

$$\lim_{n\to \infty }t_{k}^{s_{n}^{{}^{\prime }}}=\gamma_{k}$$

*is well defined for each k∈Z and*

$$\lim_{n\to \infty }\gamma_{k}^{-s_{n}^{{}^{\prime }}}=t_{k}$$

*for each k∈Z.*

**Definition** **25** ([20])**.** *A function $\phi \in PC_{ld}\left(T,X\right)$ is said to be piecewise ld-continuous almost automorphic (short for ld-piecewise almost automorphic) if the following conditions are fulfilled:*

(*i*) *Let $T=\{t_{k}\}$ be an equipotentially almost automorphic sequence.*
(*ii*) *Let $\varphi \in PC_{ld}\left(T,X\right)$ be a bounded function with respect to a sequence $T=\{t_{k}\}$. We say that φ is ld-piecewise almost automorphic if, from every sequence ${\left\{s_{n}\right\}}_{n=1}^{\infty }\subset \Pi$, we can extract a subsequence ${\left\{\tau_{n}\right\}}_{n=1}^{\infty }$ such that*
  $${\tilde{\phi }}^{*}=\left(\varphi^{*}\left(t\right),T^{*}\right)=\lim_{n\to \infty }\left(\varphi \left(t+\tau_{n}\right),T-\tau_{n}\right)=\lim_{n\to \infty }\theta_{\tau_{n}}\tilde{\phi }$$
  *is well defined for each t∈T and*
  $$\tilde{\phi }=\left(\varphi (t),T\right)=\lim_{n\to \infty }\left(\varphi^{*}\left(t-\tau_{n}\right),T^{*}+\tau_{n}\right)=\lim_{n\to \infty }\theta_{-\tau_{n}}{\tilde{\phi }}^{*}$$
  *for each t∈T. Denote by $AA_{pl}\left(T,X\right)$ the set of all such functions.*
(*iii*) *A bounded function $f\in PC_{ld}\left(T\times X,X\right)$ with respect to a sequence $T=\{t_{k}\}$ is said to be ld-piecewise uniformly almost automorphic if f(t,x) is ld-piecewise automorphic in t∈T uniformly in x∈B, where B is any bounded subset of X. Denote by $AA_{pl}\left(T\times X,X\right)$ the set of all such functions.*

Similarly, we can also introduce the concept of piecewise almost automorphic functions which belong to $PC_{rd}\left(T,X\right)$.

Some basic properties of piecewise almost automorphic functions were obtained as follows.

Let *U* be the set of all functions $\hat{\rho }:T\to \left(0,\infty \right)$ which are positive and locally ∇-integrable over T. For a given $r\in {\left[0,\infty \right)}_{\Pi }$ and $\forall t_{0}\in T$, set

(4) $$m\left(r,\hat{\rho },t_{0}\right):=\int_{t_{0}-r}^{t_{0}+r}\hat{\rho }\left(s\right)\nabla s$$

for each $\hat{\rho }\in U$.

**Remark** **2.** *In* (4)*, if T=R, $t_{0}=0$, one can easily get*

$$m\left(r,\hat{\rho },t_{0}\right):=\int_{-r}^{r}\hat{\rho }\left(s\right)ds$$

*if T=Z, $t_{0}=0$, one has the following:*

$$m\left(r,\hat{\rho },t_{0}\right)=\sum_{k=-r+1}^{r}\hat{\rho }\left(k\right).$$

Define

$$U_{\infty }:=\left\{\hat{\rho }\in U:\lim_{r\to \infty }m\left(r,\hat{\rho },t_{0}\right)=\infty \right\},$$

$$U_{B}:=\left\{\hat{\rho }\in U_{\infty }:\hat{\rho }\;\mathrm{is}\;\mathrm{bounded}\;\mathrm{and}\;\underset{s\in T}{\inf}\hat{\rho }\left(s\right)>0\right\}.$$

It is clear that $U_{B}\subset U_{\infty }\subset U$. Now, for $\hat{\rho }\in U_{\infty }$, define

$$\begin{array}{ccc} PAA_{0}^{pl}\left(T,\hat{\rho }\right): & = & \{\phi \in BPC_{ld}\left(T,X\right):\lim_{r\to \infty }\frac{1}{m(r,\hat{\rho },t_{0})}\int_{t_{0}-r}^{t_{0}+r}∥\phi \left(s\right)∥\hat{\rho }\left(s\right)\nabla s=0,\;\\ & & \forall t_{0}\in T,\;r\in \Pi \}. \end{array}$$

Similarly, we define

$$\begin{array}{ccc} PAA_{0}^{pl}\left(T\times X,\hat{\rho }\right): & = & \{\Phi \in BPC_{ld}\left(T\times \Omega ,X\right):\\ & & \lim_{r\to \infty }\frac{1}{m(r,\hat{\rho },t_{0})}\int_{t_{0}-r}^{t_{0}+r}∥\Phi \left(s,x\right)∥\hat{\rho }\left(s\right)\nabla s=0\;\\ & & \mathrm{uniformly}\;\mathrm{with}\;\mathrm{respect}\;\mathrm{to}\;x\in K,\;\forall t_{0}\in T,\;r\in \Pi \}. \end{array}$$

We are now ready to introduce the sets $WPAA^{pl}\left(T,\hat{\rho }\right)$ and $WPAA^{pl}\left(T\times X,\hat{\rho }\right)$ of piecewise ld-continuous weighted pseudo almost automorphic functions:

$$\begin{array}{c} WPAA^{pl}\left(T,\hat{\rho }\right)=\left\{f=g+\phi \in PC_{ld}\left(T,X\right):g\in AA_{pl}\left(T,X\right)\;\mathrm{and}\;\phi \in PAA_{0}^{pl}\left(T,\hat{\rho }\right)\right\}, \end{array}$$

$$\begin{array}{ccc} WPAA^{pl}\left(T\times X,\hat{\rho }\right) & = & \{f=g+\phi \in PC_{ld}\left(T\times X,X\right):g\in AA_{pl}\left(T\times X,X\right)\;\\ & & \mathrm{and}\;\phi \in PAA_{0}^{pl}\left(T\times X,\hat{\rho }\right)\}. \end{array}$$

**Theorem** **14** ([20])**.** *Let $f=g+\phi \in WPAA^{pl}\left(T\times X,\hat{\rho }\right)$, where $g\in AA_{pl}\left(T\times X,X\right)$, $\phi \in PAA_{0}^{pl}\left(T\times X,\hat{\rho }\right),\;\hat{\rho }\in U_{B}$ and the following conditions hold:*

(*i*) *$\left\{f(t,x):t\in T,x\in K\right\}$ is bounded for every bounded subset K⊆Ω.*
(*ii*) *f(t,·),g(t,·) are uniformly continuous in each bounded subset of *Ω* for all t∈T.*

*Then, $f\left(\cdot ,h(\cdot )\right)\in WPAA^{pl}\left(T,\hat{\rho }\right)$ if $h\in WPAA^{pl}\left(T,\hat{\rho }\right)$ and h(T)⊂Ω.*

**Theorem** **15** ([20])**.** *A necessary and sufficient condition for a bounded sequence $\left\{a_{n}\right\}$ to be in $WPAA^{pl}\left(Z,\hat{\rho }\right)$ is that there exists a uniformly ld-continuous function $f\in WPAA^{pl}\left(T,\hat{\rho }\right)$ and a discretization partition $\left\{t_{n}\right\}$ such that $f\left(t_{n}\right)=a_{n},\;n\in Z,\;\hat{\rho }\in U_{B}$.*

**Theorem** **16** ([20])**.** *Assume that $\hat{\rho }\in U_{B}$ and the sequence of vector-valued functions ${\left\{I_{i}\right\}}_{i\in Z}$ is weighted pseudo almost automorphic, i.e., for any $x\in \Omega ,\;\{I_{i}\left(x\right),i\in Z\}$ is weighted pseudo almost automorphic sequence. Suppose $\left\{I_{i}\left(x\right):i\in Z,x\in K\right\}$ is bounded for every bounded subset K⊆Ω, $I_{i}\left(x\right)$ is uniformly continuous in x∈Ω uniformly in i∈Z. If $h\in WPAA^{pl}\left(T,\hat{\rho }\right)\cap UPC_{ld}\left(T,X\right)$ such that h(T)⊂Ω, then $I_{i}\left(h(t_{i})\right)$ is a weighted pseudo almost automorphic sequence.*

Through using the above basic theorems, one can study the almost automorphic solutions of the following dynamic equations on time scales.

Abstract impulsive ∇-dynamic equations as follows:

(5) $$\left\{\begin{array}{c} x^{\nabla }\left(t\right)=A\left(t\right)x^{\varrho }+f\left(t,x(t)\right),\;\;t\in T,\;t\neq t_{i},\;i\in Z,\\ \Delta x\left(t_{i}\right)=x\left(t_{i}^{+}\right)-x\left(t_{i}^{-}\right)=I_{i}\left(x(t_{i})\right),\;\;t=t_{i}, \end{array}\right.$$

where $A\in PC_{ld}\left(T,X\right)$ is a linear operator in the Banach space X and $f\in PC_{ld}\left(T\times X,X\right),\;x^{\varrho }=x\left(\varrho \left(t\right)\right)$. Now, $f,\;I_{i},\;t_{i}$ satisfy suitable conditions that will be given later and T is an almost periodic time scale. In addition, the notations $x(t_{i}^{+})$ and $x(t_{i}^{-})$ represent the right-hand and the left-hand side limits of x(·) at $t_{i}$, respectively.

In the following, consider the abstract dynamic system (5) with the following assumptions:

(*H*_1_) The family {A(t):t∈T} of operators in X generates an exponentially stable evolution system {T(t,s):t≥s}, i.e., there exist $K_{0}>1$ and ω>0 such that
  $$∥T\left(t,s\right)∥\leq K_{0}{\hat{e}}_{{⊖}_{\nu }\omega }\left(t,s\right),\;\;t\geq s,$$
  and for any sequence $\{s_{n}\}\subset \Pi$, there exists a subsequence $\left\{s_{n}^{{}^{\prime }}\right\}\subset \left\{s_{n}\right\}$ such that
  $$\lim_{n\to \infty }T\left(t+s_{n}^{{}^{\prime }},s+s_{n}^{{}^{\prime }}\right)=T^{*}\left(t,s\right)\;\mathrm{is}\;\mathrm{well}\;\mathrm{defined}\;\mathrm{for}\;\mathrm{each}\;t,s\in T,\;t\geq s.$$
(*H*_2_) $f=g+\phi \in WPAA(T,\hat{\rho })$, where $\hat{\rho }\in U_{\infty }$ and f(t,·) is uniformly continuous in each bounded subset of Ω uniformly in t∈T; $I_{i}$ is a weighted pseudo almost periodic sequence, $I_{i}\left(x\right)$ is uniformly continuous in x∈Ω uniformly in i∈Z, $\inf_{i\in Z}t_{i}^{1}=\theta >0$.

**Theorem** **17** ([20])**.** *Let $f\left(\cdot ,\vartheta (\cdot )\right)\in WPAA\left(T,\hat{\rho }\right)$, where $\vartheta \in WPAA(T,\hat{\rho })$ and {T(t,s),t≥s} is exponentially stable, $\hat{\rho }\in U_{\infty }$. Then,*

$$F\left(\cdot \right):=\int_{-\infty }^{\left(\cdot \right)}T\left(\cdot ,s\right)f\left(s,\vartheta (s)\right)\nabla s+\sum_{t_{i}<\cdot }T\left(\cdot ,t_{i}\right)I_{i}\left(\vartheta (t_{i})\right)\in WPAA\left(T,\hat{\rho }\right).$$

According to Theorem 17, the following existence result of almost automorphic solutions was obtained.

**Theorem** **18** ([20])**.** *Assume the following conditions hold:*

(*A*_1_) *The family {A(t):t∈T} of operators in X generates an exponentially stable evolution system {T(t,s):t≥s}, i.e., there exist $K_{0}>1$ and ω>0 such that*
  $$∥T\left(t,s\right)∥\leq K_{0}{\hat{e}}_{{⊖}_{\nu }\omega }\left(t,s\right),\;\;t\geq s,$$
  *and, for any sequence $\{s_{n}\}\subset \Pi$, there exists a subsequence $\left\{s_{n}^{{}^{\prime }}\right\}\subset \left\{s_{n}\right\}$ such that*
  $$\lim_{n\to \infty }T\left(t+s_{n}^{{}^{\prime }},s+s_{n}^{{}^{\prime }}\right)=T^{*}\left(t,s\right)\;is\;well\;defined\;for\;each\;t,s\in T,\;t\geq s.$$
(*A*_2_) *$f\in WPAA(T\times \Omega ,\hat{\rho })$, and f satisfies the Lipschitz condition with respect to the second argument, i.e.,*
  $$∥f\left(t,x\right)-f\left(t,y\right)∥\leq L_{1}∥x-y∥,\;\;t\in T,\;x,y\in \Omega ,$$
(*A*_3_) *$I_{i}$ is a weighted pseudo almost periodic sequence, and there exists a number $L_{2}>0$ such that*
  $$∥I_{i}\left(x\right)-I_{i}\left(y\right)∥\leq L_{2}∥x-y∥,$$
  *for all x,y∈Ω,i∈Z.*

*Suppose that*

$$\frac{K_{0}L_{1}\left(1-\underline{\nu }\omega \right)}{\omega }+\frac{K_{0}L_{2}}{1-{\hat{e}}_{{⊖}_{\nu }\omega }\left(\theta ,0\right)}<1.$$

*Then, *(5)* has a unique weighted piecewise pseudo almost automorphic mild solution, where ${\hat{e}}_{{⊖}_{\nu }\omega }\left(\theta ,0\right):=\sup_{i\in Z}{\hat{e}}_{{⊖}_{\nu }\omega }\left(t_{i+1},t_{i}\right)$.*

In [21,22], the Π-semigroup and the semigroups induced by complete-closed time scales were introduced to study the almost periodic mild solutions to evolution equations.

Let $\Pi^{+}={\left[0,+\infty \right)}_{\Pi }$ and *X* be a Banach space, and $T_{\tau }:X\to X$ be a transformation. Obviously, $\left\{T_{\tau }:\tau \in \Pi \right\}$ is a set containing only one parameter. We introduce the multiplication as follows:

(6) $$T_{\tau_{1}}T_{\tau_{2}}=T_{\tau_{1}+\tau_{2}}.$$

It follows that

$$T_{\tau_{1}}\left(T_{\tau_{2}}T_{\tau_{3}}\right)=\left(T_{\tau_{1}}T_{\tau_{2}}\right)T_{\tau_{3}}=T_{\tau_{1}+\tau_{2}+\tau_{3}},$$

$I=T_{0}$ is the identity, and $T_{-\tau }$ is the inverse element of $T_{\tau }.$

**Theorem** **19** ([21])**.** *$\left\{T_{\tau }:\tau \in \Pi \right\}$ is an operator group with respect to the multiplication defined by *(6)*. It is an Abelian group.*

According to Theorem 19, some basic concepts which will be needed to define a Π-semigroup for an invariant time scale under translations can be introduced as follows.

**Definition** **26** ([21])**.** *Let a time scale T be invariant under translations, and $\left\{T_{\tau }\right\}$ be a family of bounded linear operators on Banach space X. If, for all $\tau_{1},\tau_{2}\in \Pi^{+}$, the following holds:*

(7) $$T_{\tau_{1}+\tau_{2}}=T_{\tau_{1}}T_{\tau_{2}},$$

*then $\left\{T_{\tau }:\tau \in \Pi^{+}\right\}$ is called a one-parameter operator semigroup; if *(7)* holds for all τ∈Π, we call $\left\{T_{\tau }:\tau \in \Pi \right\}$ a one-parameter operator group.*

**Definition** **27** ([21])**.** *Let T be an invariant time scale under translations, and $\left\{T_{\tau }:\tau \in \Pi^{+}\right\}$ be an operator group on a Banach space X, i.e.,*

$$T_{\tau_{1}}T_{\tau_{2}}=T_{\tau_{1}+\tau_{2}},\;\tau_{1},\tau_{2}\in \Pi^{+},\;T_{0}=I.$$

*If, for every $\tau_{0}\geq 0$ and any ε>0, there is a neighborhood U of $\tau_{0}$(i.e., $U={\left(\tau_{0}-\delta ,\tau_{0}+\delta \right)}_{\Pi^{+}}$ for some δ>0) such that*

$$∥T_{\tau }x-T_{\tau_{0}}x∥<\varepsilon \;\mathrm{for}\;\mathrm{all}\;\tau \in U,$$

*then we call $\left\{T_{\tau }:\tau \in \Pi^{+}\right\}$ the strong-continuous operator semigroup or the *Π*-semigroup.*

**Theorem** **20** ([21])**.** *Let a time scale T be invariant under translations, and $\left\{T_{\tau }:\tau \in \Pi^{+}\right\}$ be an operator semigroup on the Banach space X. For any ε>0 and x∈X, there exists a neighborhood $U={\left(\tau_{1}-\delta ,\tau_{1}+\delta \right)}_{\Pi^{+}}$ for some δ>0, such that*

(8) $$∥T_{|\sigma_{\Pi }\left(\tau_{1}\right)-\tau_{2}|}x-x∥\leq \varepsilon \;for\;all\;\tau_{2}\in U,$$

*then $\left\{T_{\tau }:\tau \in \Pi^{+}\right\}$ is a *Π*-semigroup.*

In the following, the definition of infinitesimal generator of a Π-semigroup was introduced.

**Definition** **28** ([21])**.** *Let T be an invariant time scale under translations and $\left\{T_{\tau }:\tau \in \Pi^{+}\right\}$ be a *Π*-semigroup on a Banach space X. Let D denote a subset of X, which has the property that, for each x∈D, there exists a y∈X such that for any ε>0, there is a neighborhood $U={\left(\tau_{1}-\delta ,\tau_{1}+\delta \right)}_{\Pi^{+}}$ for some δ>0 such that*

(9) $$∥\left(T_{|\sigma_{\Pi }\left(\tau_{1}\right)-\tau_{2}|}-I\right)x-y|\sigma_{\Pi }\left(\tau_{1}\right)-\tau_{2}\left|∥<\varepsilon \right|\sigma_{\Pi }\left(\tau_{1}\right)-\tau_{2}|,\;\tau_{2}\in U.$$

*We define A:D→X satisfying Ax=y, where y is fixed by *(9)*. In what follows, we call this A the infinitesimal generator of this *Π*-semigroup.*

**Theorem** **21** ([21])**.** *Let T be an invariant under translations time scale, $\left\{T_{\tau }:\tau \in \Pi^{+}\right\}$ be a *Π*-semigroup on Banach space X satisfying *(8)*, and A be the infinitesimal generator of the *Π*-semigroup. Then, A is a closed densely defined operator and for every x∈D(A), the following holds:*

$${\left(T_{\tau }x\right)}^{\Delta_{\Pi }}=A\left(T_{\tau }x\right)=T_{\tau }Ax,$$

*that is*

$$\left(T_{\tau }x\right)-x=\int_{0}^{\tau }AT_{s}x\Delta_{\Pi }s=\int_{0}^{\tau }T_{s}Ax\Delta_{\Pi }s,$$

*where D(A) denotes the domain of the operator A and $\Delta_{\Pi }$ is the differential operator over the time scale *Π*.*

**Theorem** **22** ([21])**.** *Let T be an invariant time scale under translations and X be a Banach space. Assume that $\left\{T_{\tau }:\tau \in \Pi^{+}\right\}$ is a *Π*-semigroup, A is the infinitesimal generator of the *Π*-semigroup and D(A)=X,$e_{A}\left(\tau_{1}+\tau_{2},0\right)=e_{A}\left(\tau_{1},0\right)e_{A}\left(\tau_{2},0\right)$ for all $\tau_{1},\tau_{2}\in \Pi^{+}.$ Then,*

$$T_{\tau }=e_{A}\left(\tau ,0\right),\;\;\tau \in \Pi^{+},$$

*where D(A) denotes the domain of A.*

Now, we introduce a new notion called the moving-operator on time scales.

**Definition** **29** ([21])**.** *Let A be the infinitesimal generator of the *Π*-semigroup. We call ${\tilde{e}}_{A}\left(t,t_{0}\right),t_{0}\in T$ the exponential function generated by A on the time scale T. We also let $T_{t}={\tilde{e}}_{A}\left(t,t_{0}\right)$ and call $T_{t}$ the moving-operator on T.*

Let *X* be a Banach space, and consider the following system:

(10) $$x^{\Delta }=Ax\left(t\right),\;x\left(t_{0}\right)=x_{0},\;\;t_{0}\in T,$$

where *A* is the infinitesimal generator of a Π-semigroup satisfying all the conditions in Theorem 22, and x:T→X.

**Theorem** **23** ([21])**.** *The fundamental solution of the system *(10)* can be expressed as*

$$x\left(t\right)=T_{t}x_{0},$$

From Theorem 23, the following result follows immediately.

**Theorem** **24** ([21])**.** *Let A be the infinitesimal generator of the *Π*-semigroup, and let $T_{t}$ be the moving-operator on T. Then,*

$${\left(T_{t}x\right)}^{\Delta }=A\left(T_{t}x\right)=T_{t}Ax,$$

*that is*

$$\left(T_{t}x\right)-x=\int_{t_{0}}^{t}AT_{s}x\Delta s=\int_{t_{0}}^{t}T_{s}Ax\Delta s.$$

In the following part, we will introduce two equivalent definitions of relatively dense sets on semigroups induced by complete-closed time scales under translations.

**Definition** **30** ([22])**.** *Let T be a complete-closed time scale. If*

$$\Pi^{+}:={\left[0,+\infty \right)}_{\Pi }\notin \left\{\emptyset ,\{0\}\right\},$$

*then we say $\left(\Pi^{+},+\right)$ is a positive direction semigroup induced by the time scale T; if*

$$\Pi^{-}:={\left(-\infty ,0\right]}_{\Pi }\notin \left\{\emptyset ,\{0\}\right\},$$

*then we say $\left(\Pi^{-},+\right)$ is a negative direction semigroup induced by the time scale T.*

Now, we denote the set {1,2,…,m} by Λ and introduce the following concept.

**Definition** **31** ([22])**.** *A subset E of a semigroup $\Pi^{+}$ induced by time scales is relatively dense if there exists elements $s_{1},s_{2},\ldots ,s_{m}$ in $\Pi^{+}$ such that ${⋃}_{i\in \Lambda }\left(s_{i}+E\right)=\Pi^{+}$, where $s_{i}+E=\left\{s_{i}+e:e\in E\right\}$.*

**Definition** **32** ([22])**.** *A subset E of $\Pi^{+}$ is called relatively dense if there exists a positive number $L\in \Pi^{+}$ such that ${\left[a,a+L\right]}_{\Pi^{+}}\cap E\neq \emptyset$ for all $a\in \Pi^{+}$. The number L is called the inclusion length.*

**Theorem** **25** ([22])**.** *Definition 31 is equivalent to Definition 32.*

By Theorem 25, it is obvious that, for the Abelian group (Π,+), the following two definitions are also equivalent.

**Definition** **33** ([22])**.** *A subset E of a group *Π* induced by time scales is relatively dense if there exists elements $s_{1},s_{2},\ldots ,s_{m}$ in *Π* such that ${⋃}_{i\in \Lambda }\left(s_{i}+E\right)=\Pi$, where $s_{i}+E=\left\{s_{i}+e:e\in E\right\}$.*

**Definition** **34** ([22])**.** *A subset E of *Π* is called relatively dense if there exists a positive number $L\in \Pi^{+}$ such that ${\left[a,a+L\right]}_{\Pi }\cap E\neq \emptyset$ for all a∈Π. The number L is called the inclusion length.*

Next, in [22], the equivalence of Bochner and Bohr almost automorphy on semigroup related to time scales was proved which play a fundamental role in studying the almost automorphic solutions for dynamic equations by using both notions.

**Definition** **35** ([22])**.** *Let T be a positive direction complete-closed time scale and $\left(\Pi^{+},+\right)$ be a semigroup. A function f:T→X is said to be almost automorphic function on the semigroup $\left(\Pi^{+},+\right)$ if for any sequence $\alpha^{{}^{\prime }}={\left\{\alpha_{n}^{{}^{\prime }}\right\}}_{n\in N}\subset \Pi^{+}$ of semigroup elements, there is a subsequence $\alpha ={\left\{\alpha_{n}\right\}}_{n\in N}$ and a sequence $\left\{{\tilde{\alpha }}_{n}\right\}\subset \Pi^{+}$ depending on α such that for each t∈T the equality*

$$\lim_{n\to \infty }\lim_{m\to \infty }f\left(t+\alpha_{m}+{\tilde{\alpha }}_{n}\right)=T_{\tilde{\alpha }}T_{\alpha }f=f\left(t\right)$$

*holds on $\left(\Pi^{+},+\right)$.*

**Definition** **36** ([22])**.** *A bounded function f on a semigroup $\Pi^{+}$ is said to be positive direction Bohr almost automorphic if, for each finite set $N_{T}\subset T$ and prescribed ε>0, there is a set $B_{\varepsilon }=B_{\varepsilon }\left(N_{T}\right)\subset \Pi^{+}$ such that*

(*i*) *$B_{\varepsilon }$ is relatively dense.*
(*ii*) *If $\tau \in B_{\varepsilon }$, then $\max_{t\in N_{T}}\left|f\left(t+\tau \right)-f\left(t\right)\right|<\varepsilon .$*
(*iii*) *If $\tau_{1},\tau_{2}\in B_{\varepsilon }$, then $\max_{t\in N_{T}}\left|f\left(t+\tau_{1}+\tau_{2}\right)-f\left(t\right)\right|<2\varepsilon .$*

**Theorem** **26** ([22])**.** *A function f on semigroup $\Pi^{+}$ is a positive direction Bochner almost automorphic function if and only if it is a positive direction Bohr almost automorphic function.*

Particularly, since the irregularity of time scales, the delay classification was addressed to solve the delay dynamic equations on hybrid time scales (see [23]).

The irregularity and the translation of time scales led to the idea of the approximation of time scales. In 2014, Wang and Agarwal (see [24]) firstly proposed the concept of almost periodic time scales with the approximation property as follows:

**Definition** **37** ([11,12,13])**.** *We say T is an almost periodic time scale, if for any given ε>0, there exists a constant l(ε)>0 such that each interval of length l(ε) contains a τ(ε)∈R such that $d(T,T^{\tau })<\varepsilon$, i.e., for any ε>0, the following set*

$$E\left\{T,\varepsilon \right\}=\{\tau \in R:d\left(T^{\tau },T\right)\leq \varepsilon \}$$

*is relatively dense in $\Pi_{1}$. Here, τ is called the ε-translation number of T and l(ε) is called the inclusion length of E{T,ε}, E{T,ε} is called the ε-translation numbers set of T, and for simplicity, we use the notation $E\left\{T,\varepsilon \right\}:=\Pi_{\varepsilon }$ and $\Pi_{1}:=\left\{\tau \in R:T\cap T^{\tau }\neq \emptyset \right\}\neq \left\{0\right\},$ where $T^{\tau }:=T+\tau =\left\{t+\tau :\forall t\in T\right\}$.*

Definition 37 was applied to study the almost periodicity and almost automorphy of time scales through translations and the notions of almost periodic and almost automorphic time scales were introduced (see [25]). Based on the results of approximation property of time scales, a new type of almost periodic functions called double-almost periodic functions was proposed and applied to study neural networks and biological dynamic models, and some new results of the existence and stability of the double-almost periodic solutions were established (see [26,27]). Moreover, these results were also extended to discontinuous cases and some notions of piecewise double-almost periodic functions and their generalizations were put forward and applied to study the impulsive dynamic equations and models (see [28,29,30,31]).

In 2015, to obtain the general results on more complicated hybrid time scales, the notion of changing-periodic time scales was introduced as follows:

**Definition** **38** ([32,33])**.** *Let T be an infinite time scale. We say T is a changing-periodic or a piecewise-periodic time scale if the following conditions are fulfilled:*

(*a*) *$T=\left(\overset{\infty }{\underset{i=1}{⋃}}T_{i}\right)⋃T_{r}$ and ${\left\{T_{i}\right\}}_{i\in Z^{+}}$ is a well connected timescale sequence, where $T_{r}=\overset{k}{\underset{i=1}{⋃}}\left[\alpha_{i},\beta_{i}\right]$ and k is some finite number, and $\left[\alpha_{i},\beta_{i}\right]$ are closed intervals for i=1,2,…,k or $T_{r}=\emptyset$;*
(*b*) *$S_{i}$ is a nonempty subsets of R with $0\notin S_{i}$ for each $i\in Z^{+}$ and $\Lambda =\left({⋃}_{i=1}^{\infty }S_{i}\right)⋃R_{0}$, where $R_{0}=\left\{0\right\}$ or $R_{0}=\emptyset$;*
(*c*) *for all $t\in T_{i}$ and all $\omega \in S_{i}$, we have $t+\omega \in T_{i}$, i.e., $T_{i}$ is an ω-periodic time scale;*
(*d*) *for i≠j, for all $t\in T_{i}\setminus \left\{t_{ij}^{k}\right\}$ and all $\omega \in S_{j}$, we have t+ω∉T, where $\left\{t_{ij}^{k}\right\}$ is the connected points set of the timescale sequence ${\left\{T_{i}\right\}}_{i\in Z^{+}}$;*
(*e*) *$R_{0}=\left\{0\right\}$ if and only if $T_{r}$ is a zero-periodic time scale and $R_{0}=\emptyset$ if and only if $T_{r}=\emptyset$;*

*and the set *Λ* is called a changing-periods set of T, $T_{i}$ is called the periodic sub-timescale of T and $S_{i}$ is called the periods subset of T or the periods set of $T_{i}$, $T_{r}$ is called the remain time scale of T and $R_{0}$ the remain periods set of T.*

Definition 38 shows that one can discuss the almost periodic and almost automorphic approximation problems on any arbitrary time scales with a bounded graininess function μ. The following theorems play a fundamental role in establishing the basic theory of local almost periodic and almost automorphic functions and the related dynamic equations on time scales. Based on the following theorems, it is meaningful to conduct the related qualitative analysis of local almost periodic and almost automorphic dynamical behavior described by dynamic systems on arbitrary time scales in the future.

**Theorem** **27** ([32,33], Decomposition Theorem of Time Scales)**.** *Let T be an infinite time scale and the graininess function $\mu :T\to R^{+}$ be bounded. Then, T is a changing-periodic time scale, i.e., there exists a countable periodic decomposition such that $T=\left(\overset{\infty }{\underset{i=1}{⋃}}T_{i}\right)⋃T_{r}$ and $T_{i}$ is ω-periodic sub-timescale, $\omega \in S_{i},\;i\in Z^{+}$, where $T_{i},\;S_{i},\;T_{r}$ satisfy the conditions in Definition 38.*

**Theorem** **28** ([32,33], Periodic Coverage Theorem of Time Scales)**.** *Let T be an infinite time scale and the graininess function $\mu :T\to R^{+}$ be bounded. Then, T can be covered by countable periodic time scales.*

On changing-periodic time scales, the local-periodic solutions for functional dynamic equations with infinite delay and the local pseudo almost automorphic solutions to semilinear dynamic equations were respectively discussed (see [34,35]).

Consider the following dynamic equation:

(11) $$x^{\Delta }\left(t\right)=Ax\left(t\right)+f\left(t,x(t)\right),\;\;t\in T,$$

where *A* is the infinitesimal generator of a Π-semigroup for the periodic sub-timescale $T_{\tau_{t}}$, $x:T_{\tau_{t}}\to X,\;f:T_{\tau_{t}}\times X\to X.$

**Definition** **39** ([35])**.** *A local mild solution to *(11)* is a continuous function $x\left(t\right):T_{\tau_{t}}\to X$ satisfying*

$$x\left(t\right)=T_{t,t_{0}}^{\tau }x\left(t_{0}\right)+\int_{t_{0}}^{t}T_{t,s}^{\tau }f\left(s,x(s)\right)\Delta_{\tau_{s}}s$$

*for all $t\geq t_{0}$ and all $t_{0}\in T_{\tau_{t}},$ where $T_{t,t_{0}}^{\tau }$ is the moving-operator on $T_{\tau_{t}}$.*

In [35], the following sufficient condition of the existence and uniqueness of the local pseudo almost automorphic mild solution to (11) was established under the following assumptions:

(*H*_1_) Let *A* be the infinitesimal generator of a Π-semigroup $\left\{T_{\tau }:\tau \in S_{\tau_{t}}\right\}$. The moving-operator family $\left\{T_{t,t_{0}}^{\tau }:t,t_{0}\in T_{\tau_{t}},t\geq t_{0}\right\}$ is exponentially stable, that is, there exist K>0, ω>0 such that
  $$∥T_{t,t_{0}}^{\tau }∥\leq Ke_{⊖\omega }^{\tau }\left(t,t_{0}\right),\;\mathrm{for}\;\mathrm{all}\;t\in T_{\tau_{t}}.$$
(*H*_2_) f:R×X→X is local pseudo almost automorphic.
(*H*_3_) There exists a nonnegative function $\varrho_{0}\left(t\right)\in L^{p}\left(T_{\tau_{t}},R^{+}\right)\left(p=1,2\right)$ such that
  $$∥f\left(t,x\right)-f\left(t,y\right)∥\leq \varrho_{0}\left(t\right)∥x-y∥$$
  for all x,y∈X and $t\in T_{\tau_{t}}.$

**Theorem** **29** ([35])**.** *Under assumption $\left(H_{1}\right)-\left(H_{3}\right)$, if $S_{\tau_{t}}^{-}\neq \left\{0\right\}$ or $S_{\tau_{t}}^{+}\notin \left\{\{0\},\emptyset \right\}$, then *(11)* has a unique local pseudo almost automorphic mild solution.*

### 2.2. Almost Periodic and Almost Automorphic Theory under Matched Spaces of Time Scales

In 2017, the notion of matched spaces of time scales was introduced by Wang and Agarwal et al. in [36,37,38]. Before giving the concept of matched spaces of time scales, we need the following definition.

**Definition** **40** ([36,38])**.** *Let the pair $\left(\Pi^{*},\tilde{\delta }\right)$ be an Abelian group and $\Pi^{*},\;T^{*}$ be the largest open subsets of the time scales *Π* and T, respectively. Furthermore, let *Π* be the adjoint set of T and F the adjoint mapping between T and *Π*. The operator $\delta :\Pi^{*}\times T^{*}\to T^{*}$ satisfies the following properties:*

(*P*_1_) *(Monotonicity) The function δ is strictly increasing with respect to its all arguments, i.e., if*
  $$\left(T_{0},t\right),\;\left(T_{0},u\right)\in D_{\delta }:=\left\{\left(s,t\right)\in \Pi^{*}\times T^{*}:\delta \left(s,t\right)\in T^{*}\right\},$$
  *then t<u implies $\delta \left(T_{0},t\right)<\delta \left(T_{0},u\right)$; if $\left(T_{1},u\right),\;\left(T_{2},u\right)\in D_{\delta }$ with $T_{1}<T_{2}$, then $\delta \left(T_{1},u\right)<\delta \left(T_{2},u\right)$.*
(*P*_2_) *(Existence of inverse elements) The operator δ has the inverse operator $\delta^{-1}:\Pi^{*}\times T^{*}\to T^{*}$ and $\delta^{-1}\left(\tau ,t\right)=\delta \left(\tau^{-1},t\right)$, where $\tau^{-1}\in \Pi^{*}$ is the inverse element of τ.*
(*P*_3_) *(Existence of identity element) There exists $e_{\Pi^{*}}\in \Pi^{*}$ such that $\delta (e_{\Pi^{*}},t)=t$ for any $t\in T^{*}$, where $e_{\Pi^{*}}$ is the identity element in $\Pi^{*}$.*
(*P*_4_) *(Bridge condition) For any $\tau_{1},\tau_{2}\in \Pi^{*}$ and $t\in T^{*}$, $\delta \left(\tilde{\delta }\left(\tau_{1},\tau_{2}\right),t\right)=\delta \left(\tau_{1},\delta \left(\tau_{2},t\right)\right)=\delta \left(\tau_{2},\delta \left(\tau_{1},t\right)\right)$.*

*Then, the operator δ(s,t) associated with $e_{\Pi^{*}}\in \Pi^{*}$ is said to be a shift operator on the set $T^{*}$. The variable $s\in \Pi^{*}$ in δ is called the shift size. The value δ(s,t) in $T^{*}$ indicates s units shift of the term $t\in T^{*}$. The set $D_{\delta }$ is the domain of the shift operator δ.*

Then, the matched spaces of time scales can be defined as follows.

**Definition** **41** ([36,38])**.** *Let the pair $\left(\Pi^{*},\tilde{\delta }\right)$ be an Abelian group, and $\Pi^{*},\;T^{*}$ be the largest open subsets of the time scales *Π* and T, respectively. Furthermore, let *Π* be an adjoint set of T and F the adjoint mapping between T and *Π*. If there exists the shift operator δ satisfying Definition 40, then we say the group (T,Π,F,δ) is a matched space for the time scale T.*

By using Definition 41, the classical definitions of almost periodic functions and almost automorphic functions can be generalized as follows.

**Definition** **42** ([39])**.** *Let T be a periodic time scale under the matched space (T,Π,F,δ). A function f∈C(T×D,X) is called δ-almost periodic function with shift operators in t∈T uniformly for x∈D if the ε-shift set of f*

$$E\left\{\varepsilon ,f,S\right\}=\{\tau \in \tilde{\Pi }:∥f\left(\delta_{\tau^{\pm 1}}\left(t\right),x\right)-f\left(t,x\right)∥<\varepsilon ,\;\;for\;all\;t\in T^{*}\;and\;x\in S\}$$

*is a relatively dense set with respect to the pair $\left(\Pi^{*},\tilde{\delta }\right)$ for all ε>0 and for each compact subset S of D; that is, for any given ε>0 and each compact subset S of D, there exists a constant l(ε,S)>0 such that each interval of length l(ε,S) contains a τ(ε,S)∈E{ε,f,S} such that*

$$∥f\left(\delta_{\tau^{\pm }}\left(t\right),x\right)-f\left(t,x\right)∥<\varepsilon ,\;for\;all\;t\in T^{*}\;and\;x\in S.$$

*Now, τ is called the ε-shift number of f and l(ε,S) is called the inclusion length of E{ε,f,S}.*

**Definition** **43** ([40])**.** (*i*) *Let f:T→X be a bounded continuous function. f is said to be δ-almost automorphic under the matched space (T,F,Π,δ) if for every sequence of real numbers ${\left\{s_{n}\right\}}_{n=1}^{\infty }\subset \tilde{\Pi },$ one can extract a subsequence ${\left\{\tau_{n}\right\}}_{n=1}^{\infty }\subset \tilde{\Pi }$ such that:*

$$g\left(t\right)=\lim_{n\to \infty }f\left(\delta_{\tau_{n}}\left(t\right)\right)$$

*is well defined for each t∈T and*

$$\lim_{n\to \infty }g\left(\delta_{\tau_{n}^{-1}}\left(t\right)\right)=\lim_{n\to \infty }g\left(\delta_{\tau_{n}}^{-1}\left(t\right)\right)=f\left(t\right)$$

*for each t∈T. Denote by $AA^{\delta }\left(T,X\right)$ the set of all such functions.*

(*ii*) *A continuous function f:T×X→X is said to be δ-almost automorphic if f(t,x) is δ-almost automorphic in t∈T uniformly for all x∈B, where B is any bounded subset of X. Denote by $AA^{\delta }\left(T\times X,X\right)$ the set of all such functions.*

Definitions 42 and 43 are the basic concepts of almost periodic functions and almost automorphic functions on irregular time scales such as $\bar{q^{Z}},N^{\pm \frac{1}{2}}$, etc., and their basic properties were obtained as follows.

**Theorem** **30** ([36,38,39])**.** *Assume that $f\in C(T\times D,E^{n})$ is δ-almost periodic in t uniformly for x∈D under the matched space (T,F,Π,δ), and $\delta_{\tau }\left(t\right)$ is continuous in t. Then, it is uniformly continuous and bounded on $T^{*}\times S$.*

We introduce the moving-operator $T^{\delta }$, $T_{\alpha }^{\delta }f\left(t,x\right)=g\left(t,x\right)$ by

$$g\left(t,x\right)=\lim_{n\to +\infty }f\left(\delta_{\alpha_{n}}\left(t\right),x\right)$$

and is written only when the limit exists. The mode of convergence, e.g., pointwise, uniform, etc., will be specified at each use of the symbol.

In the following, we will establish a shift-convergence theorem of δ-almost periodic functions.

**Theorem** **31** ([36,38,39])**.** *Assume that $f\in C(T\times D,E^{n})$ is δ-almost periodic in t uniformly for x∈D under the matched space (T,F,Π,δ). Then, for any given sequence $\alpha^{{}^{\prime }}\subset \tilde{\Pi },$ there is a subsequence $\beta \subset \alpha^{{}^{\prime }}$ and $g\in C(T\times D,E^{n})$ such that $T_{\beta }^{\delta }f\left(t,x\right)=g\left(t,x\right)$ holds uniformly on $T^{*}\times S$. Furthermore, g(t,x) is δ-almost periodic in t uniformly for x∈D under the matched space (T,F,Π,δ).*

**Theorem** **32** ([36,38,39])**.** *Assume that $f\left(t,x\right)\in C\left(T\times D,E^{n}\right)$ is δ-almost periodic in t uniformly for x∈D and φ(t) is δ-almost periodic with {φ(t):t∈T}⊂S, then $f\left(t,\varphi (t)\right)$ is δ-almost periodic.*

**Definition** **44** ([36,38,39])**.** *Let $f\left(t,x\right)\in C\left(T\times D,E^{n}\right)$. Then, $H_{\delta }\left(f\right)=\left\{g\left(t,x\right):T\to E^{n}\right|$ there is $\alpha \in \tilde{\Pi }$ such that $T_{\alpha }^{\delta }f\left(t,x\right)=g\left(t,x\right)$ exists uniformly on $T^{*}\times S$*}* is said to be the δ-hull of f(t,x) under the matched space (T,F,Π,δ).*

**Theorem** **33** ([36,38,39])**.** *$H_{\delta }\left(f\right)$ is compact if and only if f(t,x) is δ-almost periodic in t uniformly for x∈D.*

**Theorem** **34** ([36,38,39])**.** *If f(t,x) is δ-almost periodic in t uniformly for x∈D under the matched space (T,F,Π,δ), then for any $g\left(t,x\right)\in H_{\delta }\left(f\right)$ and $H_{\delta }\left(f\right)=H_{\delta }\left(g\right)$.*

Based on the theorems above, a sufficient and necessary criterion for δ-almost periodic functions was established.

**Theorem** **35** ([36,38,39])**.** *A function f(t,x) is δ-almost periodic in t uniformly for x∈D under the matched space (T,F,Π,δ) if and only if for every pair of sequences $\alpha^{{}^{\prime }},\beta^{{}^{\prime }}\subseteq \tilde{\Pi }$, there exist common subsequences $\alpha \subset \alpha^{{}^{\prime }},\beta \subset \beta^{{}^{\prime }}$ such that*

(12) $$T_{\tilde{\delta }\left(\alpha ,\beta \right)}^{\delta }f\left(t,x\right)=T_{\alpha }^{\delta }T_{\beta }^{\delta }f\left(t,x\right).$$

In what follows, some basic properties of δ-almost automorphic functions were also established.

Next, the notation X denotes a Banach space endowed with the norm ∥·∥ and B(X,Y) the Banach space of all bounded linear operators from X to Y. This is simply denoted as B(X) when X=Y. Let BC(T,X) be the space of bounded continuous function from T to X with the supremum norm ${∥u∥}_{\infty }=\sup_{t\in T}∥u\left(t\right)∥.$

**Theorem** **36** ([40,41])**.** *$AA^{\delta }\left(T,X\right)$ equipped with the norm ${∥\cdot ∥}_{\infty }$ is a Banach space.*

**Theorem** **37** ([40,41])**.** *Let (T,F,Π,δ) be a regular matched space. If $g\left(t,x\right)\in AA^{\delta }\left(T\times X,X\right)$ and $\alpha \left(t\right)\in AA^{\delta }\left(T,X\right)$, then $G\left(t\right):=g\left(t,\alpha (t)\right)\in AA^{\delta }\left(T,X\right)$.*

Moreover, if the following assumptions hold:

(H1) f(t,x) is uniformly continuous in any bounded subset K⊂X for all t∈T.
(H2) g(t,x) is uniformly continuous in any bounded subset K⊂X for all t∈T.

Then, we can obtain the following theorem.

**Theorem** **38** ([40,41])**.** *Let $f=g+\phi \in WPAA^{\delta }\left(T\times X,\rho \right)$ where $g\in AA^{\delta }\left(T\times X,X\right)$, $\phi \in PAA_{0}^{\delta }\left(T\times X,\rho \right),\;\rho \in U_{\infty }$. Assume that (H1) and (H2) are satisfied. Then, the $L\left(\cdot \right):=f\left(\cdot ,h(\cdot )\right)\in WPAA^{\delta }\left(T,\rho \right)$ if $h\in WPAA^{\delta }\left(T,\rho \right)$.*

From Theorem 38, we can establish the following consequence:

**Corollary** **1** ([40,41])**.** *Let $f=g+\phi \in WPAA^{\delta }\left(T,\rho \right)$ where $\rho \in U_{\infty }$ and assume both f and g are Lipschitzian in x∈X uniformly in t∈T. Then $L\left(\cdot \right):=f\left(\cdot ,h(\cdot )\right)\in WPAA^{\delta }\left(T,\rho \right)$ if $h\in WPAA^{\delta }\left(T,\rho \right).$*

It is very important to establish the approximation theory on non-translational shift time scales since that they may combine into more complicated hybrid time scales. In [38,41], the concept of the $n_{0}$-order Δ-almost periodic functions and weighted pseudo δ-almost automorphic functions were introduced and studied, respectively, and their obtained basic properties were applied to the qualitative analysis of the related dynamic equations on hybrid domains.

**Definition** **45** ([38])**.** *Let T be a periodic time scale under the matched space (T,Π,F,δ) and $n_{0}\in N$, the shift $\delta_{\tau }\left(t\right)$ is *Δ*-differentiable with rd-continuous bounded derivatives $\delta_{\tau }^{\Delta }\left(t\right):=\delta^{\Delta }\left(\tau ,t\right)$ for all $t\in T^{*}$. A function f∈C(T×D,X) is called an* **$n_{0}$-*order*** **Δ*-almost periodic function ($\Delta_{n_{0}}^{\delta }$-almost periodic function) in t∈T uniformly for x∈D under the matched space if there exists some $i_{0}\geq 1,\;n_{i}\in Z,\;i=1,2,\ldots ,i_{0}$ such that the ε-shift set of $S_{f}^{\bar{n_{1},n_{i_{0}}}}$*

$$E\left\{\varepsilon ,S_{f}^{\bar{n_{1},n_{i_{0}}}},S\right\}=\left\{\tau \in \tilde{\Pi }:∥f\left(\delta_{\tau }\left(t\right),x\right){\left(\delta_{\tau }^{\Delta }\left(t\right)\right)}^{n_{0}}-S_{f}^{\bar{n_{1},n_{i_{0}}}}\left(t,x\right)∥<\varepsilon ,\;\;for\;all\;t\in T^{*}\;and\;x\in S\right\}$$

*is a relatively dense set with respect to the pair $\left(\Pi^{*},\tilde{\delta }\right)$ for all ε>0 and, for each compact subset S of D; that is, there exists some $i_{0}\geq 1,\;n_{i}\in Z,\;i=1,2,\ldots ,i_{0}$ such that for any given ε>0 and each compact subset S of D, there exists a constant l(ε,S)>0 such that each interval of length l(ε,S) contains a $\tau \left(\varepsilon ,S\right)\in E\left\{\varepsilon ,S_{f}^{\bar{n_{1},n_{i_{0}}}},S\right\}$ such that*

$$∥f\left(\delta_{\tau }\left(t\right),x\right){\left(\delta_{\tau }^{\Delta }\left(t\right)\right)}^{n_{0}}-S_{f}^{\bar{n_{1},n_{i_{0}}}}\left(t,x\right)∥<\varepsilon ,\;for\;all\;t\in T^{*}\;and\;x\in S,$$

*where*

$$S_{f}^{\bar{n_{1},n_{i_{0}}}}\left(t,x\right)=f\left(t,x\right)\prod_{i=1}^{i_{0}}{\left(\delta_{e_{\Pi^{*}}}^{\Delta }\left(t\right)\right)}^{n_{i}}.$$

*Now, τ is called the ε-shift number of $S_{f}^{\bar{n_{1},n_{i_{0}}}}$ and l(ε,S) is called the inclusion length of $E\{\varepsilon ,S_{f}^{\bar{n_{1},n_{i_{0}}}},S\}$, and $S_{f}^{\bar{n_{1},n_{i_{0}}}}$ is called the approximation shift selection-function (ASS-function) of f.*

In what follows, we established some basic properties of $\Delta_{n_{0}}^{\delta }$-almost periodic functions.

**Theorem** **39** ([38])**.** *Let $f\in C(T\times D,E^{n})$ be $\Delta_{n_{0}}^{\delta }$-almost periodic in t uniformly for x∈D with the ASS-function $S_{f}^{n_{0}}=f\left(t,x\right){\left(\delta_{e_{\Pi^{*}}}^{\Delta }\left(t\right)\right)}^{n_{0}}$ under the matched space (T,F,Π,δ), and $\delta_{\tau }\left(t\right)$ is continuous in t. Then, $S_{f}^{n_{0}}$ is uniformly continuous and bounded on $T^{*}\times S$.*

In the following, we established a shift-convergence theorem of $\Delta_{n_{0}}^{\delta }$-almost periodic functions.

**Theorem** **40** ([38])**.** *Let $f\in C(T\times D,E^{n})$ be $\Delta_{n_{0}}^{\delta }$-almost periodic in t uniformly for x∈D with the ASS-function $S_{f}^{n_{0}}=f\left(t,x\right){\left(\delta_{e_{\Pi^{*}}}^{\Delta }\left(t\right)\right)}^{n_{0}}$ under the matched space (T,F,Π,δ). Then, for any given sequence $\alpha^{{}^{\prime }}\subset \tilde{\Pi },$ there exists a subsequence $\beta \subset \alpha^{{}^{\prime }}$ and $g\in C(T\times D,E^{n})$ such that $T_{\beta }^{\delta ,n_{0}}\left(S_{f}^{n_{0}}\right)=S_{g}^{n_{0}}$ holds uniformly on $T^{*}\times S$ and g(t,x) is $\Delta_{n_{0}}^{\delta }$-almost periodic in t uniformly for x∈D with the ASS-function $S_{g}^{n_{0}}=g\left(t,x\right){\left(\delta_{e_{\Pi^{*}}}^{\Delta }\left(t\right)\right)}^{n_{0}}$ under the matched space (T,F,Π,δ).*

Next, we give a sequentially compact criterion of $\Delta_{n_{0}}^{\delta }$-almost periodic functions through shift operator $T^{\delta ,n_{0}}$.

**Theorem** **41** ([38])**.** *Let $f\left(t,x\right)\in C\left(T\times D,E^{n}\right)$. If for any sequence $\alpha^{{}^{\prime }}\subset \tilde{\Pi }$, there exists $\alpha \subset \alpha^{{}^{\prime }}$ such that $T_{\alpha }^{\delta ,n_{0}}\left(S_{f}^{n_{0}}\right)$ exists uniformly on $T^{*}\times S$, then f(t,x) is $\Delta_{n_{0}}^{\delta }$-almost periodic in t uniformly for x∈D with the ASS-function $S_{f}^{n_{0}}$ under the matched space (T,F,Π,δ), where $S_{f}^{n_{0}}=f\left(t,x\right){\left(\delta_{e_{\Pi^{*}}}^{\Delta }\left(t\right)\right)}^{n_{0}}$.*

From Theorems 40 and 41, we can obtain the following equivalent definition of uniformly $\Delta_{n_{0}}^{\delta }$-almost periodic functions.

**Definition** **46** ([38])**.** *Let $f\left(t,x\right)\in C\left(T\times D,E^{n}\right)$. If for any given sequence $\alpha^{{}^{\prime }}\subset \tilde{\Pi },$ there exists a subsequence $\alpha \subset \alpha^{{}^{\prime }}$ such that $T_{\alpha }^{\delta ,n_{0}}\left(S_{f}^{n_{0}}\right)$ exists uniformly on $T^{*}\times S,$ where $S_{f}^{n_{0}}=f\left(t,x\right){\left(\delta_{e_{\Pi^{*}}}^{\Delta }\left(t\right)\right)}^{n_{0}}$, then f(t,x) is called an $\Delta_{n_{0}}^{\delta }$-almost periodic function in t uniformly for x∈D with the ASS-function $S_{f}^{n_{0}}$ under the matched space (T,F,Π,δ).*

**Theorem** **42** ([38])**.** *If $f\left(t,x\right)\in C\left(T\times D,E^{n}\right)$ is $\Delta_{n_{0}}^{\delta }$-almost periodic in t uniformly for x∈D with the ASS-function $S_{f}^{n_{0}}=f\left(t,x\right){\left(\delta_{e_{\Pi^{*}}}^{\Delta }\left(t\right)\right)}^{n_{0}}$, φ(t) is $\Delta_{n_{0}}^{\delta }$-almost periodic with the ASS-function $S_{\varphi }^{n_{0}}=\varphi \left(t\right){\left(\delta_{e_{\Pi^{*}}}^{\Delta }\left(t\right)\right)}^{n_{0}}$ and $\{S_{\varphi }^{n_{0}}:t\in T\}\subset S$, then $f\left(t,S_{\varphi }^{n_{0}}\left(t\right)\right)$ is $\Delta_{n_{0}}^{\delta }$-almost periodic with the ASS-function*

$$S_{f}^{n_{0}}=f\left(t,S_{\varphi }^{n_{0}}\left(t\right)\right){\left(\delta_{e_{\Pi^{*}}}^{\Delta }\left(t\right)\right)}^{n_{0}}.$$

**Definition** **47** ([38])**.** *Let $f\left(t,x\right)\in C\left(T\times D,E^{n}\right)$. Then, $H_{n_{0}}\left(S_{f}^{n_{0}}\right)=\left\{S_{g}^{n_{0}}\left(t,x\right):T\to E^{n}\right|$ there exists $\alpha \in \tilde{\Pi }$ such that $T_{\alpha }^{\delta ,n_{0}}S_{f}^{n_{0}}\left(t,x\right)=S_{g}^{n_{0}}\left(t,x\right)$ exists uniformly on $T^{*}\times S$} is called the $n_{0}$-order hull of $S_{f}^{n_{0}}\left(t,x\right)$ under the matched space (T,F,Π,δ).*

**Theorem** **43** ([38])**.** *$H_{n_{0}}\left(S_{f}^{n_{0}}\right)$ is compact if and only if f(t,x) is $\Delta_{n_{0}}^{\delta }$-almost periodic in t uniformly for x∈D with the ASS-function $f\left(t,x\right){\left(\delta_{e_{\Pi^{*}}}^{\Delta }\left(t\right)\right)}^{n_{0}}$.*

**Theorem** **44** ([38])**.** *If f(t,x) is $\Delta_{n_{0}}^{\delta }$-almost periodic in t uniformly for x∈D with the ASS-function $S_{f}^{n_{0}}=f\left(t,x\right){\left(\delta_{e_{\Pi^{*}}}^{\Delta }\left(t\right)\right)}^{n_{0}}$ under the matched space (T,F,Π,δ), then, for any $S_{g}^{n_{0}}\left(t,x\right)\in H_{n_{0}}\left(S_{f}^{n_{0}}\right)$, we have $H_{n_{0}}\left(S_{f}^{n_{0}}\right)=H_{n_{0}}\left(S_{g}^{n_{0}}\right)$.*

Now, we establish a sufficient and necessary criterion for $\Delta_{n_{0}}^{\delta }$-almost periodic functions.

**Theorem** **45** ([38])**.** *A function f(t,x) is $\Delta_{n_{0}}^{\delta }$-almost periodic in t uniformly for x∈D with the ASS-function $S_{f}^{n_{0}}=f\left(t,x\right){\left(\delta_{e_{\Pi^{*}}}^{\Delta }\left(t\right)\right)}^{n_{0}}$ under the matched space (T,F,Π,δ) if and only if for every pair of sequences $\alpha^{{}^{\prime }},\beta^{{}^{\prime }}\subseteq \tilde{\Pi }$, there exist common subsequences $\alpha \subset \alpha^{{}^{\prime }},\beta \subset \beta^{{}^{\prime }}$ such that*

$$T_{\tilde{\delta }\left(\alpha ,\beta \right)}^{\delta ,n_{0}}S_{f}^{n_{0}}\left(t,x\right)=T_{\alpha }^{\delta ,n_{0}}T_{\beta }^{\delta ,n_{0}}S_{f}^{n_{0}}\left(t,x\right).$$

In [38], the linear $\Delta_{n_{0}}^{\delta }$-almost periodic dynamic equation on T was discussed:

(13) $$x^{\Delta }=S_{A}^{n_{0}}\left(t\right)x\left(t\right)+S_{f}^{n_{0}}\left(t\right)$$

and its associated homogeneous equation

(14) $$x^{\Delta }=S_{A}^{n_{0}}\left(t\right)x\left(t\right),$$

where A(t) is an $\Delta_{n_{0}}^{\delta }$-almost periodic matrix function and f(t) is an $\Delta_{n_{0}}^{\delta }$-almost periodic vector function.

**Theorem** **46** ([38])**.** *Let A(t) be an $\Delta_{n_{0}}^{\delta }$-almost periodic matrix function with the ASS-function $S_{A}^{n_{0}}$ and f(t) be an $\Delta_{n_{0}}^{\delta }$-almost periodic vector function with the ASS-function $S_{f}^{n_{0}}$. If *(14)* admits an exponential dichotomy, then *(13)* has a unique δ-almost periodic solution with the $\Delta_{n_{0}}^{\delta }$-almost periodic function x:*

$$S_{x}^{n_{0}}\left(t\right)=\int_{-\infty }^{t}S_{X}^{n_{0}}\left(t\right)PS_{X^{-1}}^{n_{0}}\left(\sigma \left(s\right)\right)S_{f}^{n_{0}}\left(s\right)\Delta s-\int_{t}^{+\infty }S_{X}^{n_{0}}\left(t\right)\left(I-P\right)S_{X^{-1}}^{n_{0}}\left(\sigma \left(s\right)\right)S_{f}^{n_{0}}\left(s\right)\Delta s,$$

*where $S_{X}^{n_{0}}\left(t\right)$ is the fundamental solution matrix of *(14)* and X(t) is the fundamental matrix solution for $x^{\Delta }\left(t\right)=A\left(t\right)x\left(t\right)$.*

As an application of Theorem 46, the following almost periodic dynamic equation with variable delays under the matched space (T,F,Π,δ) was considered:

(15) $$x^{\Delta }\left(t\right)=S_{A}^{n_{0}}\left(t\right)x\left(t\right)+\sum_{i=1}^{n}S_{f}^{n_{0}}\left(t,x\left(\delta (\tau_{i}\left(t\right),t)\right)\right),$$

where A(t) is an $\Delta_{n_{0}}^{\delta }$-almost periodic matrix function on T, $\tau_{i}\left(t\right):T^{*}\to \Pi^{*}$ is $\Delta_{n_{0}}^{\delta }$-almost periodic on T for every i=1,2,…,n, $f\in C(T\times R^{n},R^{n})$ is $\Delta_{n_{0}}^{\delta }$-almost periodic uniformly in *t* for $x\in R^{n}$.

**Theorem** **47** ([38])**.** *Suppose that the following hold:*

(*H*_1_) *$x^{\Delta }\left(t\right)=S_{A}^{n_{0}}\left(t\right)x\left(t\right)$ admits an exponential dichotomy on T with positive constants K and α.*
(*H*_2_) *There exists $M<\frac{\alpha }{2Kn}$ such that $|S_{f}\left(t,x\right)-S_{f}\left(t,y\right)|\leq M|S_{x}-S_{y}|$ for $t\in T,x,y\in R^{n}.$*

*Then, the system *(15)* has a unique δ-almost periodic solution with the $\Delta_{n_{0}}^{\delta }$-almost periodic affiliated function.*

## 3. The Uncertainty Theory on Time Scales with Shift Operators

As is known to all that all kinds of natural changes are full of uncertainty. To describe this inaccuracy in an accurate way, the stochastic theory and fuzzy theory are always applied to overcome these difficulties in physics and biological field (see [42,43,44,45]), etc.

In this section, we will present some recent main results of the stochastic and fuzzy dynamic equations on translational and non-translational time scales. Non-translational time scales are always with shift operators introduced in [46]. Some new equivalent concepts of the periodic time scales in shift operators were proposed in [47,48,49,50] to establish the theory of almost periodic and almost automorphic functions on irregular time scales.

### 3.1. The Stochastic Theory on Time Scales

The theory of stochastic dynamic equations was discussed in [51] and applied to study the existence and exponential stability of piecewise mean-square almost periodic solutions of the impulsive stochastic Nicholson’s blowflies model on time scales (see [15]).

Let (Ω,F,P) be a probability space and $L^{2}\left(R^{n}\right)$ stands for a space that consists of all $R^{n}$-valued random variables *x* with the norm

$${E∥x∥}^{2}=\int_{\Omega }{∥x∥}^{2}dP.$$

Let ω be a standard Wiener process and suppose {ω(t+h)−ω(t):h≥0} is independent of $F_{t}:=\sigma \left\{\omega \left(s\right):0\leq s\leq t\right\}$, where $F_{R}:=\left\{F_{t}:t\in R\right\}$ is a filtration on R, and with σ{·}, we mean the σ-algebra generated by {·}. We denote Δ-stochastic integral on ${\left[0,1\right]}_{T}$, by $\int_{0}^{1}f\left(t\right)\Delta \omega \left(t\right)$.

**Lemma** **2** ([51])**.** *The *Δ*-stochastic integral has the following properties:*

(*i*) *If $f_{1},f_{2}\in L^{2}\left({\left[0,1\right]}_{T}\right)$ and $c_{1},c_{2}\in R$, then*
  $$\int_{0}^{1}\left(c_{1}f_{1}\left(t\right)+c_{2}f_{2}\left(t\right)\right)\Delta \omega \left(t\right)=c_{1}\int_{0}^{1}f_{1}\left(t\right)\Delta \omega \left(t\right)+c_{2}\int_{0}^{1}f_{2}\left(t\right)\Delta \omega \left(t\right).$$
(*ii*) *If $E\left(\int_{0}^{1}{\left|f\left(t\right)\right|}^{2}\Delta t\right)<\infty$, then $E\left(\int_{0}^{1}f\left(t\right)\Delta \omega \left(t\right)\right)=0$ and the Itô-isometry holds, i.e.,*
  $$E\left({\left(\int_{0}^{1}f\left(t\right)\Delta \omega \left(t\right)\right)}^{2}\right)=E\left(\int_{0}^{1}f^{2}\left(t\right)\Delta t\right).$$

**Definition** **48** ([46])**.** *Let T be a time scale with the shift operators $\delta_{\pm }$ associated with the initial point $t_{0}\in T^{*}$. The time scale T is said to be periodic in shifts $\delta_{\pm }$ if there exists a $p\in {\left(t_{0},\infty \right)}_{T^{*}}$ such that $\left(p,t\right)\in D_{\mp }$ for all $t\in T^{*}$. Furthermore, if*

$$P:=\inf\left\{p\in {\left(t_{0},\infty \right)}_{T^{*}}:\left(p,t\right)\in D_{\mp }\;for\;all\;t\in T^{*}\right\}\neq \left\{t_{0}\right\},$$

*then P is called the period of the time scale T, where $D_{\pm }=\left\{\left(s,t\right)\in {\left[t_{0},\infty \right)}_{T}\times T^{*}:\delta \left(s,t\right)\in T^{*}\right\}$.*

Based on Definition 48, we introduce the following concept of relatively dense set under periodic time scales with shifts $\delta_{\pm }$.

**Definition** **49** ([47,48])**.** *Let T be a time scale with the shifts operators $\delta_{\pm }$ associated with the initial point $t_{0}\in T^{*}$. A subset S of R is called relatively dense under the shift $\delta_{+}$ if there exists a positive number $L\in {\left(t_{0},\infty \right)}_{T^{*}}$ such that ${\left[a,\delta_{+}\left(L,a\right)\right]}_{T^{*}}\cap S\neq \emptyset$ for all $a\in T^{*}$. The number L is called the inclusion length with respect to the pair $\left(T^{*},\delta_{+}\right)$.*

**Remark** **3.** *In fact, some classical definitions of relatively dense set from Definition 49 can be addressed below.*

(*i*) *Let $T=R,\;\delta_{+}\left(L,a\right)=a+L$. Definition 49 can be written as:*
  **Definition** **50.** *A subset S of R is called relatively dense if there exists a positive number L such that [a,a+L]∩S≠∅ for all a∈R.*
(*ii*) *Let $T=\bar{q^{Z}},\;q>1,\;\delta_{+}\left(L,a\right)=aL$, Definition 49 is equivalent to the notion of relatively dense set on quantum time scale:*
  **Definition** **51.** *A subset S of R is called relatively dense if there exists a positive number $L\in \left(1,\infty \right)\cap q^{Z}$ such that ${\left[a,aL\right]}_{q^{Z}}\cap S\neq \emptyset$ for all $a\in q^{Z}$.*
(*iii*) *Let $T=N^{\frac{1}{2}},\;\delta_{+}\left(L,a\right)=\sqrt{L^{2}+a^{2}}$. The concept of relatively dense set on this irregular time scale follows immediately:*
  **Definition** **52.** *A subset S of R is called relatively dense if there exists a positive number $L\in \left(0,\infty \right)\cap N^{\frac{1}{2}}$ such that ${\left[a,\sqrt{L^{2}+a^{2}}\right]}_{N^{\frac{1}{2}}}\cap S\neq \emptyset$ for all $a\in N^{\frac{1}{2}}$.*
(*iv*) *Let $T=Z,\;\delta_{+}\left(L,a\right)=a+L$. The concept of relatively dense set in discrete situation can be stated as follows:*
  **Definition** **53.** *A subset S of R is called relatively dense if there exists a positive number L∈(0,∞)∩Z such that ${\left[a,a+L\right]}_{Z}\cap S\neq \emptyset$ for all a∈Z.*

*From (i),(ii),(iii),(iv), it easily follows that Definition 49 is efficient and feasible to cover some important irregular time scales. Based on it, the almost periodic functions on irregular time scales can be introduced.*

For convenience, $PC_{rd}\left(T,L^{2}\left(R^{n}\right)\right)$ denotes the set of all piecewise continuous stochastic process with respect to a sequence $\{t_{k}\},\;k\in Z$.

By Lemma 1 from [46], the following lemma follows.

**Lemma** **3** ([47,48])**.** *If $t_{k}^{j}=\delta_{-}\left(t_{k},t_{k+j}\right)$ and k,j∈Z, then*

$$\delta_{-}\left(t_{k}^{j},t_{k+k_{1}}^{j}\right)=\delta_{-}\left(t_{k}^{k_{1}},t_{k+j}^{k_{1}}\right),\;\;\delta_{-}\left(t_{k}^{k_{1}},t_{k}^{j}\right)=t_{k+k_{1}}^{j-k_{1}}.$$

According to Lemma 3, we adopt the notion $t_{k}^{j}:=\delta_{-}\left(t_{k},t_{k+j}\right)$ and introduce the concept of equipotentially almost periodic sequence under the shifts operators $\delta_{\pm }$.

**Definition** **54** ([47,48])**.** *For any ε>0, let $\Gamma_{\varepsilon }\subset T^{*}$ be a set of real numbers and $\left\{t_{k}\right\}\subset T^{*}$. We say $\{t_{k}^{j}\},\;k,j\in Z$ is equipotentially almost periodic under the shifts operators $\delta_{\pm }$ if for $r\in \Gamma_{\varepsilon }$, there exists at least one integer q such that $|t_{k}^{q}-r|<\varepsilon ,\;\;for\;all\;k\in Z.$*

Based on Definition 49, we can introduce the following new concepts of almost periodic stochastic process. Let $\Omega \subset L^{2}\left(R^{n}\right)$ or $\Omega =L^{2}\left(R^{n}\right)$, we will introduce the following definitions.

Letting $t_{0}$ be the initial point and $\Pi :=\left\{p\in T^{*}:\left(p,t\right)\in D_{\pm }\;\mathrm{for}\;\mathrm{all}\;t\in T^{*}\right\}\notin \left\{\{t_{0}\},\emptyset \right\}$, then for any s∈Π, we define a function A:Π→Π,

$$A\left(s\right)=\left\{\begin{array}{c} \delta_{+}\left(s,t_{0}\right),\;\;\;\;s>t_{0},\\ \delta_{-}\left(s,t_{0}\right),\;\;\;\;s<t_{0}, \end{array}\right.$$

which will be used later. Note that $A\left(s\right)>t_{0}$ and A(s)≥s.

**Definition** **55** ([47,48])**.** *Let T be periodic in shifts $\delta_{\pm }$ and $t_{0}\in T^{*}$ be an initial point. $\left\{t_{k}\right\}\subset T^{*}$ satisfies that the derived sequence $\{t_{k}^{j}\},\;k,j\in Z$, is equipotentially almost periodic under the shifts operators $\delta_{\pm }$. We call a stochastic process $\varphi \in PC_{rd}\left(T\times \Omega ,L^{2}\left(R^{n}\right)\right)$ mean-square almost periodic in t uniformly for x∈Ω if for any ε>0 and for each compact subset S of *Ω*:*

(*i*) *there is a positive number $\delta^{*}=\delta^{*}\left(\varepsilon ,S\right)$ such that if the points $t^{{}^{\prime }}$ and $t^{{}^{\prime \prime }}$ belong to the same interval of continuity and $A\left(\delta_{-}\left(t^{{}^{\prime }},t^{{}^{\prime \prime }}\right)\right)<\delta^{*}$, then $E∥\varphi \left(t^{{}^{\prime }},x\right)-\varphi \left(t^{{}^{\prime \prime }},x\right){∥}^{2}<\varepsilon$ for all $t^{{}^{\prime }},t^{{}^{\prime \prime }}\in T^{*}$;*
(*ii*) *there is relative dense set $\Gamma_{0}\subset {\left(t_{0},\infty \right)}_{T^{*}}$ of mean-square ε-almost periods with respect to the pair $\left(T^{*},\delta_{+}\right)$ such that if $\tau \in \Gamma_{0}$, then $E∥\varphi \left(\delta_{+}\left(\tau ,t\right),x\right)-\varphi \left(t,x\right){∥}^{2}<\varepsilon$ for all $\left(t,x\right)\in T^{*}\times S$ which satisfies the condition $A\left(\delta_{-}\left(t,t_{k}\right)\right)>\varepsilon ,\;k\in Z$.*

In 2017, Wang and Agarwal firstly proposed the concept of relatively dense set under time scales with shift operators and established the following basic notions and properties to investigate the almost periodicity and almost automorphy of impulsive dynamic equations on more general hybrid time scales (see [47,49]).

Let

$$D_{\pm }=\left\{\left(s,t\right)\in T^{*}\times T^{*}:\delta_{\pm }\left(s,t\right)\in T^{*}\right\}.$$

For any $s\in T^{*}$, denote

(16) $$T_{*}^{\delta_{s^{-}}}:=\delta_{-}\left(s,T^{*}\right):=\left\{\delta_{-}\left(s,t\right):\left(s,t\right)\in D_{-},\;\forall t\in T^{*}\right\},$$

(17) $$T_{*}^{\delta_{s^{+}}}:=\delta_{+}\left(s,T^{*}\right):=\left\{\delta_{+}\left(s,t\right):\left(s,t\right)\in D_{+},\;\forall t\in T^{*}\right\}.$$

**Definition** **56** ([50])**.** *Let T be a time scale attached with the shifts operators $\delta_{\pm }$ and $t_{0}\in T^{*}$ is the initial point. The time scale T is called bi-direction* ***shift complete-closed time scales*** *(or S-CCTS for short) in shifts $\delta_{\pm }$ if*

(18) $$\Pi :=\left\{p\in T^{*}:\left(p,t\right)\in D_{\pm }\;for\;all\;t\in T^{*}\right\}\notin \left\{\{t_{0}\},\emptyset \right\}.$$

By (16) and (17), we may rewrite (18) into the equivalent form $\Pi =\left\{p\in T^{*}:\;T_{*}^{\delta_{p^{\pm }}}\subseteq T^{*}\right\}\notin \left\{\{t_{0}\},\emptyset \right\}.$

Furthermore, from (18), we will refine the following the concept of S-CCTS attached with shift direction. For convenience, we will use the notations

$$\Pi^{+}:=\left\{p\in T^{*}:\;T_{*}^{\delta_{p}}\subseteq T^{*}\right\},\;\;\;\Pi^{-}:=\left\{p\in T^{*}:\;T_{*}^{\delta_{p^{-}}}\subseteq T^{*}\right\}.$$

**Definition** **57** ([50])**.** *Let T be an S-CCTS. Then,*

(*i*) *we say S-CCTS is with positive-direction if $\Pi^{+}\notin \left\{\{t_{0}\},\emptyset \right\}$;*
(*ii*) *we say S-CCTS is with negative-direction if $\Pi^{-}\notin \left\{\{t_{0}\},\emptyset \right\}$;*
(*iii*) *we say S-CCTS is with bi-direction if $\Pi \notin \left\{\{t_{0}\},\emptyset \right\}$.*

Through Definitions 49 and 55, the authors investigated the almost periodic oscillations for delay impulsive stochastic Nicholson’s blowflies timescale model and the almost periodic dynamical behavior of a new type of neutral impulsive stochastic Lasota-Wazewska timescale model, respectively.

In [48], two new concepts of mean-square almost periodic stochastic processes were first introduced and the following timescale model was considered:

(19) $$\left\{\begin{array}{c} \Delta \left(x_{i}\left(t\right)+c_{i}\left(t\right)x_{i}\left(\delta_{-}\left(\tau_{i},t\right)\right)\right)=\left[-\alpha_{i}\left(t\right)x_{i}\left(t\right)+\sum_{j=1}^{m}\beta_{ij}\left(t\right)e^{-\gamma_{ij}\left(t\right)x_{j}\left(\delta_{-}\left(\tau_{ij},t\right)\right)}\right]\Delta t\\ \;\;\;\;\;\;\;\;\;+\sum_{j=1}^{m}H_{ij}\left(t,x_{j}\left(\delta_{-}\left(\sigma_{ij},t\right)\right)\right)\Delta \omega_{j}\left(t\right),\;\;t\neq t_{k},\\ \tilde{\Delta }x_{i}\left(t_{k}\right)=x_{i}\left(t_{k}^{+}\right)-x_{i}\left(t_{k}^{+}\right)=I_{ik}\left(x_{i}\left(t_{k}\right)\right)+\alpha_{ik}x_{i}\left(t_{k}\right)+\nu_{ik},\;\;t=t_{k}, \end{array}\right.$$

where $x_{i}$ denotes the number of the red blood cells at time *t* of the *i*th animal, $c_{i}\left(t\right)$ is the stimulative rate of the generation of red blood cells per unit time, and $\tau_{i}$ is the stimulative time needed to produce blood cells of the *i*th animal. $\alpha_{i}$ is the rate of death of the red blood cells of the *i*th animal, $\beta_{ij}$ and $\gamma_{ij}$ describe the generation of red blood cells per unit time and $\tau_{ij}$ is the time needed to produce blood cells of the *i*th animal when blood of the *j*th animal is transfused into the *i*th one. $\Delta x_{i}\left(t\right)$ denotes a Δ-stochastic differential of $x_{i}\left(t\right)$, $\alpha_{i},\beta_{ij},\gamma_{ij}\in PC_{rd}\left(T,R^{+}\right)$, $\tau_{i},\tau_{ij},\sigma_{ij}$ are some positive constants, $\{t_{k}\}\in B$, $B=\left\{\left\{t_{k}\right\}:t_{k}\in T,\;t_{k}<t_{k+1},\;k\in Z,\;\lim_{k\to \pm \infty }=\pm \infty \right\}$, the constants $\alpha_{ik},\nu_{ik}\in R$ and $I_{ik}\in C\left(L^{2}\left(R\right),R\right)$, $H_{ij}$ is Borel measurable, i=1,2,…,n,j=1,2,…,m,k∈Z and $A={\left(H_{ij}\right)}_{n\times m}$ is a diffusion coefficient matrix (i.e., the random perturbation term for the system). The operator $\delta_{\pm }:T^{*}\to T^{*}$ are shifts operators satisfying all the conditions in Definition 3 from [46] (here $\bar{T^{*}}=T$, $\bar{T^{*}}$ denotes the closure of T, i.e, $T^{*}$ is the largest subset of T). Let (Ω,F,P) be a complete probability space furnished with a complete family of right continuous increasing sub σ-algebras $\left\{F_{t}:t\in {\left[0,+\infty \right)}_{T}\right\}$ satisfying $F_{t}\subset F$. $\omega \left(t\right)=\left(\omega_{1}\left(t\right),\omega_{2}\left(t\right),\ldots ,\omega_{m}\left(t\right)\right)$ is an *m*-dimensional standard Brownian motion over (Ω,F,P). Some sufficient conditions are obtained ensuring the existence of mean-square almost periodic solutions for system (19) by inverse operator theorem and fixed point theorem.

The following result concerning the existence of square-mean positive almost periodic solutions for (19) was established in [48].

**Theorem** **48** ([48])**.** *If the conditions $\left(A_{1}\right)-\left(A_{4}\right)$ are fulfilled—if $\left(A_{5}\right)$ holds, i.e, the following inequalities holds:*

$$\begin{array}{ccc} & & \frac{3K^{2}}{\lambda^{2}}\{2{\left(\frac{1}{1-c^{M}}\right)}^{2}\left[{\left(\sum_{i=1}^{n}\sum_{j=1}^{m}\left(\beta_{ij}^{M}\gamma_{ij}^{M}\right)\right)}^{2}+{\left(\sum_{i=1}^{n}\alpha_{i}^{M}c_{i}^{M}\right)}^{2}\right]\\ & & +{\left(\sum_{i=1}^{n}\sum_{j=1}^{m}\frac{l_{ij}^{\frac{1}{2}}}{1-c_{i}^{M}}\right)}^{2}\}+\frac{3K^{2}}{{\left(1-e_{⊖\lambda }^{*}\left(\theta ,0\right)\right)}^{2}}{\left(\sum_{i=1}^{n}\frac{L_{i}^{\frac{1}{2}}\left(1+c_{i}^{M}\right)}{1-c_{i}^{M}}\right)}^{2}<1,\;\;\mathrm{then} \end{array}$$

*there exists a unique piecewise mean-square almost periodic solution x(t) of system *(19)* in the region $B^{*}=\left\{\tilde{\varphi }:\tilde{\varphi }\in PC_{rd}\left(T,L^{2}\left(R^{n}\right)\right),E{∥\tilde{\varphi }\left(t\right)∥}^{2}\leq {\left(\frac{K_{0}}{1-c^{M}}\right)}^{2},t\in T\right\}$.*

### 3.2. The Fuzzy Theory on Time Scales

Time scale theory is also a powerful tool in establishing the fuzzy theory on hybrid domains. Based on the Hilger theory, in [50], Wang, Agarwal, and O’Regan established the theory of calculus of fuzzy vector-valued functions and almost periodic fuzzy vector-valued functions on time scales.

**Definition** **58** ([52,53])**.** *Letting $K_{C}^{n}$ be the space of nonempty compact convex set of $R^{n}$, $A,B\in K_{C}^{n}$, we define the generalized Hukuhara difference of A and B as the set $C\in K_{C}^{n}$ such that*

(20) $$A{⊟}_{gH}B=C\Leftrightarrow \left\{\begin{array}{c} (I)\;\;A=B+C\;or\\ (II)\;\;B=A+(-1)\cdot C. \end{array}\right.$$

In the following part, we establish an embedding theorem for fuzzy multidimensional space.

**Definition** **59** ([50])**.** *Let $u_{i}\in R_{F}$ for each i=1,2,…,n. We say $u=\left(u_{1},u_{2},\ldots ,u_{n}\right)\in \underset{n\;terms}{\underset{︸}{R_{F}\times R_{F}\times \ldots \times R_{F}}}=\times_{i=1}^{n}\left\{R_{F}\right\}:=\left[R_{F}^{n}\right]$ is a fuzzy (box) vector, where $\times_{i=1}^{n}$ denotes the Cartesian product.*

**Remark** **4.** *Let $u=\left(u_{1},u_{2},\ldots ,u_{n}\right)\in \left[R_{F}^{n}\right]$, then the α-level of u are multidimensional intervals (box) of $R^{n}$(see Section 3 from Stefanini [53]). In fact, a multidimensional interval (box) of $R^{n}$ can be regarded as a fuzzy (box) vector.*

Let $u=(u_{1},u_{2},\ldots ,u_{n})$ and $v=(v_{1},v_{2},\ldots ,v_{n})$ be two fuzzy vectors with (box) α-levels:

$${\left[u\right]}^{\alpha }=\left[u_{1,\alpha }^{-},u_{1,\alpha }^{+}\right]\times \left[u_{2,\alpha }^{-},u_{2,\alpha }^{+}\right]\times \ldots \times \left[u_{n,\alpha }^{-},u_{n,\alpha }^{+}\right]:=\times_{i=1}^{n}\left[u_{i,\alpha }^{-},u_{i,\alpha }^{+}\right],$$

$${\left[v\right]}^{\alpha }=\left[v_{1,\alpha }^{-},v_{1,\alpha }^{+}\right]\times \left[v_{2,\alpha }^{-},v_{2,\alpha }^{+}\right]\times \ldots \times \left[v_{n,\alpha }^{-},v_{n,\alpha }^{+}\right]:=\times_{i=1}^{n}\left[v_{i,\alpha }^{-},v_{i,\alpha }^{+}\right].$$

The distance is defined by

(21) $$\begin{array}{ccc} D_{\infty }\left(u,v\right) & = & \underset{\alpha \in [0,1]}{\sup}\max\{{\left[\sum_{i=1}^{n}{\left|s_{u}\left(\alpha ,P_{i}\right)-s_{v}\left(\alpha ,P_{i}\right)\right|}^{2}\right]}^{\frac{1}{2}},{\left[\sum_{i=1}^{n}{\left|s_{u}\left(\alpha ,P_{i}^{*}\right)-s_{v}\left(\alpha ,P_{i}^{*}\right)\right|}^{2}\right]}^{\frac{1}{2}}\\ & & :\alpha \in \left[0,1\right],\;\;P_{i},P_{i}^{*}\in S^{n-1}\cap V^{n-1},\;i=1,2,\ldots ,n\}, \end{array}$$

and the distance $D_{\infty }\left(\cdot ,\cdot \right)$ induces ${∥\cdot ∥}_{F}$ on $\left[R_{F}^{n}\right]$ defined by ${∥u∥}_{F}=D_{\infty }\left(u,\tilde{0}\right)$, where $\tilde{0}=\left(\tilde{0},\tilde{0},\ldots ,\tilde{0}\right)$ and $\tilde{0}$ is a zero element of $R_{F}$. In fact, because

$$\left[-s_{u}\left(\alpha ,P_{i}^{*}\right),s_{u}\left(\alpha ,P_{i}\right)\right]=\left[u_{i,\alpha }^{-},u_{i,\alpha }^{+}\right],\;\;i=1,2,\ldots ,n,$$

$$\left[-s_{v}\left(\alpha ,P_{i}^{*}\right),s_{v}\left(\alpha ,P_{i}\right)\right]=\left[v_{i,\alpha }^{-},v_{i,\alpha }^{+}\right],\;\;i=1,2,\ldots ,n,$$

then

$${\left[u{\tilde{-}}_{gH}v\right]}^{\alpha }={\left[u\right]}^{\alpha }{⊟}_{gH}{\left[v\right]}^{\alpha }=\left\{\begin{array}{c} \left(i\right)\;\times_{i=1}^{n}\left[s_{v}\left(\alpha ,P_{i}^{*}\right)-s_{u}\left(\alpha ,P_{i}^{*}\right),s_{u}\left(\alpha ,P_{i}\right)-s_{v}\left(\alpha ,P_{i}\right)\right]\;\;\mathrm{or}\\ \left(ii\right)\;\times_{i=1}^{n}\left[s_{u}\left(\alpha ,P_{i}\right)-s_{v}\left(\alpha ,P_{i}\right),s_{v}\left(\alpha ,P_{i}^{*}\right)-s_{u}\left(\alpha ,P_{i}^{*}\right)\right], \end{array}\right.$$

so, from (21), we have

$$\begin{array}{ccc} D_{\infty }\left(u,v\right) & = & \underset{\alpha \in [0,1]}{\sup}{\{∥\left[u\right]}^{\alpha }{⊟}_{gH}{\left[v\right]}^{\alpha }{∥}_{*}\}=∥u{\tilde{-}}_{gH}{v∥}_{F}\\ & = & \underset{\alpha \in [0,1]}{\sup}\max\{{\left[\sum_{i=1}^{n}{\left|s_{u}\left(\alpha ,P_{i}\right)-s_{v}\left(\alpha ,P_{i}\right)\right|}^{2}\right]}^{\frac{1}{2}},{\left[\sum_{i=1}^{n}{\left|s_{u}\left(\alpha ,P_{i}^{*}\right)-s_{v}\left(\alpha ,P_{i}^{*}\right)\right|}^{2}\right]}^{\frac{1}{2}}\\ & & :\alpha \in \left[0,1\right],\;\;P_{i},P_{i}^{*}\in S^{n-1}\cap V^{n-1},\;i=1,2,\ldots ,n\}. \end{array}$$

**Remark** **5.** *For each i=1,2,…,n, if we introduce the distance*

$$\begin{array}{ccc} D_{\infty }^{\left(i\right)}\left(u_{i},v_{i}\right) & = & \underset{\alpha \in [0,1]}{\sup}\max\{|s_{u}\left(\alpha ,P_{i}\right)-s_{v}\left(\alpha ,P_{i}\right)\left|,\right|s_{u}\left(\alpha ,P_{i}^{*}\right)-s_{v}\left(\alpha ,P_{i}^{*}\right)|:\\ & & \alpha \in \left[0,1\right],\;\;P_{i},P_{i}^{*}\in S^{n-1}\cap V^{n-1}\}, \end{array}$$

*the distance $D_{\infty }^{\left(i\right)}\left(\cdot ,\cdot \right)$ induces ${∥\cdot ∥}_{F_{0}}$ on $R_{F}$ defined by $∥u_{i}{∥}_{F_{0}}=D_{\infty }\left(u_{i},\tilde{0}\right)$, and then it follows that*

$$D_{\infty }\left(u,v\right)={∥u{\tilde{-}}_{gH}v∥}_{F}={\left(\sum_{i=1}^{n}D_{\infty }^{\left(i\right)}\left(u_{i},v_{i}\right)\right)}^{\frac{1}{2}}={\left(\sum_{i=1}^{n}{∥u_{i}-v_{i}∥}_{F_{0}}^{2}\right)}^{\frac{1}{2}}.$$

**Theorem** **49** ([50])**.** *The metric space $\left(\left[R_{F}^{n}\right],D_{\infty }\right)$ is complete.*

In addition, the following theorem can be proved immediately.

**Theorem** **50** ([50])**.** *$\times_{i=1}^{n}\left(\bar{C}\left[0,1\right]\times \bar{C}\left[0,1\right]\right)$, with the norm defined by*

$$∥\left(\left(f_{1},g_{1}\right),\left(f_{2},g_{2}\right),\ldots ,\left(f_{n},g_{n}\right)\right){∥}_{\times_{i=1}^{n}\left(\bar{C}\times \bar{C}\right)}=\underset{x\in [0,1]}{\sup}\max\left\{{\left(\sum_{i=1}^{n}f_{i}^{2}\left(x\right)\right)}^{\frac{1}{2}},{\left(\sum_{i=1}^{n}g_{i}^{2}\left(x\right)\right)}^{\frac{1}{2}}\right\}$$

*is a Banach space.*

The embedding theorem was established as follows.

**Theorem** **51** (Embedding theorem of fuzzy multidimensional space, [50])**.** *For all $u\in [R_{F}^{n}]$, denote $j\left(u\right)=\times_{i=1}^{n}\left(u_{i}^{-},u_{i}^{+}\right)$. Then, $j(\left[R_{F}^{n}\right])$ is a closed convex cone with vertex 0 in $\times_{i=1}^{n}\left(\bar{C}\left[0,1\right]\times \bar{C}\left[0,1\right]\right)$ and $j:\left[R_{F}^{n}\right]\to \times_{i=1}^{n}\left(\bar{C}\left[0,1\right]\times \bar{C}\left[0,1\right]\right)$ satisfies:*

(*i*) *for all $u,v\in [R_{F}^{n}]$, $\hat{s},t\geq 0$, $j\left(\hat{s}\cdot u\tilde{+}t\cdot v\right)=\hat{s}j\left(u\right)+tj\left(v\right)$;*
(*ii*) *$D_{\infty }\left(u,v\right)={∥j\left(u\right)-j\left(v\right)∥}_{\times_{i=1}^{n}\left(\bar{C}\times \bar{C}\right)}$;*

*i.e., j embeds $\left[R_{F}^{n}\right]$ into $\times_{i=1}^{n}\left(\bar{C}\left[0,1\right]\times \bar{C}\left[0,1\right]\right)$ isometrically and isomorphically.*

Next, six new types of multiplication of two compact intervals were introduced as follows.

Let $\left[u^{-},u^{+}\right]$ and $\left[v^{-},v^{+}\right]$ be two compact intervals and ab denote the ordinary product of real numbers a,b. For convenience, we introduce the following notations:

$$I_{u,v}^{\left(I\right)}=\left|\begin{array}{cc} u^{-} & u^{+}\\ v^{-} & v^{+} \end{array}\right|,\;I_{u,v}^{\left(II\right)}=\left|\begin{array}{cc} u^{+} & u^{-}\\ v^{-} & v^{+} \end{array}\right|,\;I_{u,v}^{\left(III\right)}=\left|\begin{array}{cc} u^{-} & u^{-}\\ v^{-} & v^{+} \end{array}\right|,$$

$$I_{u,v}^{\left(IV\right)}=\left|\begin{array}{cc} u^{+} & u^{+}\\ v^{-} & v^{+} \end{array}\right|,\;I_{u,v}^{\left(V\right)}=\left|\begin{array}{cc} u^{-} & u^{+}\\ v^{-} & v^{-} \end{array}\right|,\;I_{u,v}^{\left(VI\right)}=\left|\begin{array}{cc} u^{-} & u^{+}\\ v^{+} & v^{+} \end{array}\right|.$$

For any $\left[a^{-},a^{+}\right]\subseteq \left[u^{-},u^{+}\right]$ and $\left[b^{-},b^{+}\right]\subseteq \left[v^{-},v^{+}\right]$, we defined the following multiplications:

(22) $$\mathrm{Type}\;I.\;\;\left[a^{-},a^{+}\right]\circ \left[b^{-},b^{+}\right]=\left\{a⋇b:a\in \left[a^{-},a^{+}\right],\;b\in \left[b^{-},b^{+}\right]\right\},$$

where if $I_{u,v}^{\left(I\right)}\leq 0$, then

$$a⋇b=\left\{\begin{array}{c} ab,\;\;\;\;\;\;\;ab\in [u^{-}v^{+},u^{+}v^{-}],\\ u^{-}v^{+},\;\;\;ab<u^{-}v^{+},\\ u^{+}v^{-},\;\;\;ab>u^{+}v^{-}; \end{array}\right.$$

if $I_{u,v}^{\left(I\right)}\geq 0$, then

$$a⋇b=\left\{\begin{array}{c} ab,\;\;\;\;\;\;\;ab\in [u^{+}v^{-},u^{-}v^{+}],\\ u^{+}v^{-},\;\;\;ab<u^{+}v^{-},\\ u^{-}v^{+},\;\;\;ab>u^{-}v^{+}. \end{array}\right.$$

(23) $$\mathrm{Type}\;\mathrm{II}.\;\;\left[a^{-},a^{+}\right]⊚\left[b^{-},b^{+}\right]=\left\{a⋇b:a\in \left[a^{-},a^{+}\right],\;b\in \left[b^{-},b^{+}\right]\right\},$$

where if $I_{u,v}^{\left(II\right)}\leq 0$, then

$$a⋇b=\left\{\begin{array}{c} ab,\;\;\;\;\;\;\;ab\in [u^{+}v^{+},u^{-}v^{-}],\\ u^{+}v^{+},\;\;\;ab<u^{+}v^{+},\\ u^{-}v^{-},\;\;\;ab>u^{-}v^{-}; \end{array}\right.$$

if $I_{u,v}^{\left(II\right)}\geq 0$, then

$$a⋇b=\left\{\begin{array}{c} ab,\;\;\;\;\;\;\;ab\in [u^{-}v^{-},u^{+}v^{+}],\\ u^{-}v^{-},\;\;\;ab<u^{-}v^{-},\\ u^{+}v^{+},\;\;\;ab>u^{+}v^{+}. \end{array}\right.$$

(24) $$\mathrm{Type}\;\mathrm{III}.\;\;\left[a^{-},a^{+}\right]⊠\left[b^{-},b^{+}\right]=\left\{a⋇b:a\in \left[a^{-},a^{+}\right],\;b\in \left[b^{-},b^{+}\right]\right\},$$

where if $I_{u,v}^{\left(III\right)}\leq 0$, then

$$a⋇b=\left\{\begin{array}{c} ab,\;\;\;\;\;\;\;ab\in [u^{-}v^{+},u^{-}v^{-}],\\ u^{-}v^{+},\;\;\;ab<u^{-}v^{+},\\ u^{-}v^{-},\;\;\;ab>u^{-}v^{-}; \end{array}\right.$$

if $I_{u,v}^{\left(III\right)}\geq 0$, then

$$a⋇b=\left\{\begin{array}{c} ab,\;\;\;\;\;\;\;ab\in [u^{-}v^{-},u^{-}v^{+}],\\ u^{-}v^{-},\;\;\;ab<u^{-}v^{-},\\ u^{-}v^{+},\;\;\;ab>u^{-}v^{+}. \end{array}\right.$$

(25) $$\mathrm{Type}\;\mathrm{IV}.\;\;\left[a^{-},a^{+}\right]⊡\left[b^{-},b^{+}\right]=\left\{a⋇b:a\in \left[a^{-},a^{+}\right],\;b\in \left[b^{-},b^{+}\right]\right\},$$

where if $I_{u,v}^{\left(IV\right)}\leq 0$, then

$$a⋇b=\left\{\begin{array}{c} ab,\;\;\;\;\;\;\;ab\in [u^{+}v^{-},u^{+}v^{+}],\\ u^{+}v^{-},\;\;\;ab<u^{+}v^{-},\\ u^{+}v^{+},\;\;\;ab>u^{+}v^{+}; \end{array}\right.$$

if $I_{u,v}^{\left(IV\right)}\geq 0$, then

$$a⋇b=\left\{\begin{array}{c} ab,\;\;\;\;\;\;\;ab\in [u^{+}v^{+},u^{+}v^{-}],\\ u^{+}v^{+},\;\;\;ab<u^{+}v^{+},\\ u^{+}v^{-},\;\;\;ab>u^{+}v^{-}. \end{array}\right.$$

(26) $$\mathrm{Type}\;V.\;\;\left[a^{-},a^{+}\right]⊗\left[b^{-},b^{+}\right]=\left\{a⋇b:a\in \left[a^{-},a^{+}\right],\;b\in \left[b^{-},b^{+}\right]\right\},$$

where if $I_{u,v}^{\left(V\right)}\leq 0$, then

$$a⋇b=\left\{\begin{array}{c} ab,\;\;\;\;\;\;\;ab\in [u^{-}v^{-},u^{+}v^{-}],\\ u^{-}v^{-},\;\;\;ab<u^{-}v^{-},\\ u^{+}v^{-},\;\;\;ab>u^{+}v^{-}; \end{array}\right.$$

if $I_{u,v}^{\left(V\right)}\geq 0$, then

$$a⋇b=\left\{\begin{array}{c} ab,\;\;\;\;\;\;\;ab\in [u^{+}v^{-},u^{-}v^{-}],\\ u^{+}v^{-},\;\;\;ab<u^{+}v^{-},\\ u^{-}v^{-},\;\;\;ab>u^{-}v^{-}. \end{array}\right.$$

(27) $$\mathrm{Type}\;\mathrm{VI}.\;\;\left[a^{-},a^{+}\right]⊙\left[b^{-},b^{+}\right]=\left\{a⋇b:a\in \left[a^{-},a^{+}\right],\;b\in \left[b^{-},b^{+}\right]\right\},$$

where if $I_{u,v}^{\left(VI\right)}\leq 0$, then

$$a⋇b=\left\{\begin{array}{c} ab,\;\;\;\;\;\;\;ab\in [u^{-}v^{+},u^{+}v^{+}],\\ u^{-}v^{+},\;\;\;ab<u^{-}v^{+},\\ u^{+}v^{+},\;\;\;ab>u^{+}v^{+}; \end{array}\right.$$

if $I_{u,v}^{\left(VI\right)}\geq 0$, then

$$a⋇b=\left\{\begin{array}{c} ab,\;\;\;\;\;\;\;ab\in [u^{+}v^{+},u^{-}v^{+}],\\ u^{+}v^{+},\;\;\;ab<u^{+}v^{+},\\ u^{-}v^{+},\;\;\;ab>u^{-}v^{+}. \end{array}\right.$$

Now, six types of the multiplication of fuzzy vectors induced by the multiplications of compact intervals can be defined by (22)–(27). For any α∈[0,1] and i=1,2,…,n, we introduce the notations:

$$I_{u_{i},v_{i}}^{\alpha ,(I)}=\left|\begin{array}{cc} u_{i,\alpha }^{-} & u_{i,\alpha }^{+}\\ v_{i,\alpha }^{-} & v_{i,\alpha }^{+} \end{array}\right|,\;I_{u_{i},v_{i}}^{\alpha ,(II)}=\left|\begin{array}{cc} u_{i,\alpha }^{+} & u_{i,\alpha }^{-}\\ v_{i,\alpha }^{-} & v_{i,\alpha }^{+} \end{array}\right|,\;I_{u_{i},v_{i}}^{\alpha ,(III)}=\left|\begin{array}{cc} u_{i,\alpha }^{-} & u_{i,\alpha }^{-}\\ v_{i,\alpha }^{-} & v_{i,\alpha }^{+} \end{array}\right|,$$

$$I_{u_{i},v_{i}}^{\alpha ,(IV)}=\left|\begin{array}{cc} u_{i,\alpha }^{+} & u_{i,\alpha }^{+}\\ v_{i,\alpha }^{-} & v_{i,\alpha }^{+} \end{array}\right|,\;I_{u_{i},v_{i}}^{\alpha ,(V)}=\left|\begin{array}{cc} u_{i,\alpha }^{-} & u_{i,\alpha }^{+}\\ v_{i,\alpha }^{-} & v_{i,\alpha }^{-} \end{array}\right|,\;I_{u_{i},v_{i}}^{\alpha ,(VI)}=\left|\begin{array}{cc} u_{i,\alpha }^{-} & u_{i,\alpha }^{+}\\ v_{i,\alpha }^{+} & v_{i,\alpha }^{+} \end{array}\right|,$$

then we define the following types I−VI with the (compact box) α-level set:

$$\mathrm{Type}\;I.\;{\left[u*v\right]}^{\alpha }=\times_{i=1}^{n}\left(\left[u_{i,\alpha }^{-},u_{i,\alpha }^{+}\right]\circ \left[v_{i,\alpha }^{-},v_{i,\alpha }^{+}\right]\right),$$

(28) $$\mathrm{where}\;\left[u_{i,\alpha }^{-},u_{i,\alpha }^{+}\right]\circ \left[v_{i,\alpha }^{-},v_{i,\alpha }^{+}\right]=\left\{\begin{array}{c} {}[u_{i,\alpha }^{-}v_{i,\alpha }^{+},u_{i,\alpha }^{+}v_{i,\alpha }^{-}]\;\;\mathrm{if}\;I_{u_{i},v_{i}}^{\alpha ,(I)}\leq 0,\\ {}[u_{i,\alpha }^{+}v_{i,\alpha }^{-},u_{i,\alpha }^{-}v_{i,\alpha }^{+}]\;\;\mathrm{if}\;I_{u_{i},v_{i}}^{\alpha ,(I)}\geq 0; \end{array}\right.$$

$$\mathrm{Type}\;\mathrm{II}.\;{\left[u⊛v\right]}^{\alpha }=\times_{i=1}^{n}\left(\left[u_{i,\alpha }^{-},u_{i,\alpha }^{+}\right]⊚\left[v_{i,\alpha }^{-},v_{i,\alpha }^{+}\right]\right),$$

(29) $$\mathrm{where}\;\left[u_{i,\alpha }^{-},u_{i,\alpha }^{+}\right]⊚\left[v_{i,\alpha }^{-},v_{i,\alpha }^{+}\right]=\left\{\begin{array}{c} {}[u_{i,\alpha }^{+}v_{i,\alpha }^{+},u_{i,\alpha }^{-}v_{i,\alpha }^{-}]\;\;\mathrm{if}\;I_{u_{i},v_{i}}^{\alpha ,(II)}\leq 0,\\ {}[u_{i,\alpha }^{-}v_{i,\alpha }^{-},u_{i,\alpha }^{+}v_{i,\alpha }^{+}]\;\;\mathrm{if}\;I_{u_{i},v_{i}}^{\alpha ,(II)}\geq 0; \end{array}\right.$$

$$\mathrm{Type}\;\mathrm{III}.\;{\left[u\hat{*}v\right]}^{\alpha }=\times_{i=1}^{n}\left(\left[u_{i,\alpha }^{-},u_{i,\alpha }^{+}\right]⊠\left[v_{i,\alpha }^{-},v_{i,\alpha }^{+}\right]\right),$$

(30) $$\mathrm{where}\;\left[u_{i,\alpha }^{-},u_{i,\alpha }^{+}\right]⊠\left[v_{i,\alpha }^{-},v_{i,\alpha }^{+}\right]=\left\{\begin{array}{c} {}[u_{i,\alpha }^{-}v_{i,\alpha }^{+},u_{i,\alpha }^{-}v_{i,\alpha }^{-}]\;\;\mathrm{if}\;I_{u_{i},v_{i}}^{\alpha ,(III)}\leq 0,\\ {}[u_{i,\alpha }^{-}v_{i,\alpha }^{-},u_{i,\alpha }^{-}v_{i,\alpha }^{+}]\;\;\mathrm{if}\;I_{u_{i},v_{i}}^{\alpha ,(III)}\geq 0; \end{array}\right.$$

$$\mathrm{Type}\;\mathrm{IV}.\;{\left[u\hat{⊛}v\right]}^{\alpha }=\times_{i=1}^{n}\left(\left[u_{i,\alpha }^{-},u_{i,\alpha }^{+}\right]⊡\left[v_{i,\alpha }^{-},v_{i,\alpha }^{+}\right]\right),$$

(31) $$\mathrm{where}\;\left[u_{i,\alpha }^{-},u_{i,\alpha }^{+}\right]⊡\left[v_{i,\alpha }^{-},v_{i,\alpha }^{+}\right]=\left\{\begin{array}{c} {}[u_{i,\alpha }^{+}v_{i,\alpha }^{-},u_{i,\alpha }^{+}v_{i,\alpha }^{+}]\;\;\mathrm{if}\;I_{u_{i},v_{i}}^{\alpha ,(IV)}\leq 0,\\ {}[u_{i,\alpha }^{+}v_{i,\alpha }^{+},u_{i,\alpha }^{+}v_{i,\alpha }^{-}]\;\;\mathrm{if}\;I_{u_{i},v_{i}}^{\alpha ,(IV)}\geq 0; \end{array}\right.$$

$$\mathrm{Type}\;V.\;{\left[u\tilde{*}v\right]}^{\alpha }=\times_{i=1}^{n}\left(\left[u_{i,\alpha }^{-},u_{i,\alpha }^{+}\right]⊗\left[v_{i,\alpha }^{-},v_{i,\alpha }^{+}\right]\right),$$

(32) $$\mathrm{where}\;\left[u_{i,\alpha }^{-},u_{i,\alpha }^{+}\right]⊗\left[v_{i,\alpha }^{-},v_{i,\alpha }^{+}\right]=\left\{\begin{array}{c} {}[u_{i,\alpha }^{-}v_{i,\alpha }^{-},u_{i,\alpha }^{+}v_{i,\alpha }^{-}]\;\;\mathrm{if}\;I_{u_{i},v_{i}}^{\alpha ,(V)}\leq 0,\\ {}[u_{i,\alpha }^{+}v_{i,\alpha }^{-},u_{i,\alpha }^{-}v_{i,\alpha }^{-}]\;\;\mathrm{if}\;I_{u_{i},v_{i}}^{\alpha ,(V)}\geq 0; \end{array}\right.$$

$$\mathrm{Type}\;\mathrm{VI}.\;{\left[u\tilde{⊛}v\right]}^{\alpha }=\times_{i=1}^{n}\left(\left[u_{i,\alpha }^{-},u_{i,\alpha }^{+}\right]⊙\left[v_{i,\alpha }^{-},v_{i,\alpha }^{+}\right]\right),$$

(33) $$\mathrm{where}\;\left[u_{i,\alpha }^{-},u_{i,\alpha }^{+}\right]⊙\left[v_{i,\alpha }^{-},v_{i,\alpha }^{+}\right]=\left\{\begin{array}{c} {}[u_{i,\alpha }^{-}v_{i,\alpha }^{+},u_{i,\alpha }^{+}v_{i,\alpha }^{+}]\;\;\mathrm{if}\;I_{u_{i},v_{i}}^{\alpha ,(VI)}\leq 0,\\ {}[u_{i,\alpha }^{+}v_{i,\alpha }^{+},u_{i,\alpha }^{-}v_{i,\alpha }^{+}]\;\;\mathrm{if}\;I_{u_{i},v_{i}}^{\alpha ,(VI)}\geq 0. \end{array}\right.$$

From Ref. [50], the interval multiplications (22)–(27) are well defined and have a well inclusion isotonicity, and so do (28)–(33) (see Remark 2.14 from [50]).

**Remark** **6.** *For $I_{u_{i},v_{i}}^{\alpha ,(I)}=0$ for all i=1,2,…,n, from *(28)*, we have $u_{i,\alpha }^{-}v_{i,\alpha }^{+}=u_{i,\alpha }^{+}v_{i,\alpha }^{-}$, then*

$${\left[u*v\right]}^{\alpha }=\times_{i=1}^{n}\left[u_{i,\alpha }^{-},u_{i,\alpha }^{+}\right]\circ \left[v_{i,\alpha }^{-},v_{i,\alpha }^{+}\right]=\times_{i=1}^{n}\left\{u_{i,\alpha }^{-}v_{i,\alpha }^{+}\right\}=\times_{i=1}^{n}\left\{u_{i,\alpha }^{+}v_{i,\alpha }^{-}\right\}.$$

*Similarly, for $I_{u_{i},v_{i}}^{\alpha ,(II)}=0$ for all i=1,2,…,n, from *(29)*, we have*

$${\left[u⊛v\right]}^{\alpha }=\times_{i=1}^{n}\left(\left[u_{i,\alpha }^{-},u_{i,\alpha }^{+}\right]⊚\left[v_{i,\alpha }^{-},v_{i,\alpha }^{+}\right]\right)=\times_{i=1}^{n}\left\{u_{i,\alpha }^{+}v_{i,\alpha }^{+}\right\}=\times_{i=1}^{n}\left\{u_{i,\alpha }^{-}v_{i,\alpha }^{-}\right\},$$

*noticing that $\times_{i=1}^{n}\left[a_{i},a_{i}\right]=\times_{i=1}^{n}\left\{a_{i}\right\}$ for any $a_{i}\in R$. For example, given $u=\chi_{\left[-a,a\right]}$ and $v=\chi_{\left[-b,b\right]}$ in $R_{F}$, where a,b>0, it follows that ${\left[u\right]}^{\alpha }=\left[-a,a\right]$, ${\left[v\right]}^{\alpha }=\left[-b,b\right]$ for all α∈[0,1]. Note that $I_{u,v}^{\alpha ,(I)}=I_{u,v}^{\alpha ,(II)}=0$, it indicates that ${\left[u*v\right]}^{\alpha }=\left\{-ab\right\}$ and ${\left[u⊛v\right]}^{\alpha }=\left\{ab\right\}$, i.e., $u*v=\chi_{\left\{-ab\right\}}$ and $u⊛v=\chi_{\left\{ab\right\}}$. In fact, it is easy to see that, if there exists some $\hat{I}\in \left\{I,II,\ldots ,VI\right\}$ such that $I_{u_{i},v_{i}}^{\alpha ,(\hat{I})}=0$, then the corresponding product of α-levels defined by *(28)*–*(33)* is a one-point set for Type $\hat{I}$.*

**Remark** **7.** *Since the interval multiplications defined by *(22)* and *(27)* have a well inclusion isotonicity, then *(28)* and *(33)* also has well inclusion isotonicity naturally. For example, given $u=\chi_{\left[-1,0\right]}$ and $v=\chi_{\left[-1,1\right]}$, then we have $I_{u,v}^{\alpha ,(I)}<0$ for all α∈[0,1]. Therefore, u∗v is given by*

$${\left[u*v\right]}^{\alpha }=\left[u_{\alpha }^{-},u_{\alpha }^{+}\right]\circ \left[v_{\alpha }^{-},v_{\alpha }^{+}\right]=\left[u_{\alpha }^{-}v_{\alpha }^{+},u_{\alpha }^{+}v_{\alpha }^{-}\right]=\left[-1,0\right]$$

*for all α∈[0,1]. For any given $a\in \left[-1,0\right]=\left[u_{\alpha }^{-},u_{\alpha }^{+}\right]$ and $b\in \left[-1,1\right]=\left[v_{\alpha }^{-},v_{\alpha }^{+}\right]$, it implies that*

$$a⋇b=\left\{\begin{array}{c} ab,\;\;\;ab\in [-1,0],\\ -1,\;ab<-1,\\ 0,\;\;\;\;\;\;ab>0, \end{array}\right.$$

*which indicates that, for any $\left[a,b\right]\subseteq \left[u_{\alpha }^{-},u_{\alpha }^{+}\right]$, $\left[c,d\right]\subseteq \left[v_{\alpha }^{-},v_{\alpha }^{+}\right]$, we can obtain $\left[a,b\right]\circ \left[c,d\right]\subseteq \left[u_{\alpha }^{-},u_{\alpha }^{+}\right]\circ \left[v_{\alpha }^{-},v_{\alpha }^{+}\right]$.*

**Remark** **8.** *Traditionally, the multiplication of compact intervals is induced by the ordinary multiplication of real numbers, i.e, for the real compact intervals $U=[u^{-},u^{+}]$ and $V=[v^{-},v^{+}]$, the interval $C=[c^{-},c^{+}]$ defining the multiplication C=UV is given by*

$$c^{-}=\min\left\{u^{-}v^{-},u^{-}v^{+},u^{+}v^{-},u^{+}v^{+}\right\},\;\;c^{+}=\max\left\{u^{-}v^{-},u^{-}v^{+},u^{+}v^{-},u^{+}v^{+}\right\}.$$

*In fact, C=UV={ab:a∈U,b∈V}. However, note that such a multiplication of compact intervals induced by ordinary multiplication of real numbers is completely different from the multiplications of compact intervals induced by a⋇b above. In the example of Remark 7, given $-\frac{1}{2}\in \left[u_{\alpha }^{-},u_{\alpha }^{+}\right]$, $-\frac{1}{4}\in \left[v_{\alpha }^{-},v_{\alpha }^{+}\right]$, we have $ab=\left(-\frac{1}{2}\right)\left(-\frac{1}{4}\right)=\frac{1}{8}\notin \left[-1,0\right]=\left[-1,0\right]\circ \left[-1,1\right]$ but $\left(-\frac{1}{2}\right)⋇\left(-\frac{1}{4}\right)=0\in \left[-1,0\right]=\left[-1,0\right]\circ \left[-1,1\right]$.*

**Theorem** **52** ([50])**.** *If $u,v\in [R_{F}^{n}]$, then ${∥u*v∥}_{F}\leq {∥u∥}_{F}\cdot {∥v∥}_{F}$ and ${∥u⊛v∥}_{F}\leq {∥u∥}_{F}\cdot {∥v∥}_{F}$.*

From Theorem 51 and the definition embedding *j*, we can prove the following properties easily.

**Theorem** **53** ([50])**.** *For $u,v,w\in [R_{F}^{n}]$, if the gH-difference among them exist, then the following properties hold:*

(*i*) *$D_{\infty }\left(u{\tilde{\pm }}_{gH}w,v{\tilde{\pm }}_{gH}w\right)=D_{\infty }\left(u,v\right)$;*
(*ii*) *$D_{\infty }\left(u{\tilde{\pm }}_{gH}w,v{\tilde{\pm }}_{gH}e\right)\leq D_{\infty }\left(u,v\right)+D_{\infty }\left(w,e\right)$;*
(*iii*) *$D_{\infty }\left(\mu \cdot u,\mu \cdot v\right)=\left|\mu \right|D_{\infty }\left(u,v\right)$ for μ∈R;*
(*iv*) *$D_{\infty }\left(u*w,v*w\right)\leq {∥w∥}_{F}D_{\infty }\left(u,v\right)$ if $\left(u{\tilde{-}}_{gH}v\right)*\omega =u*\omega {\tilde{-}}_{gH}v*\omega$;*
  *$D_{\infty }\left(u⊛w,v⊛w\right)\leq {∥w∥}_{F}D_{\infty }\left(u,v\right)$ if $\left(u{\tilde{-}}_{gH}v\right)⊛\omega =u⊛\omega {\tilde{-}}_{gH}v⊛\omega$;*
(*v*) *$D_{\infty }\left(\mu \cdot u,\nu \cdot u\right)={|\mu -\nu |∥u∥}_{F}$ for μ,ν≥0 or μ,ν≤0.*

In this part, we will establish some basic results of calculus of fuzzy vector-valued functions on time scales.

For convenience, we introduce the following notations.

Let $f,g:T\to [R_{F}^{n}]$, where $f=(f_{1},f_{2},\ldots ,f_{n})$, $g=(g_{1},g_{2},\ldots ,g_{n})$ with the box α-level sets (0≤α<1) as follows:

$${\left[f\left(t\right)\right]}^{\alpha }=\left[f_{1,\alpha }^{-}\left(t\right),f_{1,\alpha }^{+}\left(t\right)\right]\times \left[f_{2,\alpha }^{-}\left(t\right),f_{2,\alpha }^{+}\left(t\right)\right]\times \ldots \times \left[f_{n,\alpha }^{-}\left(t\right),f_{n,\alpha }^{+}\left(t\right)\right]=\times_{i=1}^{n}\left[f_{i,\alpha }^{-}\left(t\right),f_{i,\alpha }^{+}\left(t\right)\right]$$

and

$${\left[g\left(t\right)\right]}^{\alpha }=\left[g_{1,\alpha }^{-}\left(t\right),g_{1,\alpha }^{+}\left(t\right)\right]\times \left[g_{2,\alpha }^{-}\left(t\right),g_{2,\alpha }^{+}\left(t\right)\right]\times \ldots \times \left[g_{n,\alpha }^{-}\left(t\right),g_{n,\alpha }^{+}\left(t\right)\right]=\times_{i=1}^{n}\left[g_{i,\alpha }^{-}\left(t\right),g_{i,\alpha }^{+}\left(t\right)\right].$$

The following definition of the gH-Δ-derivative of fuzzy vector-valued functions on time scales was introduced to analyze the almost periodic fuzzy dynamic equations on time scales.

**Definition** **60** ([50])**.** *For $f:T\to [R_{F}^{n}]$ and $t\in T^{\kappa }$, we define the gH-*Δ*-derivative of $f\left(t\right),\;f^{\Delta }\left(t\right)=\left(f_{1}^{\Delta },f_{2}^{\Delta },\ldots ,f_{n}^{\Delta }\right)$, to be the fuzzy vector (if it exists) with the property that for a given ε>0, there exists a neighborhood U of t (i.e., $U={\left(t-\delta ,t+\delta \right)}_{T}$ for some δ>0) such that*

$$D_{\infty }^{\left(i\right)}\left(f_{i}\left(\sigma \left(t\right)\right){\tilde{-}}_{gH}f_{i}\left(s\right),f_{i}^{\Delta }\left(t\right)\left(\sigma \left(t\right)-s\right)\right)<\varepsilon \left|\sigma \left(t\right)-s\right|,\;\;i=1,2,\ldots ,n$$

*for all s∈U. That is, the limit*

$$f_{i}^{\Delta }\left(t\right)=\lim_{s\to t}\frac{f_{i}\left(\sigma (t)\right){\tilde{-}}_{gH}f_{i}\left(s\right)}{\sigma (t)-s}$$

*exists for each i=1,2,…,n.*

The following definition is obviously equivalent to Definition 60.

**Definition** **61** ([50])**.** *For $f:T\to R_{F}^{n}$ and $t\in T^{\kappa }$, we define the gH-*Δ*-derivative of $f\left(t\right),\;f^{\Delta }\left(t\right)=\left(f_{1}^{\Delta },f_{2}^{\Delta },\ldots ,f_{n}^{\Delta }\right)$, to be the fuzzy vector (if it exists) with the property that for a given ε>0, there exists a δ>0 such that |h|<δ implies*

$$D_{\infty }^{\left(i\right)}\left(f_{i}\left(\sigma \left(t\right)\right){\tilde{-}}_{gH}f_{i}\left(t+h\right),f_{i}^{\Delta }\left(t\right)\left(\mu \left(t\right)-h\right)\right)\leq \varepsilon \left|\mu \left(t\right)-h\right|,$$

*i.e.,*

$$\lim_{h\to 0}\frac{f_{i}\left(\sigma (t)\right){\tilde{-}}_{gH}f_{i}\left(t+h\right)}{\mu (t)-h}=f_{i}^{\Delta }\left(t\right)$$

*exists for each i=1,2,…,n.*

A sufficient and necessary condition for gH-Δ-differentiability of functions is given by the following theorem.

**Theorem** **54** ([50])**.** *Let $f:T\to R_{F}^{n}$ be a function and ${\left[f\left(t\right)\right]}^{\alpha }=\times_{i=1}^{n}\left[f_{i,\alpha }^{-}\left(t\right),f_{i,\alpha }^{+}\left(t\right)\right]$, α∈[0,1]. The function f(t) is gH-*Δ*-differentiable if $f_{i,\alpha }^{-}\left(t\right)$ and $f_{i,\alpha }^{+}\left(t\right)$ are *Δ*-differentiable real-valued functions for each i=1,2,…,n. Furthermore,*

$${\left[f^{\Delta }\left(t\right)\right]}^{\alpha }=\times_{i=1}^{n}\left[\min\left\{{\left(f_{i,\alpha }^{-}\right)}^{\Delta }\left(t\right),{\left(f_{i,\alpha }^{+}\right)}^{\Delta }\left(t\right)\right\},\max\left\{{\left(f_{i,\alpha }^{-}\right)}^{\Delta }\left(t\right),{\left(f_{i,\alpha }^{+}\right)}^{\Delta }\left(t\right)\right\}\right].$$

By Theorem 54, for the definition of gH-Δ-differentiability, we distinguished two cases, corresponding to (I) and (II) of (20).

**Definition** **62** ([50])**.** *Let $f:T\to R_{F}^{n}$ be a function and ${\left[f\left(t\right)\right]}^{\alpha }=\times_{i=1}^{n}\left[f_{i,\alpha }^{-}\left(t\right),f_{i,\alpha }^{+}\left(t\right)\right]$, α∈[0,1]. Let $f_{i,\alpha }^{-}\left(t\right)$ and $f_{i,\alpha }^{+}\left(t\right)$ be *Δ*-differentiable real-valued functions at $t_{0}\in {\left(a,b\right)}_{T}$ for each i=1,2,…,n and α∈[0,1]. We say that f is (I)-gH-*Δ*-differentiable at $t_{0}\in {\left(a,b\right)}_{T}$ if $f^{\Delta_{I}}\left(t\right)=\left(f_{1}^{\Delta_{I}}\left(t\right),f_{2}^{\Delta_{I}}\left(t\right),\ldots ,f_{n}^{\Delta_{I}}\left(t\right)\right)$ with α-level set*

(34) $${\left[f^{\Delta_{I}}\left(t\right)\right]}^{\alpha }=\times_{i=1}^{n}\left[{\left(f_{i,\alpha }^{-}\right)}^{\Delta }\left(t\right),{\left(f_{i,\alpha }^{+}\right)}^{\Delta }\left(t\right)\right],$$

*and f is (II)-gH-*Δ*-differentiable at $t_{0}\in {\left(a,b\right)}_{T}$ if $f^{\Delta_{II}}\left(t\right)=\left(f_{1}^{\Delta_{II}}\left(t\right),f_{2}^{\Delta_{II}}\left(t\right),\ldots ,f_{n}^{\Delta_{II}}\left(t\right)\right)$ with α-level set*

(35) $${\left[f^{\Delta_{II}}\left(t\right)\right]}^{\alpha }=\times_{i=1}^{n}\left[{\left(f_{i,\alpha }^{+}\right)}^{\Delta }\left(t\right),{\left(f_{i,\alpha }^{-}\right)}^{\Delta }\left(t\right)\right].$$

Similar to Ref. [53], we will introduce and study the switch between the two cases (I) and (II) in Definition 62.

**Definition** **63** ([50])**.** *We say a point $t_{0}\in {\left(a,b\right)}_{T}$ is a switching point for the gH-*Δ*-differentiability of f, if, in any neighborhood U of $t_{0}$, there exists points $t_{1}<t_{0}<t_{2}$ such that*

(*i*) *(type-I switch) at $t_{1}$*(34)* holds while *(35)* does not hold and at $t_{2}$*(35)* holds while *(34)* does not hold, or*
(*ii*) *(type-II switch) at $t_{1}$*(35)* holds while *(34)* does not hold and at $t_{2}$*(34)* holds while *(35)* does not hold.*

**Theorem** **55** ([50])**.** *If $f,g:T\to R_{F}^{n}$ is gH-*Δ*-differentiable at $t\in T^{k}$, then*

(*i*) *$f\left(\sigma \left(t\right)\right)=f\left(t\right)\tilde{+}\mu \left(t\right)\cdot f^{\Delta }\left(t\right)$ or $f\left(t\right)=f\left(\sigma \left(t\right)\right)\tilde{+}\left(-1\right)\mu \left(t\right)\cdot f^{\Delta }\left(t\right)$, i.e., $f\left(\sigma (t)\right){\tilde{-}}_{gH}f\left(t\right)=\mu \left(t\right)\cdot f^{\Delta }\left(t\right)$.*
(*ii*) *Let f,g be (I)-gH-*Δ*-differentiable at $t\in {\left(a,b\right)}_{T}$ or (II)-gH-*Δ*-differentiable at $t\in {\left(a,b\right)}_{T}$, then $f\tilde{+}g:T\to R_{F}^{n}$ is gH-*Δ*-differentiable at t and*
  $${\left(f\tilde{+}g\right)}^{\Delta }=f^{\Delta }\left(t\right)\tilde{+}g^{\Delta }\left(t\right).$$
(*iii*) *For any nonnegative constant λ∈R, $\lambda \cdot f:T\to R_{F}^{n}$ is gH-*Δ*-differentiable at t with*
  $${\left(\lambda \cdot f\right)}^{\Delta }\left(t\right)=\lambda \cdot f^{\Delta }\left(t\right).$$

In the following, we examine the relations between gH-Δ-differentiability and the integral of fuzzy vector-valued functions on time scales.

**Definition** **64** ([50])**.** *The fuzzy Aumann *Δ*-integral (or *Δ*-integral for short) of $f:{\left[a,b\right]}_{T}\to R_{F}^{n}$ is defined level-wise by*

$$\begin{array}{ccc} {\left[\int_{a}^{b}f\left(t\right)\Delta t\right]}^{\alpha } & = & \int_{a}^{b}{\left[f(t)\right]}^{\alpha }\Delta t=\times_{i=1}^{n}\left[\int_{a}^{b}{\left[f_{i}\left(t\right)\right]}^{\alpha }\Delta t\right]\\ & = & \times_{i=1}^{n}\left[\int_{a}^{b}f_{i}^{-}\left(t\right)\Delta t,\int_{a}^{b}f_{i}^{+}\left(t\right)\Delta t\right],\;\;\;\alpha \in \left[0,1\right]. \end{array}$$

Some basic calculus results of fuzzy functions are established as follows.

**Theorem** **56** ([50])**.** *Let $f:{\left[a,b\right]}_{T}\to R_{F}^{n}$ be continuous with ${\left[f\left(t\right)\right]}^{\alpha }=\times_{i=1}^{n}{\left[f_{i}^{-},f_{i}^{+}\right]}_{\alpha }$. Then,*

(*i*) *the function $F\left(t\right)=\int_{a}^{t}f\left(s\right)\Delta s$ is gH-*Δ*-differentiable and $F^{\Delta }\left(t\right)=f\left(t\right)$;*
(*ii*) *the function $F\left(t\right)=\int_{t}^{b}f\left(s\right)\Delta s$ is gH-*Δ*-differentiable and $G^{\Delta }\left(t\right)=-f\left(t\right)$;*

**Theorem** **57** ([50])**.** *If $f:{\left[a,b\right]}_{T}\to R_{F}^{n}$ is *Δ*-integrable and $c\in {\left[a,b\right]}_{T}$. Then,*

$$\int_{a}^{b}f\left(t\right)\Delta t=\int_{a}^{c}f\left(t\right)\Delta t\tilde{+}\int_{c}^{b}f\left(t\right)\Delta t.$$

**Theorem** **58** ([50])**.** *Assume that function f is gH-*Δ*-differentiable with n switching points at $c_{i}$, i=1,2…,n, $a=c_{0}<c_{1}<c_{2}<\ldots <c_{n}<c_{n+1}=b$ and exactly at these points. Then,*

$$f\left(b\right){\tilde{-}}_{gH}f\left(a\right)=\sum_{i=1}^{n}\left[\int_{c_{i-1}}^{c_{i}}f^{\Delta }\left(t\right)\Delta t{\tilde{-}}_{gH}\left(-1\right)\int_{c_{i}}^{c_{i+1}}f^{\Delta }\left(t\right)\Delta t\right].$$

*In addition,*

$$\int_{a}^{b}f^{\Delta }\left(t\right)\Delta t=\sum_{i=1}^{n+1}\left(f\left(c_{i}\right){\tilde{-}}_{gH}f\left(c_{i-1}\right)\right),$$

*where summation denotes standard fuzzy addition in this statement.*

Through our multiplication, the formula of integration by parts of fuzzy functions can be derived below.

**Theorem** **59** ([50])**.** *Assume $f,g:{\left[a,b\right]}_{T}\to R_{F}^{n}$ are (I)-gH-*Δ*-differentiable and f∗g is also (I)-gH-*Δ*-differentiable. If there is no switching point in ${\left[a,b\right]}_{T}$ and $I_{f_{i},g_{i}}^{\alpha ,(I)}>0$, $I_{f_{i}^{\sigma },g_{i}^{\Delta_{I}}}^{\alpha ,(I)}>0$, $I_{f_{i}^{\Delta_{I}},g_{i}}^{\alpha ,(I)}>0$ for each i=1,2,…,n, then*

$$\int_{a}^{b}f\left(t\right)*g^{\Delta_{I}}\left(t\right)\Delta t=\left(f\left(b\right)*g\left(b\right){\tilde{-}}_{gH}f\left(a\right)*g\left(a\right)\right){\tilde{-}}_{gH_{I}}\int_{a}^{b}f^{\Delta_{I}}\left(t\right)*g\left(\sigma (t)\right)\Delta t\;\;or$$

$$\int_{a}^{b}f\left(t\right)*g^{\Delta_{I}}\left(t\right)\Delta t=\int_{a}^{b}g\left(\sigma (t)\right)*f^{\Delta_{I}}\left(t\right)\Delta t{\tilde{-}}_{gH_{II}}\left(f\left(a\right)*g\left(a\right){\tilde{-}}_{gH}f\left(b\right)*g\left(b\right)\right).$$

By adopting determinant algorithm of the multiplication of fuzzy vectors, some arithmetic properties of the gH-Δ-derivatives of the product of two fuzzy vector-valued functions on time scales were obtained. For convenience, we adopt the notation $f(\sigma \left(t\right))=f^{\sigma }\left(t\right)$ in some statement.

**Theorem** **60** ([50])**.** *Let f,g be (I)-gH-*Δ*-differentiable, then*

(*i*) *if $I_{f_{i},g_{i}}^{\alpha ,(I)}<0$, $I_{f_{i}^{\sigma },g_{i}^{\Delta_{I}}}^{\alpha ,(I)}<0$, $I_{f_{i}^{\Delta_{I}},g_{i}}^{\alpha ,(I)}<0$ and f∗g is (I)-gH-*Δ*-differentiable, then*
  $${\left(f*g\right)}^{\Delta_{I}}=f^{\sigma }*g^{\Delta_{I}}\tilde{+}f^{\Delta_{I}}*g.$$
(*ii*) *if $I_{f_{i},g_{i}}^{\alpha ,(I)}<0$, $I_{f_{i}^{\sigma },g_{i}^{\Delta_{I}}}^{\alpha ,(I)}>0$, $I_{f_{i}^{\Delta_{I}},g_{i}}^{\alpha ,(I)}>0$ and f∗g is (II)-gH-*Δ*-differentiable, then*
  $${\left(f*g\right)}^{\Delta_{II}}=f^{\sigma }*g^{\Delta_{I}}\tilde{+}f^{\Delta_{I}}*g.$$
(*iii*) *if $I_{f_{i},g_{i}}^{\alpha ,(II)}<0$, $I_{f_{i}^{\sigma },g_{i}^{\Delta_{I}}}^{\alpha ,(II)}<0$, $I_{f_{i}^{\Delta_{I}},g_{i}}^{\alpha ,(II)}<0$ and f⊛g is (I)-gH-*Δ*-differentiable, then*
  $${\left(f⊛g\right)}^{\Delta_{I}}=f^{\sigma }⊛g^{\Delta_{I}}\tilde{+}g⊛f^{\Delta_{I}}.$$
(*iv*) *if $I_{f_{i},g_{i}}^{\alpha ,(II)}<0$, $I_{f_{i}^{\sigma },g_{i}^{\Delta_{I}}}^{\alpha ,(II)}>0$, $I_{f_{i}^{\Delta_{I}},g_{i}}^{\alpha ,(II)}>0$ and f⊛g is (II)-gH-*Δ*-differentiable, then*
  $${\left(f⊛g\right)}^{\Delta_{II}}=f^{\sigma }⊛g^{\Delta_{I}}\tilde{+}f^{\Delta_{I}}⊛g.$$
(*v*) *if $I_{f_{i},g_{i}}^{\alpha ,(I)}>0$, $I_{f_{i}^{\sigma },g_{i}^{\Delta_{I}}}^{\alpha ,(I)}>0$, $I_{f_{i}^{\Delta_{I}},g_{i}}^{\alpha ,(I)}>0$ and f∗g is (I)-gH-*Δ*-differentiable, then*
  $${\left(f*g\right)}^{\Delta_{I}}=f^{\sigma }*g^{\Delta_{I}}\tilde{+}f^{\Delta_{I}}*g.$$
(*vi*) *if $I_{f_{i},g_{i}}^{\alpha ,(I)}>0$, $I_{f_{i}^{\sigma },g_{i}^{\Delta_{I}}}^{\alpha ,(I)}<0$, $I_{f_{i}^{\Delta_{I}},g_{i}}^{\alpha ,(I)}<0$ and f∗g is (II)-gH-*Δ*-differentiable, then*
  $${\left(f*g\right)}^{\Delta_{II}}=f^{\sigma }*g^{\Delta_{I}}\tilde{+}f^{\Delta_{I}}*g.$$
(*vii*) *if $I_{f_{i},g_{i}}^{\alpha ,(II)}>0$, $I_{f_{i}^{\sigma },g_{i}^{\Delta_{I}}}^{\alpha ,(II)}>0$, $I_{f_{i}^{\Delta_{I}},g_{i}}^{\alpha ,(II)}>0$ and f⊛g is (I)-gH-*Δ*-differentiable, then*
  $${\left(f⊛g\right)}^{\Delta_{I}}=f^{\sigma }⊛g^{\Delta_{I}}\tilde{+}g⊛f^{\Delta_{I}}.$$
(*viii*) *if $I_{f_{i},g_{i}}^{\alpha ,(II)}>0$, $I_{f_{i}^{\sigma },g_{i}^{\Delta_{I}}}^{\alpha ,(II)}<0$, $I_{f_{i}^{\Delta_{I}},g_{i}}^{\alpha ,(II)}<0$ and f⊛g is (II)-gH-*Δ*-differentiable, then*
  $${\left(f⊛g\right)}^{\Delta_{II}}=f^{\sigma }⊛g^{\Delta_{I}}\tilde{+}f^{\Delta_{I}}⊛g.$$

In Ref. [50], the authors established the calculus of fuzzy vector-valued functions to study the almost periodic fuzzy vector-valued functions on time scales.

**Definition** **65** ([50])**.** *Let T be a bi-direction S-CCTS and $f:T\times D\to R_{F}^{n}$ be continuous on T×D.*

(*i*) *A function $f\in C(T\times D,R_{F}^{n})$ is called**shift almost periodic**fuzzy vector-valued function in t∈T uniformly for x∈D with shift operators if the ε-shift number set of f*
  $$E\left\{\varepsilon ,f,S_{0}\right\}=\left\{\tau \in \Pi :D_{\infty }\left(f(\delta_{\pm }\left(\tau ,t\right),x),f\left(t,x\right)\right)<\varepsilon ,\;\;for\;all\;t\in T^{*}\;and\;x\in S_{0}\right\}$$
  *is a relatively dense set with respect to the pair $\left(\Pi ,\delta_{\pm }\right)$ for all ε>0 and for each compact subset $S_{0}$ of D; that is, for any given ε>0 and each compact subset $S_{0}$ of D, there exists a constant $l(\varepsilon ,S_{0})>0$ such that each interval of length $l(\varepsilon ,S_{0})$ contains a $\tau \left(\varepsilon ,S_{0}\right)\in E\left\{\varepsilon ,f,S_{0}\right\}$ such that*
  $$D_{\infty }\left(f\left(\delta_{\pm }\left(\tau ,t\right),x\right),f\left(t,x\right)\right)<\varepsilon ,\;for\;all\;t\in T^{*}\;and\;x\in S_{0}.$$
  *Now, τ is called the ε-shift number of f and $l(\varepsilon ,S_{0})$ is called the inclusion length of $E\{\varepsilon ,f,S_{0}\}$.*
(*ii*) *A function $f\in C(T\times D,R_{F}^{n})$ is called shift normal function if for any sequence $F_{n}:T\times D\to R_{F}^{n}$ of the form $F_{n}\left(t,x\right)=f\left(\delta_{+}\left(h_{n},t\right),x\right),\;n\in N$, where ${\left(h_{n}\right)}_{n}\subset \Pi$ is a sequence of real numbers, one can extract a subsequence of ${\left(F_{n}\right)}_{n}$, converging uniformly on T×D(i.e., $\forall {\left(h_{n}\right)}_{n}\subset \Pi$, $\exists {\left(h_{n}\right)}_{k}$, $\exists F:T\to R_{F}^{n}$ which may depend on ${\left(h_{n}\right)}_{n}$), such that*
  $$D_{\infty }\left(F_{n_{k}}\left(t,x\right),F\left(t,x\right)\right)\to 0\;\;as\;k\to \infty$$
  *uniformly with respect to (t,x)∈T×D.*
(*iii*) *Let $\delta_{\pm }\left(s,t\right)$ be *Δ*-differentiable to its second argument. A function $f\in C(T\times D,R_{F}^{n})$ is called shift *Δ*-almost periodic fuzzy vector-valued function in t∈T uniformly for x∈D with shift operators if the ε-shift number set of f*
  $$E\left\{\varepsilon ,f,S_{0}\right\}=\left\{\tau \in \Pi :D_{\infty }\left(f\left(\delta_{\pm }\left(\tau ,t\right),x\right)\delta_{\pm }^{\Delta }\left(\tau ,t\right),f\left(t,x\right)\right)<\varepsilon ,\;\;for\;all\;t\in T^{*}\;and\;x\in S_{0}\right\}$$
  *is a relatively dense set with respect to the pair $\left(\Pi ,\delta_{\pm }\right)$ for all ε>0 and for each compact subset $S_{0}$ of D; that is, for any given ε>0 and each compact subset $S_{0}$ of D, there exists a constant $l(\varepsilon ,S_{0})>0$ such that each interval of length $l(\varepsilon ,S_{0})$ contains a $\tau \left(\varepsilon ,S_{0}\right)\in E\left\{\varepsilon ,f,S_{0}\right\}$ such that*
  $$D_{\infty }\left(f\left(\delta_{\pm }\left(\tau ,t\right),x\right)\delta_{\pm }^{\Delta }\left(\tau ,t\right),f\left(t,x\right)\right)<\varepsilon ,\;for\;all\;t\in T^{*}\;and\;x\in S_{0}.$$
  *Now, τ is called the ε-shift number of f and $l(\varepsilon ,S_{0})$ is called the inclusion length of $E\{\varepsilon ,f,S_{0}\}$.*
(*iv*) *Let $\delta_{\pm }\left(s,t\right)$ be *Δ*-differentiable to its second argument. A function $f\in C(T\times D,R_{F}^{n})$ is called shift *Δ*-normal function if for any sequence $F_{n}:T\times D\to R_{F}^{n}$ of the form $F_{n}\left(t,x\right)=f\left(\delta_{+}\left(h_{n},t\right),x\right)\delta_{+}^{\Delta }\left(h_{n},t\right),\;n\in N$, where ${\left(h_{n}\right)}_{n}\subset \Pi$ is a sequence of real numbers, one can extract a subsequence of ${\left(F_{n}\right)}_{n}$, converging uniformly on T×D(i.e., $\forall {\left(h_{n}\right)}_{n}\subset \Pi$, $\exists {\left(h_{n}\right)}_{k}$, $\exists F:T\to R_{F}^{n}$ which may depend on ${\left(h_{n}\right)}_{n}$), such that*
  $$D_{\infty }\left(F_{n_{k}}\left(t,x\right),F\left(t,x\right)\right)\to 0\;\;as\;k\to \infty$$
  *uniformly with respect to (t,x)∈T×D.*

For convenience, we denote $AP_{S}\left(T\right)$ the set of all shift almost periodic functions in shifts on T and we introduce some notation. Let $\alpha =\{\alpha_{n}\}\subset \Pi$ and $\beta =\{\beta_{n}\}\subset \Pi$ be two sequences. Then, β⊂α means that β is a subsequence of α; $\delta_{\pm }\left(\alpha ,\beta \right)=\left\{\delta_{\pm }\left(\alpha_{n},\beta_{n}\right)\right\};\delta_{-}\left(\alpha ,t_{0}\right)=\left\{\delta_{-}\left(\alpha_{n},t_{0}\right)\right\}$, α and β are common subsequences of $\alpha^{{}^{\prime }}$ and $\beta^{{}^{\prime }}$, respectively, means that $\alpha_{n}=\alpha_{n(k)}^{{}^{\prime }}$ and $\beta_{n}=\beta_{n(k)}^{{}^{\prime }}$ for some given function n(k).

We introduce the moving-operator $T^{S}$, $T_{\alpha }^{S}f\left(t,x\right)=g\left(t,x\right)$ by

$$g\left(t,x\right)=\lim_{n\to +\infty }f\left(\delta_{+}\left(\alpha_{n},t\right),x\right)$$

and is written only when the limit exists. The mode of convergence, e.g., pointwise, uniform, etc., will be specified at each use of the symbol.

In what follows, we establish some basic properties of *S*-almost periodic fuzzy vector-valued functions.

**Theorem** **61** ([50])**.** *Let T be a bi-direction S-CCTS with shifts $\delta_{\pm }$ and $f\in C(T\times D,R_{F}^{n})$ be S-almost periodic in t uniformly for x∈D, where $\delta_{+}\left(\tau ,t\right)$ is continuous in t. Then, it is uniformly continuous and bounded on $T^{*}\times S_{0}$.*

In the following, we obtained a shift-convergence theorem of *S*-almost periodic fuzzy vector-valued functions.

**Theorem** **62** ([50])**.** *Let $f\in C(T\times D,R_{F}^{n})$ be S-almost periodic in t uniformly for x∈D under shifts $\delta_{\pm }$. Then, for any given sequence $\alpha^{{}^{\prime }}\subset \Pi ,$ there exists a subsequence $\beta \subset \alpha^{{}^{\prime }}$ and $g\in C(T\times D,R_{F}^{n})$ such that $T_{\beta }^{S}f\left(t,x\right)=g\left(t,x\right)$ holds uniformly on $T^{*}\times S_{0}$ and g(t,x) is S-almost periodic in t uniformly for x∈D under shifts $\delta_{\pm }$.*

The concept of the *S*-hull of f(t,x) under shifts $\delta_{\pm }$ was introduced related to fuzzy almost periodic functions on time scales.

**Definition** **66** ([50])**.** *Let $f\in C(T\times D,R_{F}^{n})$. Then, $H_{S}\left(f\right)=\left\{g\left(t,x\right):T\times D\to R_{F}^{n}\right|$ and there exists α∈Π such that $T_{\alpha }^{S}f\left(t,x\right)=g\left(t,x\right)$ exists uniformly on $T^{*}\times S_{0}$} is called the S-hull of f(t,x) under shifts $\delta_{\pm }$.*

**Theorem** **63** ([50])**.** *$H_{S}\left(f\right)$ is compact if and only if f(t,x) is S-almost periodic in t uniformly for x∈D.*

**Theorem** **64** ([50])**.** *If $f\in C(T\times D,R_{F}^{n})$ is S-almost periodic in t uniformly for x∈D under shifts $\delta_{\pm }$, then, for any $g\left(t,x\right)\in H_{S}\left(f\right),H_{S}\left(f\right)=H_{S}\left(g\right)$.*

From Definition 66 and Theorem 64, one can directly obtain the following theorem.

**Theorem** **65** ([50])**.** *If $f\in C(T\times D,R_{F}^{n})$ is S-almost periodic in t uniformly for x∈D under shifts $\delta_{\pm }$, then, for any $g\left(t,x\right)\in H_{S}\left(f\right)$, g(t,x) is S-almost periodic in t uniformly for x∈D under shifts $\delta_{\pm }$.*

In what follows, a convergence theorem of *S*-almost periodic function sequences is established.

**Theorem** **66** ([50])**.** *If $f_{n}\in C\left(T\times D,R_{F}^{n}\right),n=1,2,\ldots$ are S-almost periodic in t for x∈D, and the sequence $\left\{f_{n}\left(t,x\right)\right\}$ uniformly converges to f(t,x) on $T^{*}\times S_{0}$, then f(t,x) is S-almost periodic in t uniformly for x∈D.*

**Theorem** **67** ([50])**.** *Let $f\in C(T\times D,R_{F}^{n})$ and j be an embedding mapping in Theorem 51. Then,*

(*i*) *j∘f is continuous on T if and only if f is continuous on T.*
(*ii*) *j∘f is S-almost periodic if and only if f is S-almost periodic.*
(*iii*) *If f is gH-*Δ*-differentiable on T, then j∘f is *Δ*-differentiable on T and ${\left(j\circ f\right)}^{\Delta }\left(t\right)=\left(j\circ f^{\Delta }\right)\left(t\right)$ for t∈T.*

**Theorem** **68** ([50])**.** *If $f\in C(T\times D,R_{F}^{n})$ is shift-*Δ*-almost periodic in t uniformly for x∈D under shifts $\delta_{\pm }$, denote*

$$F\left(t,x\right)=\int_{t_{0}}^{t}f\left(s,x\right)\Delta s,\;\;t_{0}\in T^{*},$$

*then F(t,x) is S-almost periodic in t uniformly for x∈D under shifts $\delta_{\pm }$ if and only if F(t,x) is bounded on $T^{*}\times S_{0}$, where $S_{0}$ is any compact subset of D.*

A sufficient and necessary criterion for *S*-almost periodic functions was established.

**Theorem** **69** ([50])**.** *A function $fC(T\times D,R_{F}^{n})$ is S-almost periodic in t uniformly for x∈D under shifts $\delta_{\pm }$ if and only if for every pair of sequences $\alpha^{{}^{\prime }},\beta^{{}^{\prime }}\subseteq \Pi$, there exist common subsequences $\alpha \subset \alpha^{{}^{\prime }},\beta \subset \beta^{{}^{\prime }}$ such that*

$$T_{\delta_{+}\left(\alpha ,\beta \right)}^{S}f\left(t,x\right)=T_{\alpha }^{S}T_{\beta }^{S}f\left(t,x\right).$$

## 4. The Quaternion Theory on Time Scales

To represent spatial orientations and rotations of elements in three-dimensional space, quaternions provide a convenient mathematical notation. Particularly, an axis-angle rotation about an arbitrary axis is encoded by the unit quaternion. In computer graphics, computer vision, robotics, navigation, molecular dynamics, flight dynamics, orbital mechanics of satellites and crystallographic texture analysis, rotation, and orientation quaternions have wide applications (see [54,55,56,57]).

**Figure 1:**
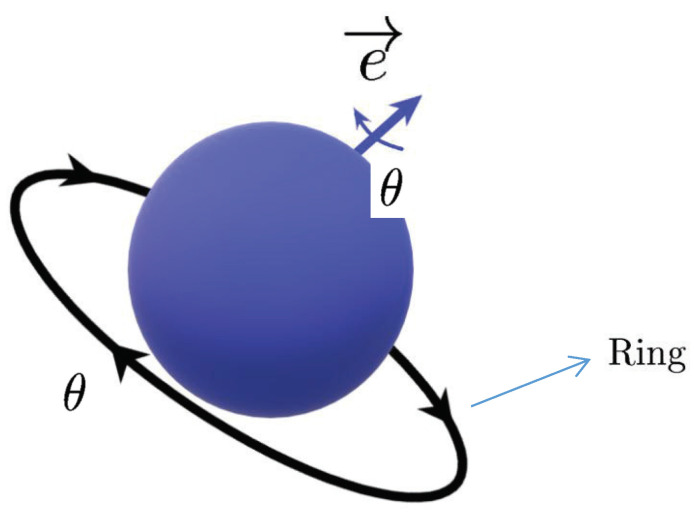
The quaternion number and the rotation of the corresponding ring.

**Figure 2:**
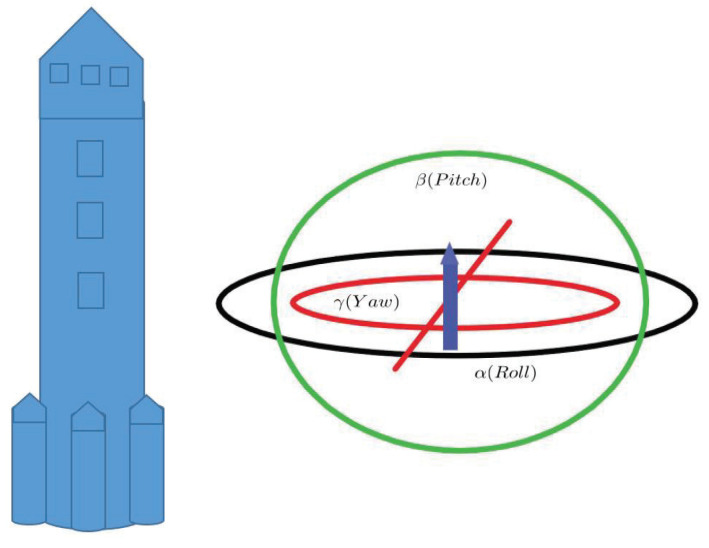
The phenomenon “Gimbal Lock”.

**Figure 3:**
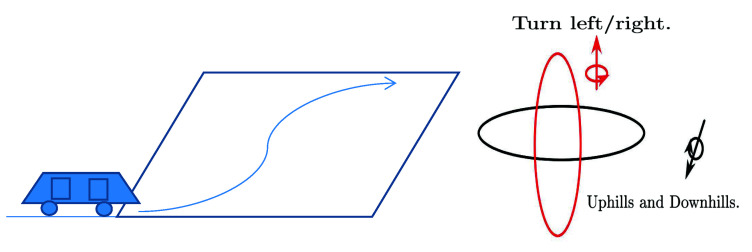
The 2×2 quaternion dynamic equations and the corresponding automobilism.

**Figure 4:**
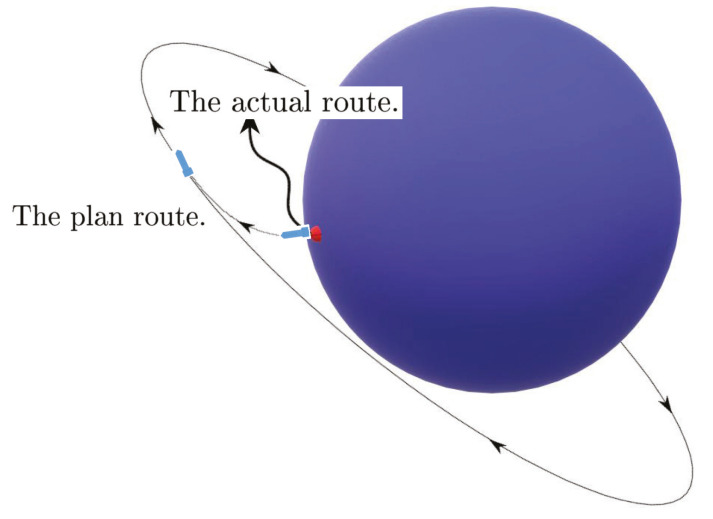
The obstacle of the presented quaternion exponential function application.

**Figure 5:**
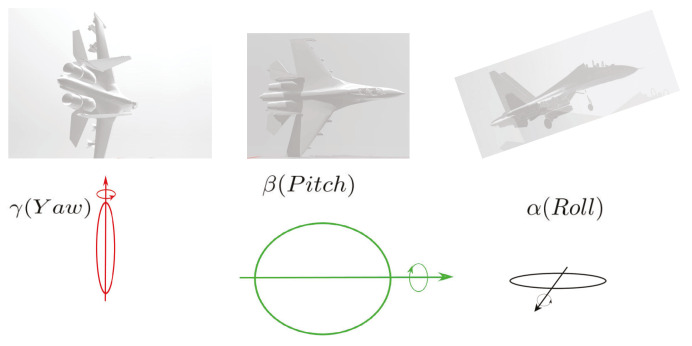
The 3×3 quaternion dynamic equations and the corresponding working diagram of a warplane.

The study of quaternion dynamic equations is an interesting topic (see [58,59]). In [60], Wang and Li firstly obtained the Cauchy matrix and Liouville formula of the quaternion impulsive dynamic equations on time scales. In [61], nine questions were proposed and solved in the quaternion dynamic equations on hybrid time scales as follows:

(1) By Euler’s rotation theory, one can represent a ring rotation through a corresponding quaternion (see Figure 1). However, if a rotation depends on a hybrid time domain, i.e., the ring’s rotation is intermittent, it is reasonable to consider the quaternion-valued functions on a time scale. It is difficult to describe the intermittent rotation by using a quaternion-valued functions on time scales.
(2) The direction of many conveyances are controlled by the gyroscope, for example, plane, ship, rocket, etc. The process of their motion is based on a time scale if the gyroscope does not work continuously. How should the work process of the gyroscope controlled by a 2×2 quaternion dynamic equation be depicted? When does the phenomenon "Gimbal Lock" take place (see Figure 2)? What is expression form of the solution to such quaternion dynamic equations?
(3) It is very common to see some phenomena described by a 2×2 quaternion dynamic equations on time scales. For example, in the process of a car going up a slope, the time that is consumed for changing the direction of the car can be regarded as a time scale which is located in the time interval from the bottom to the top of the hill (see Figure 3). It is convenient to use a 2×2 quaternion dynamic equations on a time scale to accurately describe the orientations and rotations of the car on the slope. How can a 2×2 quaternion dynamic equations to describe the process of the orientations and rotations of this car be established? What is the representation form of the solution to this dynamic equations?
(4) For the dynamic equation $x^{\Delta }\left(t\right)=f\left(t\right)x\left(t\right)$ with the initial value $x(t_{0})=1$. The quaternion exponential function
  $$E_{f}\left(t,s\right)=\exp\left(\int_{s}^{t}\xi_{\mu (\tilde{\tau })}(f\left(\tilde{\tau }\right)\Delta \tilde{\tau }\right)$$
  from the previous literature **is not a solution**, this deficiency will lead to a great difficulty to analyze some practical and theoretical problems. For example, the rocket will deviate from its intended route (see Figure 4). Therefore, it is urgent to find the quaternion exponential solution of this initial-valued problem.
(5) As is well known, three rings of the gyroscope work simultaneously such as warplane, rocket (see Figure 5), etc. Unfortunately, it is impossible to depict the orientations and rotations by a 2×2 quaternion dynamic equations for this case. Hence, it is necessary to consider the higher dimensional matrix quaternion dynamic equations. The main problem is how to establish some basic results of the 2×2 quaternion dynamic equations based on the double determinant algorithm and extend the case to n×n situation?
(6) Does the linear homogeneous n×n quaternion dynamic equations have a unique solution on time scales? What form does it have? In fact, many objects’ orientations and rotations can be described by n×n quaternion dynamic equations. If the solution is not unique, some reality problems will emerge such as losing the direction of the objects or suffering from the unexpected orientations and rotations.
(7) Letting X(t) be a solution of $X^{\Delta }\left(t\right)=A\left(t\right)X\left(t\right)$ and Y(t) be a solution of $Y^{\Delta }\left(t\right)=B\left(t\right)Y\left(t\right)$, what are the commutativity conditions of X(t) and Y(t) on time scales? Moreover, what is the connection between the quaternion functions with commutativity conditions and the complex-valued function? What are the commutativity conditions of the quaternion-valued functions on time scales?
(8) Based on the double determinant algorithm, what is the Liouville formula $Q_{TDE}\left(t\right)$ of the 2×2 linear homogenous quaternion dynamic equations on time scales? Particularly for $Q_{TDE}\left(t\right)=0$, what kind of the orientations and rotations phenomena will occur?
(9) We will encounter many problems in real applications in which the 2×2 or 3×3 quaternion dynamic equations are not sufficient. Taking the launching rocket as an example, the process will be affected by many factors, for example, the continuously changing earth gravity, the irregular wind power, the predictable and irregular air temperature and the continuously changing atmospheric pressure, etc. All these factors indicate that we must adopt the n×n quaternion dynamic equations on time scales. Therefore, some mathematical questions arise, such as what is the solution expression of the n×n quaternion dynamic equations $X^{\Delta }\left(t\right)=\Phi \left(t\right)X\left(t\right)$? Do these dynamic equations have a unique solution? How can the Liouville formula of the n×n quaternion dynamic equations on time scales be obtained?

### 4.1. Basic Results of Quaternion Dynamic Equations on Time Scales

In [61], the two-dimensional linear homogenous quaternion dynamic equations on time scales (or short for TQDEs) with the initial value were considered as follows:

(36) $$\left\{\begin{array}{c} h^{\Delta }\left(t\right)=\Phi \left(t\right)h\left(t\right),\\ h\left(t_{0}\right)=h_{0}\in H^{2}, \end{array}\right.$$

i.e.,

$$\left[\begin{array}{c} h_{1}^{\Delta }\left(t\right)\\ h_{2}^{\Delta }\left(t\right) \end{array}\right]=\left[\begin{array}{cc} p_{11}\left(t\right) & p_{12}\left(t\right)\\ p_{21}\left(t\right) & p_{22}\left(t\right) \end{array}\right]\left[\begin{array}{c} h_{1}\left(t\right)\\ h_{2}\left(t\right) \end{array}\right],$$

where $\Phi \left(\cdot \right):T\to H^{2\times 2}$ is an rd-continuous quaternion-valued function on T.

The following Liouville formula for (36) through double determinant algorithm was established.

**Theorem** **70** (Liouville Formula, [61])**.** *If τ is regressive for any t∈T, then the Wronskian $Q_{TDE}\left(t\right)$ of *(36)* satisfies the following quaternion Liouville formula:*

$$Q_{TDE}\left(t\right)=e_{\tau }\left(t,t_{0}\right)Q_{TDE}\left(t_{0}\right),$$

*where*

$$\begin{array}{ccc} \tau (t) & = & tr\Phi \left(t\right)+tr\Phi^{+}\left(t\right)+\left[tr\Phi \left(t\right)\bar{tr\Phi }\left(t\right)+\det_{r}\Phi \left(t\right)+\bar{\det_{r}\Phi }\left(t\right)\right]\mu \left(t\right)+\det_{d}\Phi \left(t\right)\mu^{3}\left(t\right)\\ & & +\left[(p_{11}\left(t\right)\bar{\det_{r}\Phi }\left(t\right)+\det_{r}\Phi \left(t\right)\bar{p_{11}}\left(t\right)+p_{22}\left(t\right)\bar{\det_{c}\Phi }\left(t\right)+\det_{c}\Phi \left(t\right)\bar{p_{22}}\left(t\right))\right]\mu^{2}\left(t\right) \end{array}$$

*and*

$$tr\Phi \left(t\right)=p_{11}\left(t\right)+p_{22}\left(t\right),\;\;\;tr\Phi^{+}\left(t\right)=\bar{p_{11}}\left(t\right)+\bar{p_{22}}\left(t\right),$$

$$\det_{r}\Phi =p_{11}\left(t\right)p_{22}\left(t\right)-p_{12}\left(t\right)p_{21}\left(t\right),\;\;\;\det_{c}\Phi \left(t\right)=p_{11}\left(t\right)p_{22}\left(t\right)-p_{21}\left(t\right)p_{12}\left(t\right).$$

**Definition** **67** ([61])**.** *Let $A\left(\cdot \right):T\to H^{n\times m}$, where $A\left(t\right)={\left[a_{wv}\left(t\right)\right]}_{n\times m}$, 1≤w≤n, 1≤v≤m. If every $a_{wv}\left(t\right)$ is rd-continuous, then A(t) is said to be an rd-continuous quaternion-valued matrix function.*

**Definition** **68** ([61])**.** *Let A(t),B(t) be n×n-quaternion-valued matrix function, A(t) and B(t) are rd-continuous on T, and define derivatives*

$$A^{\Delta }\left(t\right)={\left[a_{wv}^{\Delta }\left(t\right)\right]}_{1\leq w,v\leq n},\;\;\;B^{\Delta }\left(t\right)={\left[b_{wv}^{\Delta }\left(t\right)\right]}_{1\leq w,v\leq n}.$$

*Define the “circle plus" addition *⊕* as:*

A(t)⊕B(t)=A(t)+B(t)+μ(t)A(t)B(t).

**Definition** **69** ([61])**.** *Let f:T→H. We define the quaternion exponential function $e_{f}\left(t,t_{0}\right)$ by the solution of the initial value problem $x^{\Delta }\left(t\right)=f\left(t\right)x\left(t\right),x\left(t_{0}\right)=1$, and $e_{f}\left(t,t_{0}\right)$ can be given as*

$$e_{f}\left(t,t_{0}\right)=1+\sum_{n=1}^{+\infty }\int_{t_{0}}^{t}f\left(t_{n}\right)\int_{t_{0}}^{t_{n}}f\left(t_{n-1}\right)\ldots \int_{t_{0}}^{t_{2}}f\left(t_{1}\right)\Delta t_{1}\ldots \Delta t_{n-1}\Delta t_{n}.$$

*Similarly, let $\Phi :T\to H^{n\times n}$. The quaternion matrix exponential function $e_{\Phi }\left(t,t_{0}\right)$ is defined by the solution of the initial value problem $H^{\Delta }\left(t\right)=\Phi \left(t\right)H\left(t\right),H\left(t_{0}\right)=I$, where I is n×n-identity matrix, and $e_{\Phi }\left(t,t_{0}\right)$ can be given as*

$$e_{\Phi }\left(t,t_{0}\right)=I+\sum_{n=1}^{+\infty }\int_{t_{0}}^{t}\Phi \left(t_{n}\right)\int_{t_{0}}^{t_{n}}\Phi \left(t_{n-1}\right)\ldots \int_{t_{0}}^{t_{2}}\Phi \left(t_{1}\right)\Delta t_{1}\ldots \Delta t_{n-1}\Delta t_{n}.$$

Consider the *n*-dimensional linear homogenous TQDEs with the initial value as follows:

(37) $$\left\{\begin{array}{c} h^{\Delta }\left(t\right)=\hat{\Phi }\left(t\right)h\left(t\right),\\ h\left(t_{0}\right)=h_{0}\in H^{n}, \end{array}\right.$$

where $\hat{\Phi }\left(\cdot \right):T\to H^{n\times n}$ is an rd-continuous quaternion n×n-matrix function on T.

**Theorem** **71** ([61])**.** *If $\hat{\Phi }\left(t\right)$ is uniformly bounded on T, i.e., there exists some constant M>0 such that $∥\hat{\Phi }\left(t\right)∥\leq M$ for all t∈T, then the solution h(t) of the initial value problem of *(37)* is rd-continuous and uniquely given by*

$$h\left(t\right)=\left(I+\sum_{n=1}^{\infty }\int_{t_{0}}^{t}\hat{\Phi }\left(t_{n}\right)\int_{t_{0}}^{t_{n}}\hat{\Phi }\left(t_{n-1}\right)\ldots \int_{t_{0}}^{t_{2}}\hat{\Phi }\left(t_{1}\right)\Delta t_{1}\ldots \Delta t_{n-1}\Delta t_{n}\right)h_{0}.$$

In the following, we provide a numerical iteration method of the linear homogenous three-dimensional TQDEs on the time scale $T=\bar{2^{Z}}$.

**Example** **2.** *Let $T=\bar{2^{Z}}$, $t\in [2^{-10},2^{5}]$, the linear homogenous three-dimensional TQDEs with the initial value as follows:*

(38) $$h^{\Delta }\left(t\right)=\left[\begin{array}{ccc} \sin t^{2}+i\sin t+j\sin2t+k\cos t^{3} & \cos t+i\sin(t+1)+j\cos t+k\sin t & k\sin t\\ \sin2t+3i+2j+k\sin t & \sin4t+4i+j & j\sin t\\ 1+4i+j\cos t+k\sin t & \sin t+j\sin2t+3k & k\sin t^{2} \end{array}\right]h\left(t\right),$$

*with the initial value $h\left(2^{-10}\right)={\left[1,1,1\right]}^{T}$, where $h\left(t\right)=[h_{11}\left(t\right)+h_{12}\left(t\right)i+h_{13}\left(t\right)j+h_{14}\left(t\right)k,h_{21}\left(t\right)$$+h_{22}\left(t\right)i+h_{23}\left(t\right)j+h_{24}\left(t\right)k,h_{31}\left(t\right)+h_{32}\left(t\right)i+h_{33}\left(t\right)j+h_{34}{\left(t\right)k]}^{T}$ and $h^{\Delta }\left(t\right)=\left(A+Bi+Cj+Dk\right)h\left(t\right)$. The numerical solution of *(38)* can be solved by the following MATLAB code:*
*clear* 
*syms h11 h21 h31 h12 h22 h32 h13 h23 h33 h14 h24 h34 t;* 
*h11=1;h21=1;h31=1;h12=0;h22=0;h32=0;h13=0;h23=0;h33=0;h14=0;h24=0;h34=0;* 
*for n=-10:1:4;t=2.^n;* 
*h=[h11 h21 h31;h12 h22 h32;h13 h23 h33;h14 h24 h34];* 
A=[sin(t.^2) cos(t) 0;sin(2.*t) sin(4.*t) 0;1 sin(t) 0]’; 
*B=[sin(t) sin(t + 1) 0;3 4 0;4 0 0]’;* 
C=[sin(2.*t) cos(t) 0;2 1 sin(t);cos(t) sin(2.*t) 0]’; 
*D=[cos(t.^3) sin(t) sin(t);sin(t) 0 0;sin(t) 3 sin(t.^2)]’;* 
h=t.*[h(1,:)*A-h(2,:)*B-h(3,:)*C-h(4,:)*D;h(2,:)*A + h(1,:)*B + h(4,:)*C-h(3,:)*D; 
    h(3,:)*A-h(4,:)*B + h(1,:)*C + h(2,:)*D;h(4,:)*A + h(3,:)*B-h(2,:)*C + h(1,:)*D] + h 
*end* 
         
*The numerical iteration solution of *(38)* is given by Table 1. Notice that the existence of solutions to quaternion homogeneous dynamic equations on time scales provides a prerequisite to study the applications of quaternion dynamic equations on various hybrid domains, these significant applications are demonstrated in [61] including the multi-dimensional rotations and transformations of the submarine, gyroscope and planet whose dynamical behaviors are depicted by quaternion dynamics on time scales.*

Next, we will introduce a new Liouville algorithm of n×n quaternion-valued matrix which is an extension of the double determinant algorithm.

**Definition** **70** ([61])**.** *Let M be a n×n quaternion matrix, we define the Liouville algorithm of M by*

$${\mathrm{Liou}}_{dn}\left(M\right):=\prod_{w=1}^{n}\prod_{v=w+1}^{n}\det_{r}\left[\begin{array}{c} {\bar{M}}_{w}^{T}\\ {\bar{M}}_{v}^{T} \end{array}\right]\left[\begin{array}{cc} M_{w} & M_{v} \end{array}\right]=\prod_{w=1}^{n}\prod_{v=w+1}^{n}\det_{r}\left[\begin{array}{cc} \sum_{c=1}^{n}{\bar{m}}_{cw}m_{cw} & \sum_{c=1}^{n}{\bar{m}}_{cw}m_{cv}\\ \sum_{c=1}^{n}{\bar{m}}_{cv}m_{cw} & \sum_{c=1}^{n}{\bar{m}}_{cv}m_{cv} \end{array}\right],$$

*where $M=\left[M_{1},M_{2},\ldots ,M_{n}\right]={\left[m_{wv}\right]}_{n\times n}$.*

By Definition 70, the following conclusion is immediate.

**Remark** **9.** *${\mathrm{Liou}}_{dn}\left(M\right)=\det_{d}\left(M\right)$ for n=2.*

Next, we will show the Liouville algorithm of the n×n quaternion-valued matrix is well-defined, i.e., ${\mathrm{Liou}}_{dn}\left(M\right)$ is real.

**Theorem** **72** ([61])**.** *Let M be a n×n quaternion matrix, $M={\left[M_{wv}\right]}_{n\times n}$, n≥2, then ${\mathrm{Liou}}_{dn}\left(M\right)\in R$.*

Now, we will prove the Liouville formula of the linear homogenous n×n quaternion dynamic equations based on the fundamental matrix solution M(t) as follows.

Consider the n×n linear homogenous matrix TQDEs with the initial value as follows:

(39) $$\left\{\begin{array}{c} H^{\Delta }\left(t\right)=\hat{\Phi }\left(t\right)H\left(t\right),\\ H\left(t_{0}\right)=H_{0}\in H^{n\times n}. \end{array}\right.$$

**Theorem** **73** ([61])**.** *The Wronskian of (39) can be given as*

$$Q_{TDEn}\left(t\right)=\prod_{w=1}^{n}\prod_{v=w+1}^{n}\left(\sum_{c=1}^{n}{\bar{h}}_{cw}\left(t\right)h_{cw}\left(t\right)\sum_{c=1}^{n}{\bar{h}}_{cv}\left(t\right)h_{cv}\left(t\right)-\sum_{c=1}^{n}{\bar{h}}_{cw}\left(t\right)h_{cv}\left(t\right)\sum_{c=1}^{n}{\bar{h}}_{cv}\left(t\right)h_{cw}\left(t\right)\right).$$

### 4.2. Applied Quaternion Dynamic Equations

In Ref. [61], some real applications of the quaternion dynamic equations were demonstrated as follows.

**Figure 6:**
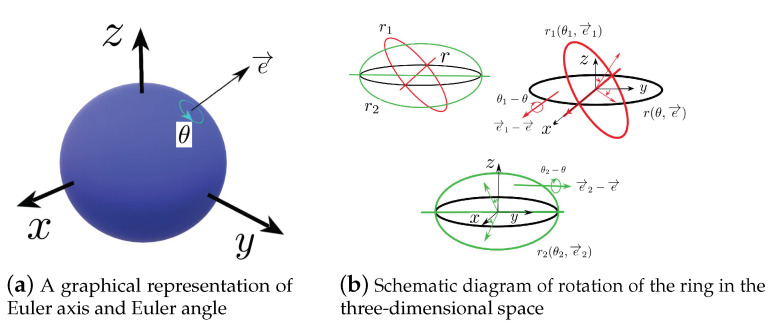
The diagram of the Euler’s rotation principle.

In a three-dimensional case, Euler’s rotation theory demonstrates that any rotation can be represented as a combination of a scalar θ (called the Euler angle) and a vector $\vec{e}$ (the direction vector of Euler axis) (see Figure 6a), which indicates that we can regard a quaternion number as the result of a point that is described by the shift of a vector $\vec{e}$ which starts at the origin of $R^{3}$ and the Euler angle θ which moves round $\vec{e}$, i.e., we can define q∈H as $q=q(\theta ,\vec{e})$. In a similar way, one can define the quaternion-valued matrix function $\hat{\Phi }\left(t\right)$ by

$$\hat{\Phi }\left(t\right)={\left[\begin{array}{cccc} q_{11}\left(\theta_{11}\left(t\right),{\vec{e}}_{11}\left(t\right)\right) & q_{12}\left(\theta_{12}\left(t\right),{\vec{e}}_{12}\left(t\right)\right) & \ldots & q_{1n}\left(\theta_{1n}\left(t\right),{\vec{e}}_{1n}\left(t\right)\right)\\ q_{21}\left(\theta_{21}\left(t\right),{\vec{e}}_{21}\left(t\right)\right) & q_{22}\left(\theta_{22}\left(t\right),{\vec{e}}_{22}\left(t\right)\right) & \ldots & q_{2n}\left(\theta_{2n}\left(t\right),{\vec{e}}_{2n}\left(t\right)\right)\\ \vdots & \vdots & \ddots & \vdots \\ q_{m1}\left(\theta_{m1}\left(t\right),{\vec{e}}_{m1}\left(t\right)\right) & q_{m2}\left(\theta_{m2}\left(t\right),{\vec{e}}_{m2}\left(t\right)\right) & \ldots & q_{mn}\left(\theta_{mn}\left(t\right),{\vec{e}}_{mn}\left(t\right)\right) \end{array}\right]}_{m\times n}.$$

Consider the rotation of a circular ring, there are two approaches to form this rotation, i.e., rotate $r(\theta ,\vec{e})$ to $r_{1}\left(\theta_{1},{\vec{e}}_{1}\right)$ or to $r_{2}\left(\theta_{2},{\vec{e}}_{2}\right)$ (see Figure 6b), which implies that we can represent the result of difference between two quaternion numbers as the rotation of a circular ring. Moreover, we can consider a quaternion dynamic equation

$$h^{\Delta }\left(t\right)=a\left(t\right)h\left(t\right),\;\mathrm{where}\;\;\;a:T\to H$$

with the initial value $h\left(t_{0}\right)=r\left(\theta ,\vec{e}\right)$ to track the rotation that is from $r(\theta ,\vec{e})$ to $r_{1}\left(\theta_{1},{\vec{e}}_{1}\right)$.

**Figure 7:**
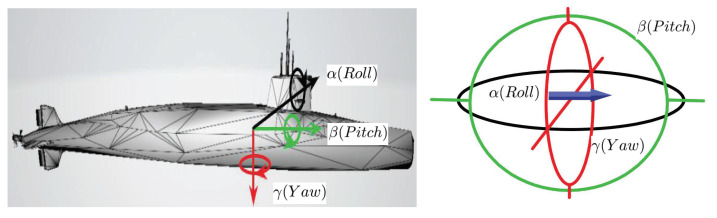
Initial state diagram of a submarine controlled by a gyroscope.

**Figure 8:**
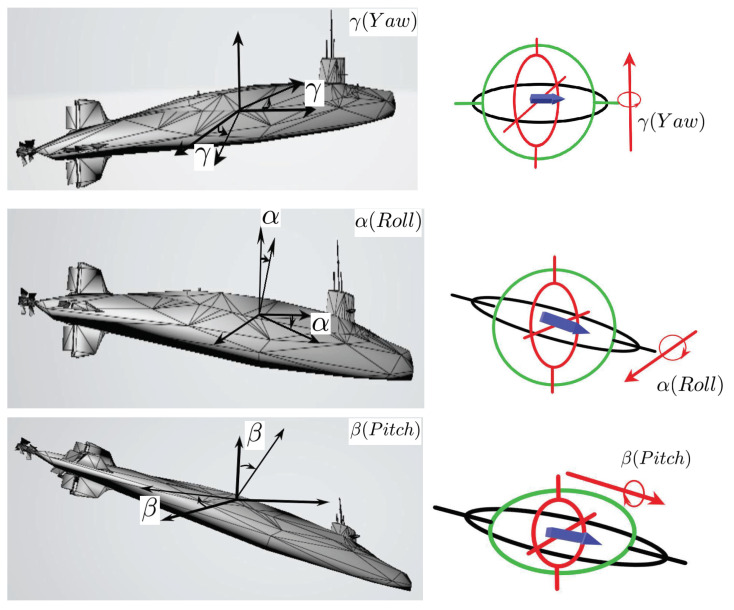
A working diagram of a submarine controlled by a gyroscope.

**Figure 9:**
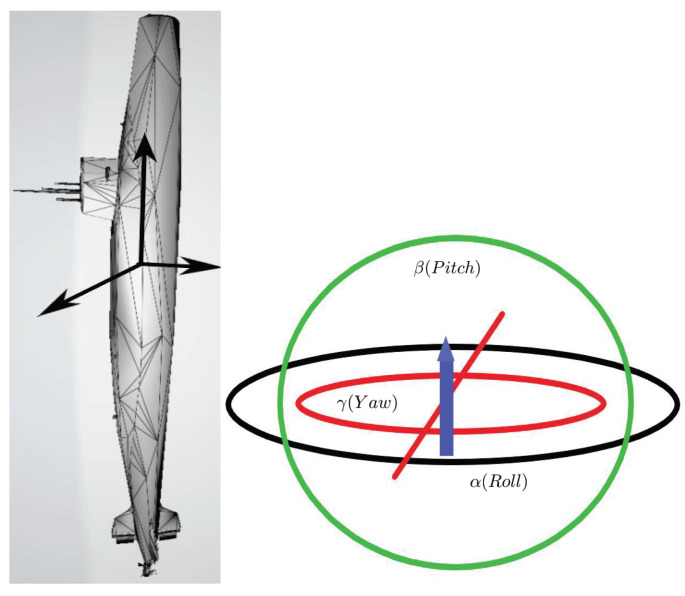
“Gimbal Lock”.

Next, some further results will be shown on the rotation of gyroscope. For the gyroscope, we shall consider this rotation in an ideal state with the rotations α(Roll), β(Pitch) and γ(Yaw)(see Figure 7). Noticing that the rotation dynamical behavior of the gyroscope is dependent on the operation of the three related rings, we can describe the rotation of gyroscope by the quaternion dynamic equations

(40) $$h^{\Delta }\left(t\right)=\hat{\Phi }\left(t\right)h\left(t\right)$$

with the initial value $h\left(t_{0}\right)={\left(h_{1}\left(t_{0}\right),h_{2}\left(t_{0}\right),h_{3}\left(t_{0}\right)\right)}^{T}$, where $\hat{\Phi }\left(t\right)$ is a 3×3 quaternion-valued matrix function, $h_{1}\left(t_{0}\right)$ is the quaternion number corresponding to the initial state of the α(Roll)-axis, $h_{2}\left(t_{0}\right)$ is the quaternion number corresponding to the initial state of the β(Pitch)-axis, $h_{3}\left(t_{0}\right)$ is the quaternion number corresponding to the initial state of the γ(Yaw)-axis. Indeed, the dynamical behavior of the submarine can be represented by the rotation of gyroscope (see Figure 8). Moreover, let ${\vec{e}}_{0}=\left(0,0,0\right)$, ${\vec{e}}_{1}=\left(0,0,1\right)$, ${\vec{e}}_{2}=\left(1,0,0\right)$,

$$\hat{\Phi }\left(t\right)=\left[\begin{array}{ccc} q_{1}\left(\theta_{1}\left(t\right),{\vec{e}}_{0}\right) & 0 & q_{3}\left(\theta_{3}\left(t\right),{\vec{e}}_{0}\right)\\ 0 & q_{2}\left(\theta_{2}\left(t\right),{\vec{e}}_{0}\right) & 0\\ q_{1}\left(\theta_{1}\left(t\right),{\vec{e}}_{0}\right) & 0 & q_{3}\left(\theta_{3}\left(t\right),{\vec{e}}_{0}\right) \end{array}\right],$$

with the initial value $h_{1}\left(t_{0}\right)=h_{3}\left(t_{0}\right)=h_{3}\left(\theta \left(t_{0}\right),{\vec{e}}_{1}\right)$ and $h_{2}\left(t_{0}\right)=h_{2}\left(\tilde{\theta }\left(t_{0}\right),{\vec{e}}_{2}\right)$. Then, $h_{1}\left(t\right)=h_{3}\left(t\right)=h_{3}\left(\theta \left(t\right),{\vec{e}}_{1}\right)$ and $h_{2}\left(t\right)=h_{2}\left(\tilde{\theta }\left(t\right),{\vec{e}}_{2}\right)$, a phenomenon of “Gimbal Lock” in Euler’s rotation principle indicates that there are two equivalent vector components in the vector solutions to the homogeneous equations (40). (see Figure 9). In the real applications, some monomer ships, including submarines, have a center of gravity and a center of buoyancy to maintain lateral stability, which indicates that we can consider the steering operation of submarines by the quaternion dynamic equations with the form (36).

**Figure 10:**
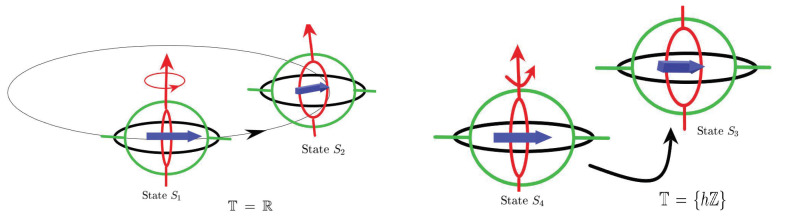
The gyroscope working diagram marked on different time scales.

Time scale plays a powerful role in dealing with the current problems under the quaternion background. For example, the gyroscope will move from the state $S_{1}$ to the state $S_{2}$ by a continuous rotational force for T=R; it may be also subjected to a discontinuous rotational force for T={hZ} and then revoking the force on R∖{hZ}, by inertia, the gyroscope will move from the state $S_{3}$ to the state $S_{4}$ (see Figure 10). The similar cases will frequently occur on the quantum time scales $T=\bar{q^{Z}}$ and the hybrid time scales such as $T=\left\{hZ\right\}\cup \left\{\bar{q^{Z}}\right\}$, etc. All these problems belong to the quaternion problems on time scales.

Commutativity of the quaternion-matrix-valued functions is an important property. For instance, a rotation can be denoted by an Euler angle θ and a unit vector defined by

$$\vec{u}=\left(u_{x},u_{y},u_{z}\right)=u_{x}i+u_{y}j+u_{z}k,$$

i.e., this rotation can be represented by a quaternion. In this paper, we have established some results of the commutativity of quaternion-valued functions. Based on it, two quaternion-valued functions can commutate with each other implies that the directional vectors of Euler axis are parallel to each other, which can contribute to studying the relationship between two particular status (or solutions) of the quaternion dynamic equations.

**Figure 11:**
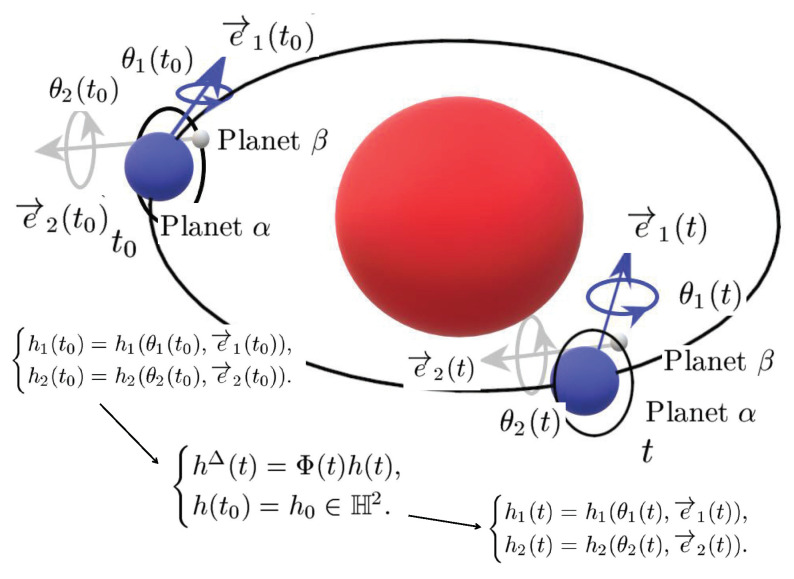
The motion diagram of the planet rotation described by (36) which describe (the state at $t_{0}$ to the state at *t* by (36)).

Another application is about the rotation of the planet. The rotation direction ${\vec{e}}_{1}\left(t_{0}\right)$ and the rotation angle $\theta_{1}\left(t_{0}\right)$ of the planet α at time $t_{0}$ describe the space state of the planet α at $t_{0}$, i.e., a quaternion number $h_{1}\left(\theta_{1}\left(t_{0}\right),{\vec{e}}_{1}\left(t_{0}\right)\right)$ represents the state. Similarly, we can consider the planet β at time $t_{0}$ and planets α,β at time *t* as well. By using the similar analysis of the gyroscope above, the rotation of two planets have an impact on each other, thus we can use dynamic Equation (36) to depict such a rotation which is from the state at time $t_{0}$ to the state at time *t* (see Figure 11). Notice that the dynamic Equation (36) can be given as:

$$h^{\Delta }\left(t\right)=\Phi \left(t\right)h\left(t\right)$$

i.e.,

$$\left[\begin{array}{c} h_{1}^{\Delta }\left(\theta_{1}\left(t\right),{\vec{e}}_{1}\left(t\right)\right)\\ h_{2}^{\Delta }\left(\theta_{2}\left(t\right),{\vec{e}}_{2}\left(t\right)\right) \end{array}\right]=\left[\begin{array}{cc} q_{11}\left(\theta_{11}\left(t\right),{\vec{e}}_{11}\left(t\right)\right) & q_{12}\left(\theta_{12}\left(t\right),{\vec{e}}_{12}\left(t\right)\right)\\ q_{21}\left(\theta_{21}\left(t\right),{\vec{e}}_{21}\left(t\right)\right) & q_{22}\left(\theta_{22}\left(t\right),{\vec{e}}_{22}\left(t\right)\right) \end{array}\right]\left[\begin{array}{c} h_{1}\left(\theta_{1}\left(t\right),{\vec{e}}_{1}\left(t\right)\right)\\ h_{2}\left(\theta_{2}\left(t\right),{\vec{e}}_{2}\left(t\right)\right) \end{array}\right],$$

with the initial condition

$$h\left(t_{0}\right)=\left[\begin{array}{c} h_{1}\left(t_{0}\right)\\ h_{2}\left(t_{0}\right) \end{array}\right]=\left[\begin{array}{c} h_{1}\left(\theta_{1}\left(t_{0}\right),{\vec{e}}_{1}\left(t_{0}\right)\right)\\ h_{2}\left(\theta_{2}\left(t_{0}\right),{\vec{e}}_{2}\left(t_{0}\right)\right) \end{array}\right].$$

In what follows, a rotation of the planets α,β by a concrete dynamic equation is demonstrated, and the state of the planet at the same time of each day is considered. For this case, the time intervals that we assume are equivalent. Therefore, we consider the dynamic equations on the time scale T=Z as follows (see Example 3).

**Example** **3** ([61])**.** *Letting T=Z, we consider the linear homogenous two-dimensional TQDEs as follows:*

(41) $$h^{\Delta }\left(t\right)=\Phi \left(t\right)h\left(t\right),\;\;\;\Phi \left(t\right)=A+Bi+Cj+Dk=\left[\begin{array}{cc} \Phi_{11} & \Phi_{12}\\ \Phi_{21} & \Phi_{22} \end{array}\right],$$

*with the initial value $h\left(0\right)={\left[1,1\right]}^{T}$, where*

$$\left\{\begin{array}{c} \Phi_{11}=15\sin t\sin23.5+15i\cos t\sin23.5+15j\sin t\cos23.5+15k\cos t\sin23.5,\\ \Phi_{12}=15\sin^{2}t\sin23.5+15i\cos t\sin t\sin23.5+15j\sin t\cos t\cos23.5+15k\cos^{2}t\sin23.5,\\ \Phi_{21}=3.8\sin t+3.8i\cos t+2j\sin t+2k\cos t,\\ \Phi_{22}=3.8\sin^{2}t+3.8i\cos t\sin t+2j\sin t\cos t+2k\cos^{2}t. \end{array}\right.$$

*$h\left(t\right)={\left[h_{11}\left(t\right)+h_{12}\left(t\right)i+h_{13}\left(t\right)j+h_{14}\left(t\right)k,h_{21}\left(t\right)+h_{22}\left(t\right)i+h_{23}\left(t\right)j+h_{24}\left(t\right)k\right]}^{T}=\left[{\hat{h}}_{1}\left(t\right),{\hat{h}}_{2}\left(t\right)\right]$. Assume that $h\left(t\right)=h_{1}+h_{2}i+h_{3}j+h_{4}k$, then*

$$\begin{array}{ccc} h^{T}\left(t+1\right) & = & h^{T}\left(t\right)\Phi^{T}\left(t\right)+h^{T}\left(t\right)=h_{1}^{T}A^{T}-h_{2}^{T}B^{T}-h_{3}^{T}C^{T}-h_{4}^{T}D^{T}+\left(h_{2}^{T}A^{T}+h_{1}^{T}B^{T}+h_{4}^{T}C^{T}-h_{3}^{T}D^{T}\right)i\\ & & +\left(h_{3}^{T}A^{T}-h_{4}^{T}B^{T}+h_{1}^{T}C^{T}+h_{2}^{T}D^{T}\right)j+\left(h_{4}^{T}A^{T}+h_{3}^{T}B^{T}-h_{2}^{T}C^{T}+h_{1}^{T}D^{T}\right)k+h^{T}\left(t\right), \end{array}$$

*i.e.,*

$$\left\{\begin{array}{c} h_{1}^{T}\left(t+1\right)=h_{1}^{T}A^{T}-h_{2}^{T}B^{T}-h_{3}^{T}C^{T}-h_{4}^{T}D^{T}+h_{1}^{T},\\ h_{2}^{T}\left(t+1\right)=h_{2}^{T}A^{T}+h_{1}^{T}B^{T}+h_{4}^{T}C^{T}-h_{3}^{T}D^{T}+h_{2}^{T},\\ h_{3}^{T}\left(t+1\right)=h_{3}^{T}A^{T}-h_{4}^{T}B^{T}+h_{1}^{T}C^{T}+h_{2}^{T}D^{T}+h_{3}^{T},\\ h_{4}^{T}\left(t+1\right)=h_{4}^{T}A^{T}+h_{3}^{T}B^{T}-h_{2}^{T}C^{T}+h_{1}^{T}D^{T}+h_{4}^{T}, \end{array}\right.$$

*where $h\left(t+1\right)=h_{1}\left(t+1\right)+h_{2}\left(t+1\right)i+h_{3}\left(t+1\right)j+h_{4}\left(t+1\right)k$, $h_{wv}\in R$, $h_{v},h_{v}\left(t+1\right)\in R^{2}$, w,v∈{1,2,3,4} and $A,B,C,D\in R^{2\times 2}$. Hence, the numerical solution of *(41)* can be calculated by the following MATLAB code:*
*clear*  
*syms h11 h21 h12 h22 h13 h23 h14 h24 t;*  
*h11=1;h21=1;h12=0;h22=0;h13=0;h23=0;h14=0;h24=0;*  
*for n=0:1:14;t=n*  
*h=[h11 h21;h12 h22;h13 h23;h14 h24];*  
A=[15*sin(23.5)*sin(t) 15*sin(t)*sin(t)*sin(23.5);  
   3.8*sin(t) 3.8*sin(t)*sin(t)]’;  
B=[15*sin(23.5)*cos(t) 15*cos(t)*sin(t)*sin(23.5);  
  3.8*cos(t) 3.8*cos(t)*sin(t)]’;  
C=[15*cos(23.5)*sin(t) 15*sin(t)*cos(t)*cos(23.5);2*sin(t) 2*sin(t)*cos(t)]’;  
D=[15*sin(23.5)*cos(t) 15*cos(t)*cos(t)*sin(23.5);2*cos(t) 2*cos(t)*cos(t)]’;  
h=1.*[h(1,:)*A-h(2,:)*B-h(3,:)*C-h(4,:)*D;h(2,:)*A + h(1,:)*B + h(4,:)*C-h(3,:)*D;  
    h(3,:)*A-h(4,:)*B + h(1,:)*C + h(2,:)*D;h(4,:)*A + h(3,:)*B-h(2,:)*C + h(1,:)*D] + h  
*end*  
           
*The numerical solution of *(41)* is demonstrated at Table 2. Next, in real application, we will show the solution h(t) with the planets α,β corresponding state (see Figure 11), without loss of generality, for t=10, we have*

$$\begin{array}{ccc} h(10) & = & \left[\begin{array}{c} -7.7127+19.2623i-0.0340j+1.2122k\\ 3.2114-4.8892i+0.0732j-0.1619k \end{array}\right]=\left[\begin{array}{c} {\hat{h}}_{1}\left(10\right)\\ {\hat{h}}_{2}\left(10\right) \end{array}\right]\\ & = & \left[\begin{array}{c} |{\hat{h}}_{1}\left(10\right)|\left[\cos\theta_{1}\left(10\right)+\left(i,j,k\right){\vec{e}}_{1}\left(10\right)\sin\theta_{1}\left(10\right)\right]\\ |{\hat{h}}_{2}\left(10\right)|\left[\cos\theta_{2}\left(10\right)+\left(i,j,k\right){\vec{e}}_{2}\left(10\right)\sin\theta_{2}\left(10\right)\right] \end{array}\right]=\left[\begin{array}{c} |{\hat{h}}_{1}\left(10\right)|\left[\frac{R({\hat{h}}_{1}\left(10\right))}{|{\hat{h}}_{1}\left(10\right)|}+\frac{ℑ({\hat{h}}_{1}\left(10\right))}{\left|ℑ({\hat{h}}_{1}\left(10\right))\right|}\frac{\left|ℑ({\hat{h}}_{1}\left(10\right))\right|}{|{\hat{h}}_{1}\left(10\right)|}\right]\\ |{\hat{h}}_{2}\left(10\right)|\left[\frac{R({\hat{h}}_{2}\left(10\right))}{|{\hat{h}}_{2}\left(10\right)|}+\frac{ℑ({\hat{h}}_{2}\left(10\right))}{\left|ℑ({\hat{h}}_{2}\left(10\right))\right|}\frac{\left|ℑ({\hat{h}}_{2}\left(10\right))\right|}{|{\hat{h}}_{2}\left(10\right)|}\right] \end{array}\right]\\ & = & \left[\begin{array}{c} 20.78443\left(\frac{-7.7127}{20.78443}+\frac{19.2623i-0.0340j+1.2122k}{19.30043}\frac{19.30043}{20.78442}\right)\\ 5.88431\left(\frac{3.2114}{5.88431}+\frac{-4.8892i+0.0732j-0.1619k}{4.93072}\frac{4.93072}{5.88431}\right) \end{array}\right], \end{array}$$

*i.e., the rotation direction of the planet α in three-dimensional space is ${\vec{e}}_{1}\left(10\right)=(0.99802,-0.00176,$0.06281) and the rotation angle is $\theta_{1}\left(10\right)$, where $\cos\theta_{1}\left(10\right)=-0.37108$ and $\sin\theta_{1}\left(10\right)=0.9286$. Similarly, the rotation direction of the planet β is ${\vec{e}}_{2}\left(10\right)=\left(-0.99158,0.00754,-0.03283\right)$ and the rotation angle is $\theta_{2}\left(10\right)$, where $\cos\theta_{2}\left(10\right)=0.54575$ and $\sin\theta_{2}\left(10\right)=0.83794$.*

In the following, a comprehensive application is provided including the rotation theory of quaternions, the Liouville formula, the commutativity of quaternion-matrix-valued functions, the existence and uniqueness of solution for TQDEs, and the quaternion exponential function, and we apply the theory of time scales to show the feasibility of the main results stated in this article.

**Example** **4** ([61])**.** *In this application, we will consider the motion of submarines by the quaternion dynamic equations under time scales background. We use $h_{1}\left(t\right)$ to represent the orientations and rotations of α(Roll), $h_{2}\left(t\right)$ to represent the orientations and rotations of γ(Yaw) (see Figure 8). Since the submarines have a center of gravity and a center of buoyancy to maintain lateral stability, the function β(Picth) is a constant, which means that we can use *(36)* to present this submarine’s motion. The initial value $h\left(t_{0}\right)={\left[1,k\right]}^{T}$ represents the initial state of the orientations and rotations of the submarine (see Figure 7). For convenience, the black ring is called roll ring, and the red ring is called yaw ring in Figure 7. Indeed, $p_{11}\left(t\right)$ represents the difference value of the roll ring variable. We take $p_{11}\left(t\right)=t-1+2t\cos\frac{\lambda \pi }{2}i+2t\sin\frac{\lambda \pi }{2}k$, which implies the roll ring rotates left for λ=0, upward for λ=1. During the voyage of the submarine, the roll ring is affected by the yaw ring. Hence, we take $p_{12}\left(t\right)=t$, i.e., the yaw ring changes the speed of the roll ring instead of its direction. For the yaw ring, it is not subject to the effect of the roll ring. Hence, we take $p_{21}\left(t\right)=0$ and $p_{22}\left(t\right)=t-1+3tk$. On the other hand, if $Q_{TDE}\left(t\right)=0$, then $h_{1}\left(t\right)$ and $h_{2}\left(t\right)$ are right dependent, i.e., the roll ring and the yaw ring are in the same plane. Furthermore, for $h_{1}\left(\theta_{1}\left(t\right),{\vec{e}}_{1}\left(t\right)\right)$ and $h_{2}\left(\theta_{2}\left(t\right),{\vec{e}}_{2}\left(t\right)\right)$, if ${\vec{e}}_{1}\left(t\right),{\vec{e}}_{2}\left(t\right)$ are parallel to each other and they are perpendicular to the horizon simultaneously, then the phenomenon of “Gimbal Lock" happens. For $Q_{TDE}\left(t\right)\neq 0$, $h_{1}\left(t\right)$ and $h_{2}\left(t\right)$ are right independent, i.e., the roll ring and the yaw ring are not in the same plane.*

**Figure 12:**
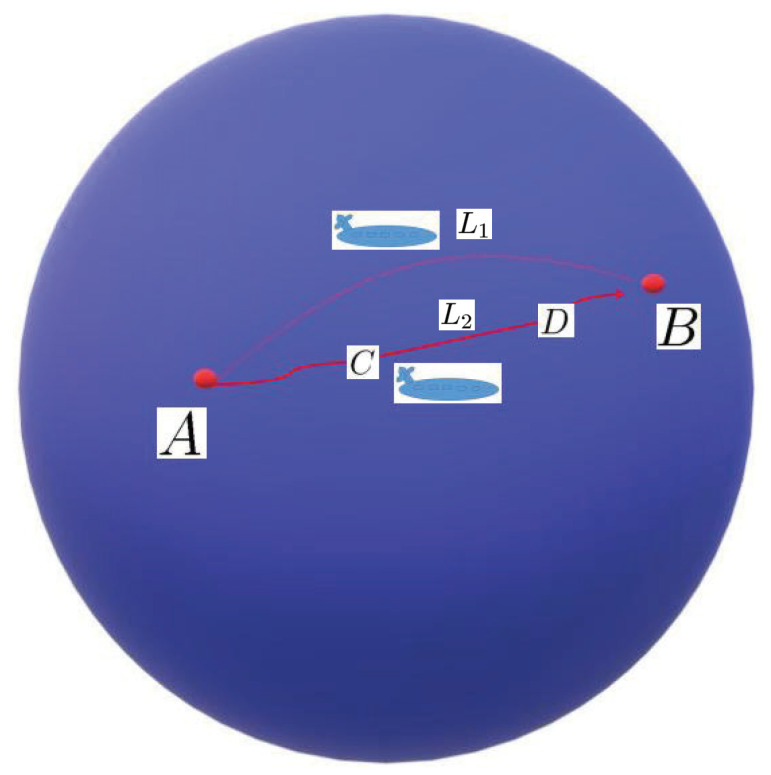
The motion diagram of submarine.

*As the quaternion dynamic equations are considered on times scales, we shall show the influence of time scales for the motion of submarine as follows. If we steer the submarines from the place A to the place B, there are two routes that can be chosen, i.e., $L_{1}$ or $L_{2}$ (see Figure 12). For the route $L_{1}$, we steer the submarine in an ideal state, i.e., the orientations and rotations of the submarine are continuously changed by considering the corresponding quaternion dynamic equations in T=R case. For the route $L_{2}$, we steer the submarine from the place A to the place C by the continuous change of the orientations and rotations of the submarine, then steer straight ahead from the place C to the place D, which indicates that the corresponding quaternions value are different at the places A and C, and are equivalent at the places C and D. We denote the interval $\left[t_{0},t\right]$ the time of passing places AB. Obviously, the time that is consumed to change the orientations and rotations of the submarine is a closed subset of $\left[t_{0},t\right]$, i.e., the corresponding quaternion dynamic equations are considered on $T\cap [t_{0},t]$, which is a time scale. Now, we will calculate the solution, the fundamental matrix, and the Liouville formula for the T=Z case.*

*Let λ∈[0,1], T=Z, $t_{0}=1$, $\Phi \left(t\right)=\left[\begin{array}{cc} t-1+2ti\cos\frac{\lambda \pi }{2}+2tk\sin\frac{\lambda \pi }{2} & t\\ 0 & t-1+3tk \end{array}\right]$, the initial value $h\left(t_{0}\right)={\left[1,k\right]}^{T}$. Then, the solution of *(36)* can be given as*

$$\begin{array}{ccc} h(t) & = & e_{\Phi }\left(t,1\right)h\left(1\right)=h\left(1\right)+\sum_{n=1}^{+\infty }\int_{1}^{t}\Phi \left(t_{n}\right)\int_{1}^{t_{n}}\Phi \left(t_{n-1}\right)\ldots \int_{1}^{t_{2}}\Phi \left(t_{1}\right)\Delta t_{1}\ldots \Delta t_{n-1}\Delta t_{n}h\left(1\right)\\ & = & h\left(1\right)+\sum_{n=1}^{t}\int_{1}^{t}\Phi \left(t_{n}\right)\int_{1}^{t_{n}}\Phi \left(t_{n-1}\right)\ldots \int_{1}^{t_{2}}\Phi \left(t_{1}\right)\Delta t_{1}\ldots \Delta t_{n-1}\Delta t_{n}h\left(1\right)\\ & = & \left\{I+\Phi (t-1)+\Phi (t-2)+\ldots +\Phi (1)+\Phi (t-1)[\Phi (t-2)+\ldots +\Phi (1)\right]\\ & & +\Phi (t-2)[\Phi (t-3)+\ldots +\Phi (1)]+\ldots +\Phi (t-1)\Phi (t-2)\ldots \Phi (1)\}h(1)\\ & = & \left[I+\Phi (t-1)][I+\Phi (t-2)]\ldots [I+\Phi (1)]h(1\right)\\ & = & \left[\begin{array}{cc} \left(t-1\right)!{\left(1+i\cos\frac{\lambda \pi }{2}+k\sin\frac{\lambda \pi }{2}\right)}^{t-1} & \left(t-1\right)!\sum_{l=0}^{t-1}{\left(1+i\cos\frac{\lambda \pi }{2}+k\sin\frac{\lambda \pi }{2}\right)}^{l}{\left[1+3k\right]}^{t-1-l}\\ 0 & \left(t-1\right)!{\left(1+3k\right)}^{t-1} \end{array}\right]h\left(1\right)\\ & = & \left[\begin{array}{c} \left(t-1\right)!{\left(1+i\cos\frac{\lambda \pi }{2}+k\sin\frac{\lambda \pi }{2}k\right)}^{t-1}+k\left(t-1\right)!\sum_{l=0}^{t-1}{\left(1+i\cos\frac{\lambda \pi }{2}+k\sin\frac{\lambda \pi }{2}\right)}^{l}{\left[1+3k\right]}^{t-1-l}\\ \left(t-1\right)!{\left(1+3k\right)}^{t-1}k \end{array}\right]. \end{array}$$

*We say that λ is the steering parameter, i.e., through taking the different values of λ, one can control the submarine’s motion by choosing the corresponding parameter that reflects the different submarine’s states. Assume that*

$$\begin{array}{ccc} h(t) & = & \left[\begin{array}{c} h_{1}\left(t\right)\\ h_{2}\left(t\right) \end{array}\right]=\left[\begin{array}{c} |h_{1}\left(t\right)|\left(\frac{R(h_{1}\left(t\right))}{|h_{1}\left(t\right)|}+\frac{ℑ(h_{1}\left(t\right))}{\left|ℑ(h_{1}\left(t\right))\right|}\frac{\left|ℑ(h_{1}\left(t\right))\right|}{|h_{1}\left(t\right)|}\right)\\ |h_{2}\left(t\right)|\left(\frac{R(h_{2}\left(t\right))}{|h_{2}\left(t\right)|}+\frac{ℑ(h_{2}\left(t\right))}{\left|ℑ(h_{2}\left(t\right))\right|}\frac{\left|ℑ(h_{2}\left(t\right))\right|}{|h_{2}\left(t\right)|}\right) \end{array}\right]\\ & = & \left[\begin{array}{c} |h_{1}\left(t\right)|\left(\cos\mathrm{Arg}\left(h_{1}\left(t\right)\right)+\frac{ℑ(h_{1}\left(t\right))}{\left|ℑ(h_{1}\left(t\right))\right|}\sin\mathrm{Arg}\left(h_{1}\left(t\right)\right)\right)\\ |h_{2}\left(t\right)|\left(\cos\mathrm{Arg}\left(h_{2}\left(t\right)\right)+\frac{ℑ(h_{2}\left(t\right))}{\left|ℑ(h_{2}\left(t\right))\right|}\sin\mathrm{Arg}\left(h_{2}\left(t\right)\right)\right) \end{array}\right]\\ & = & \left[\begin{array}{c} |h_{1}\left(t\right)|\left(\cos\theta_{1}\left(t\right)+{\vec{e}}_{1}\left(t\right)\left(i,j,k\right)\sin\theta_{1}\left(t\right)\right)\\ |h_{2}\left(t\right)|\left(\cos\theta_{2}\left(t\right)+{\vec{e}}_{2}\left(t\right)\left(i,j,k\right)\sin\theta_{2}\left(t\right)\right) \end{array}\right]\\ & = & \left[\begin{array}{c} h_{10}\left(t\right)+h_{11}\left(t\right)i+h_{12}\left(t\right)j+h_{13}\left(t\right)k\\ h_{20}\left(t\right)+h_{21}\left(t\right)i+h_{22}\left(t\right)j+h_{23}\left(t\right)k \end{array}\right]. \end{array}$$

*For λ∈[0,1), $h_{1}\left(t\right)$ and $h_{2}\left(t\right)$ are non-commutative. The roll ring rotates to the left for λ=0, and it rotates to left and upward at the same time for λ∈(0,1). For λ=1, we have*

$$h\left(t\right)=\left[\begin{array}{c} \left(t-1\right)!{\left[1+k\right]}^{t-1}+k\left(t-1\right)!\sum_{l=0}^{t-1}{\left(1+k\right)}^{l}{\left[1+3k\right]}^{t-1-l}\\ \left(t-1\right)!{\left(1+3k\right)}^{t-1}k \end{array}\right],$$

*thus*

$$\left\{\begin{array}{c} h_{11}\left(t\right)h_{22}\left(t\right)=h_{12}\left(t\right)h_{21}\left(t\right)\\ h_{12}\left(t\right)h_{23}\left(t\right)=h_{13}\left(t\right)h_{22}\left(t\right)\\ h_{11}\left(t\right)h_{23}\left(t\right)=h_{13}\left(t\right)h_{21}\left(t\right) \end{array}\right.,$$

*${\vec{e}}_{1}\left(t\right),{\vec{e}}_{2}\left(t\right)\in \left\{\left(0,0,1\right),\left(0,0,0\right)\right\}$. Hence, $h_{1}\left(t\right),h_{2}\left(t\right)$ are commutative and ${\vec{e}}_{1}\left(t\right),{\vec{e}}_{2}\left(t\right)$ are parallel vectors. Moreover, if $Q_{TDE}\left(t_{0}\right)=0$, ${\vec{e}}_{1}\left(t_{0}\right)={\vec{e}}_{2}\left(t_{0}\right)=\left(0,0,1\right)$, then ${\vec{e}}_{1}\left(t\right),{\vec{e}}_{2}\left(t\right)$ are perpendicular to the horizontal plane and the phenomenon of "Gimbal Lock" happens. The fundamental solution matrix can be formulated as*

$$M\left(t\right)=\left[\begin{array}{cc} \left(t-1\right)!{\left(1+i\cos\frac{\lambda \pi }{2}+k\sin\frac{\lambda \pi }{2}\right)}^{t-1} & \left(t-1\right)!\sum_{l=0}^{t-1}{\left(1+i\cos\frac{\lambda \pi }{2}+k\sin\frac{\lambda \pi }{2}\right)}^{l}{\left[1+3k\right]}^{t-1-l}\\ 0 & \left(t-1\right)!{\left(1+3k\right)}^{t-1} \end{array}\right].$$

*By Theorem 70, we have*

$$\begin{array}{ccc} \tau (t) & = & p_{11}\left(t\right)+\bar{p_{11}}\left(t\right)+p_{22}\left(t\right)+\bar{p_{22}}\left(t\right)+[p_{11}\left(t\right)\bar{p_{11}}\left(t\right)+p_{22}\left(t\right)\bar{p_{22}}\left(t\right)+\left(p_{11}\left(t\right)+\bar{p_{11}}\left(t\right)\right)\left(p_{22}\left(t\right)+\bar{p_{22}}\left(t\right)\right)\\ & & -\left(p_{12}\left(t\right)p_{21}\left(t\right)+\bar{p_{21}}\left(t\right)\bar{p_{12}}\left(t\right)\right)]\mu \left(t\right)+[p_{11}\left(t\right)\bar{p_{11}}\left(t\right)\left(p_{22}\left(t\right)+\bar{p_{22}}\left(t\right)\right)+\left(p_{11}\left(t\right)+\bar{p_{11}}\left(t\right)\right)p_{22}\left(t\right)\bar{p_{22}}\left(t\right)\\ & & -\left(p_{11}\left(t\right)\bar{p_{21}}\left(t\right)\bar{p_{12}}\left(t\right)+p_{12}\left(t\right)p_{21}\left(t\right)\bar{p_{11}}\left(t\right)\right)-\left(p_{12}\left(t\right)\bar{p_{22}}\left(t\right)p_{21}\left(t\right)+\bar{p_{21}}\left(t\right)p_{22}\left(t\right)\bar{p_{12}}\left(t\right)\right)]\mu^{2}\left(t\right)\\ & & +[p_{11}\left(t\right)\bar{p_{11}}\left(t\right)p_{22}\left(t\right)\bar{p_{22}}\left(t\right)+p_{12}\left(t\right)\bar{p_{12}}\left(t\right)p_{21}\left(t\right)\bar{p_{21}}\left(t\right)\\ & & -p_{12}\left(t\right)\bar{p_{22}}\left(t\right)p_{21}\left(t\right)\bar{p_{11}}\left(t\right)-p_{11}\left(t\right)\bar{p_{21}}\left(t\right)p_{22}\left(t\right)\bar{p_{12}}\left(t\right)]\mu^{3}\left(t\right)\\ & = & p_{11}\left(t\right)+\bar{p_{11}}\left(t\right)+p_{22}\left(t\right)+\bar{p_{22}}\left(t\right)+p_{11}\left(t\right)\bar{p_{11}}\left(t\right)+p_{22}\left(t\right)\bar{p_{22}}\left(t\right)+\left(p_{11}\left(t\right)+\bar{p_{11}}\left(t\right)\right)\left(p_{22}\left(t\right)+\bar{p_{22}}\left(t\right)\right)\\ & & +p_{11}\left(t\right)\bar{p_{11}}\left(t\right)\left(p_{22}\left(t\right)+\bar{p_{22}}\left(t\right)\right)+\left(p_{11}\left(t\right)+\bar{p_{11}}\left(t\right)\right)p_{22}\left(t\right)\bar{p_{22}}\left(t\right)+p_{11}\left(t\right)\bar{p_{11}}p_{22}\left(t\right)\bar{p_{22}}\left(t\right)\\ & = & 2t-2+2t-2+{\left(t-1\right)}^{2}+4t^{2}+{\left(t-1\right)}^{2}+9t^{2}+4{\left(t-1\right)}^{2}+\left[{\left(t-1\right)}^{2}+4t^{2}\right]\left(2t-2\right)\\ & & +\left[{\left(t-1\right)}^{2}+9t^{2}\right]\left(2t-2\right)+\left[{\left(t-1\right)}^{2}+4t^{2}\right]\left[{\left(t-1\right)}^{2}+9t^{2}\right]=15t^{4}-1. \end{array}$$

*Hence, the Wronskian of TQDEs with $Q_{TDE}\left(t_{0}\right)=1$ can be calculated as:*

$$\begin{array}{ccc} Q_{TDE}\left(t\right) & = & e_{\tau }\left(t,1\right)Q_{TDE}\left(1\right)=1+\sum_{n=1}^{+\infty }\int_{1}^{t}\tau \left(t_{n}\right)\int_{1}^{t_{n}}\tau \left(t_{n-1}\right)\ldots \int_{1}^{t_{2}}\tau \left(t_{1}\right)\Delta t_{1}\ldots \Delta t_{n-1}\Delta t_{n}\\ & = & 1+\sum_{n=1}^{t}\int_{1}^{t}\tau \left(t_{n}\right)\int_{1}^{t_{n}}\tau \left(t_{n-1}\right)\ldots \int_{1}^{t_{2}}\tau \left(t_{1}\right)\Delta t_{1}\ldots \Delta t_{n-1}\Delta t_{n}\\ & = & 1+\tau (t-1)+\tau (t-2)+\ldots +\tau (1)+\tau (t-1)[\tau (t-2)+\ldots +\tau (1)]\\ & & +\tau (t-2)[\tau (t-3)+\ldots +\tau (1)]+\ldots +\tau (t-1)\tau (t-2)\ldots \tau (1)\\ & = & \left[1+\tau \left(t-1\right)\right]\left[1+\tau \left(t-2\right)\right]\ldots \left[1+\tau \left(1\right)\right]={\left[15\times \left(t-1\right)!\right]}^{t-1}. \end{array}$$

*On the other hand, τ(t)=4t−4 and $Q_{TDE}\left(t\right)=e^{2t^{2}-4t-6}Q_{TDE}\left(1\right)$ for T=R.*

## 5. The Coupled-Jumping Theory on Time Scales

In 2020, Wang, Li, Agarwal, and O’Regan proposed the coupled-jumping theory. It is an interesting topic and can include the Hilger theory and can be used to solve the problems on more general hybrid time scales (see [62,63]).

### 5.1. Vertical Evolution of Time Scales

**Figure 13:**
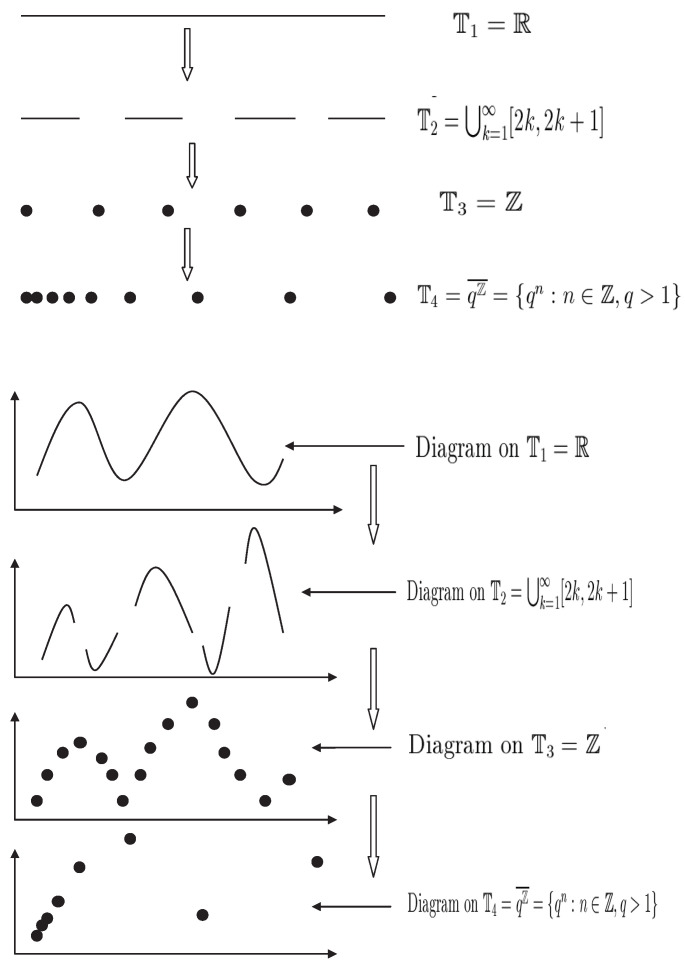
The vertical evolution diagram of dynamical behavior from $T_{1}$ to $T_{4}$ under Hilger theory.

In Figure 13, let $\left\{T_{1},T_{2},T_{3},T_{4}\right\}$ be a timescale group. By Hilger theory, this time scale group will induce a continuous dynamic equation, a piecewise continuous dynamic equation, a discrete dynamic equation, and a quantum dynamic equation in sequence. Starting with the evolution process of these time scales, T varies from the form $T_{1}$ to the form $T_{4}$ in the timescale group, such a vertical evolution in the timescale group acts as a direct factor which leads to the four different types of dynamic equations during the changing process of the time scale T. Only when T is fixed in this timescale group can the concrete dynamic equation be determined. From the viewpoint of the evolution process of time scales, the essence of Hilger’s theory depends on the vertical evolution of time scales; accordingly, the unification of various types of dynamic equation can be achieved when the form of T is fixed in a timescale group. In other words, the related analysis and applications on Hilger theory are purely based on a single time scale during this evolution.

### 5.2. Hybrid-Timescale Problems—A Horizontal Evolution of Time Scales

**Figure 14:**
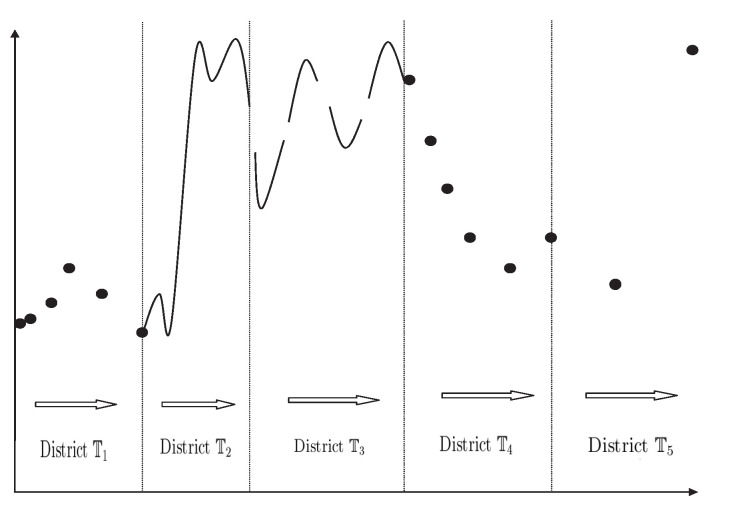
The horizontal evolution diagram of dynamical behavior from $T_{1}$ to $T_{4}$ under coupled-jumping timescale theory.

The other natural and significant evolution of time scales that must be referred to is **horizontal evolution** of time scales. The related problems caused by horizontal evolution of time scales cannot be solved by Hilger theory and they still belong to the problems of timescale category. In Figure 14, let

$$T_{1}=\bar{\left\{q^{n}:q>1,n\in Z^{-}\cup \left\{0\right\}\right\}},\;T_{2}=\left[1.1,3.7\right],\;T_{3}=\overset{5}{\underset{k=2}{⋃}}\left[2k,2k+1\right],$$

$$T_{4}=\left\{12.1,13.1,14.1,15.1,16.1\right\},\;T_{5}=\bar{\left\{{\left(1.5\right)}^{n}:n\geq 7\right\}},\ldots .$$

For convenience, let a timescale group be formed by $\left\{T_{1},T_{2},T_{3},T_{4},T_{5},\ldots \right\}$. It is easy to observe that the dynamical behavior described by Figure 14 exists on the time scale T formed by five districts, and each district is a time scale, i.e., $T=T_{1}\cup T_{2}\cup T_{3}\cup T_{4}\cup T_{5}\cup \ldots$. Therefore, the switch of the dynamical behavior in four timescale districts is directly caused by a **horizontal evolution** of all the time scales in this timescale group.

Usually, all the similar problems described by Figure 14 are called the **hybrid-timescale problems**. Essentially, the hybrid-timescale problems are formed by the problems on multiple time scales, and this class of problems can be precisely depicted by a **horizontal evolution** of time scales in a timescale group.

**Figure 15:**
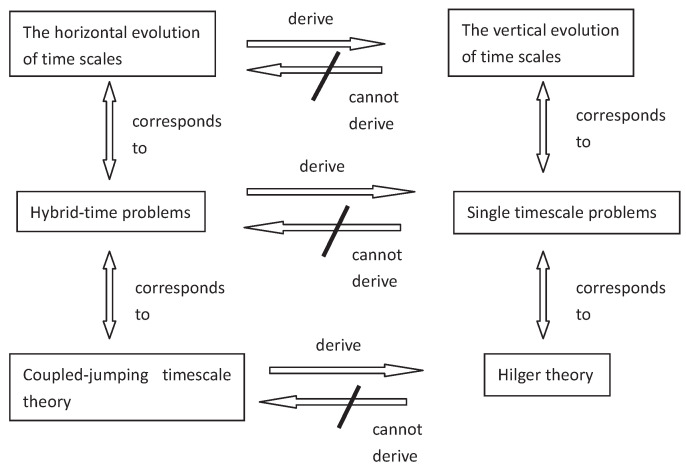
The relation among hybrid-timescale problems, single-timescale problems, Hilger theory and coupled-jumping timescale theory.

By comparison, the related hybrid-timescale problems are more comprehensive and will strictly include the problems on a single time scale as their particular cases (see Figure 15 for their detailed relations). Moreover, the dynamical behavior on hybrid time scales cannot be effectively studied purely on a single time scale through Hilger theory. Therefore, it is very necessary to establish a theory (we call it coupled-jumping timescale theory) to solve the hybrid-timescale problems.

### 5.3. The Description of the Hybrid-Timescale Initial-Value Problems

For understanding the idea to solve the hybrid-timescale problems, we will adopt Figure 14 to illustrate our methods and the framework of the solving steps. Let a timescale group be $\left\{T_{1},T_{2},T_{3},T_{4},T_{5},\ldots \right\}$. To break through the limitation of the Hilger theory and to establish a coupled-jumping timescale theory, demonstrating a distinct dynamical behavior on time scales, firstly, we must consider the formation process of the dynamical behavior in Figure 14. Assume that the dynamical behavior in Figure 14 corresponds to a solution x(t) of a dynamic equation on the hybrid time scales with the initial point $\left(t_{0},x\left(t_{0}\right)\right)$, where $t_{0}=0\in T_{1}$. According to the continuous dependence on initial values of solutions and the continuation theorem, there is a solution on the district $T_{1}$ such that $\left(t_{1},x\left(t_{1}\right)\right)$ is the right boundary point on the district $T_{1}$, where $t_{1}=1\notin T_{2}$. Now taking $\left(t_{1},x\left(t_{1}\right)\right)$ as the initial point, there is a solution on the district $T_{2}$ such that $\left(t_{2},x\left(t_{2}\right)\right)$ is the right boundary point on the district $T_{2}$, where $t_{2}=3.7\notin T_{3}$. Next, by taking $\left(t_{2},x\left(t_{2}\right)\right)$ as the initial point, there is a solution on the district $T_{3}$ such that $\left(t_{3},x\left(t_{3}\right)\right)$ is the right boundary point on the district $T_{3}$, where $t_{3}=11\notin T_{4}$. Repeating the process, by taking $\left(t_{3},x\left(t_{3}\right)\right)$ as the initial point, there is a solution on the district $T_{4}$ such that $\left(t_{4},x\left(t_{4}\right)\right)$ is the right boundary point on the district $T_{4}$, where $t_{4}=16.1\notin T_{5}$. Finally, the solution on the district $T_{5}$ is determined by the initial point $\left(t_{4},x\left(t_{4}\right)\right)$. If there are more time scales after $T_{5}$, for instance, $T_{6},T_{7},\ldots$, the process above can be continued until the solution exists on $T_{1}\cup T_{2}\cup T_{3}\ldots :={⋃}_{i=1}^{+\infty }T_{i}$.

In the above process, a key problem appears. Note that $t_{1}\notin T_{2}$, but the solution on district $T_{2}$ is continuously dependent on $\left(t_{1},x\left(t_{1}\right)\right)$; similarly, $t_{2}\notin T_{3}$, but the solution on district $T_{3}$ is continuously dependent on $\left(t_{2},x\left(t_{2}\right)\right)$,…, $t_{4}\notin T_{5}$, but the solution on district $T_{5}$ is continuously dependent on $(t_{4},x\left(t_{4}\right)),\ldots$. Therefore, the first problem we must solve is that we should introduce an initial value problem of a dynamic equations whose initial value is given in one time scale and the unique solution is located in another. In Ref. [62], the coupled-jumping timescale theory (or hybrid-timescale theory) was proposed.

### 5.4. The Coupled-Jumping Timescale Space (CJTS) and Calculus

A notion of coupled-jumping timescale space and a concept of the hybrid-composition integral was introduced.

**Definition** **71** ([62])**.** *For $\hat{t}\in T_{k}$, we define the forward jump operator $\sigma_{k}:T_{k}\to T_{k}$ by $\sigma_{k}\left(\hat{t}\right)=\inf\left\{s\in T_{k}:s>\hat{t}\right\};$ the backward jump operator $\rho_{k}:T_{k}\to T_{k}$ by $\rho_{k}\left(\hat{t}\right)=\sup\left\{s\in T_{k}:s<\hat{t}\right\}$; and the graininess function $\mu_{k}:T_{k}\to \left[0,+\infty \right)$ by $\mu_{k}\left(\hat{t}\right)=\sigma_{k}\left(\hat{t}\right)-\hat{t}$, where k=1,2.*

The jumping construction of the coupled-jumping timescale space $T_{1}-T_{2}$ was defined.

**Figure 16:**
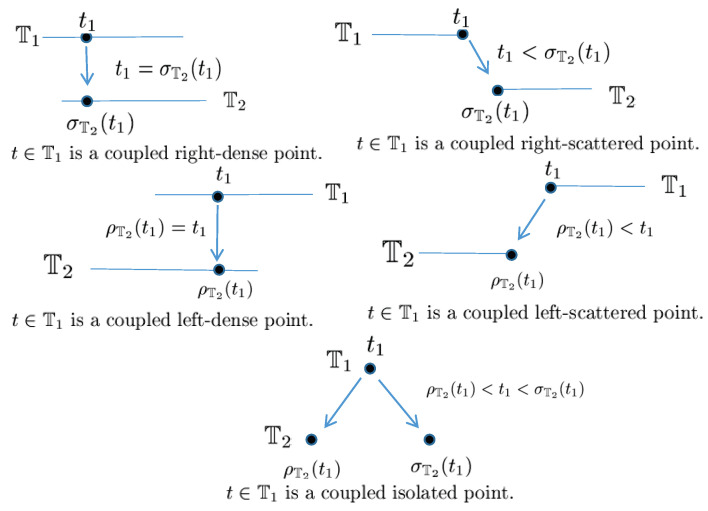
Schematic diagram of all types of coupled-jumping points.

**Definition** **72** ([62])**.** *Let $T_{1}$ and $T_{2}$ be a pair of time scales. For $t\in T_{1}\cup T_{2}$, we define the coupled-forward jump operator between $T_{1}$ and $T_{2}$ by $\sigma_{T_{2}}\left(t\right)=\inf\left\{s\in T_{2}:s\geq t\right\}$, and define the coupled-backward jump operator between $T_{1}$ and $T_{2}$ by $\rho_{T_{2}}\left(t\right)=\sup\left\{s\in T_{2}:s\leq t\right\}$. We say t is a coupled right-dense point iff $\sigma_{T_{2}}\left(t\right)=t$; t is a coupled right-scattered point iff $\sigma_{T_{2}}\left(t\right)>t$; t is a coupled left-dense point iff $\rho_{T_{2}}\left(t\right)=t$; t is a coupled left-scattered point iff $\rho_{T_{2}}\left(t\right)<t$; t is a coupled isolated point iff $\rho_{T_{2}}\left(t\right)<t<\sigma_{T_{2}}\left(t\right)$ (see Figure 16).*

**Figure 17:**
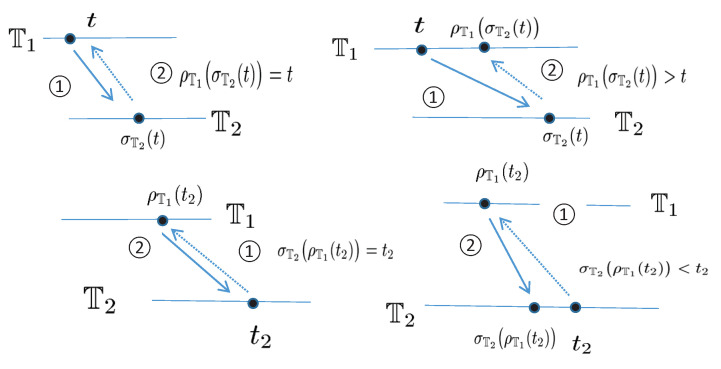
The jump of coupled-jumping points in Remark 10.

**Remark** **10.** *In Definition 72, one can obtain $\sigma_{T_{2}}\left(t_{2}\right)=\rho_{T_{2}}\left(t_{2}\right)=t_{2}$ for $t_{2}\in T_{2}$; $\rho_{T_{1}}\left(\sigma_{T_{2}}\left(t\right)\right)\geq t$ for $t\in T_{1}$; $\sigma_{T_{2}}\left(\rho_{T_{1}}\left(t_{2}\right)\right)\leq t_{2}$ for $t_{2}\in T_{2}$. Note that $\rho_{T_{1}}\left(\sigma_{T_{2}}\left(t\right)\right)=t$ if and only if $\left(t,\sigma_{T_{2}}\left(t\right)\right)\cap T_{1}=\emptyset$; $\sigma_{T_{2}}\left(\rho_{T_{1}}\left(t_{2}\right)\right)=t_{2}$ if and only if $\left(\rho_{T_{1}}\left(t_{2}\right),t_{2}\right)\cap T_{2}=\emptyset$, where *∅* is an empty set (see Figure 17).*

**Definition** **73** ([62])**.** *Let $T_{1}$ and $T_{2}$ be a pair of time scales. We define $T_{k}^{\overset{´}{\kappa }}$ and $T_{k}^{\overset{‘}{\kappa }}$ as follows:*

$$\begin{array}{ccc} T_{k}^{\overset{´}{\kappa }} & = & \left\{\begin{array}{c} T_{k}\setminus \left(\sup T_{j},+\infty \right)\;\;\;if\;\;\sup T_{j}\;is\;a\;finite\;number,\\ T_{k}\;\;\;otherwise, \end{array}\right.\\ T_{k}^{\overset{‘}{\kappa }} & = & \left\{\begin{array}{c} T_{k}\setminus \left(-\infty ,\inf T_{j}\right)\;\;\;if\;\;\inf T_{j}\;is\;a\;finite\;number,\\ T_{k}\;\;\;otherwise, \end{array}\right. \end{array}$$

*where k,j∈{1,2} and k≠j.*

**Definition** **74** ([62])**.** *Let $T_{1}$ and $T_{2}$ be a pair of time scales. We define $T_{k}^{\bar{\kappa }}$ as follows:*

$$T_{k}^{\bar{\kappa }}=\left\{\begin{array}{c} T_{k}\setminus \left(-\infty ,\inf T_{j}\right)\cup \left(\sup T_{j},+\infty \right)\;\;\;if\;\;\inf T_{j},\sup T_{j}\;are\;finite\;numbers,\\ T_{k}\setminus \left(-\infty ,\inf T_{j}\right)\;\;\;if\;\;\inf T_{j}\;is\;a\;finite\;number,\;\sup T_{j}=+\infty ,\\ T_{k}\setminus \left(\sup T_{j},+\infty \right)\;\;\;if\;\;\sup T_{j}\;is\;a\;finite\;number,\;\inf T_{j}=-\infty ,\\ T_{k}\;\;\;otherwise, \end{array}\right.$$

*where k,j∈{1,2} and k≠j.*

**Remark** **11.** *In Definition 74, if $T_{1}=T_{2}=T$, then $T^{\bar{\kappa }}=T$ and a Hilger time scale is obtained.*

**Remark** **12.** *In Definitions 73 and 74, we obtain that $T_{k}^{\bar{\kappa }}=T_{k}^{\overset{´}{\kappa }}\cap T_{k}^{\overset{‘}{\kappa }}$.*

**Figure 18:**
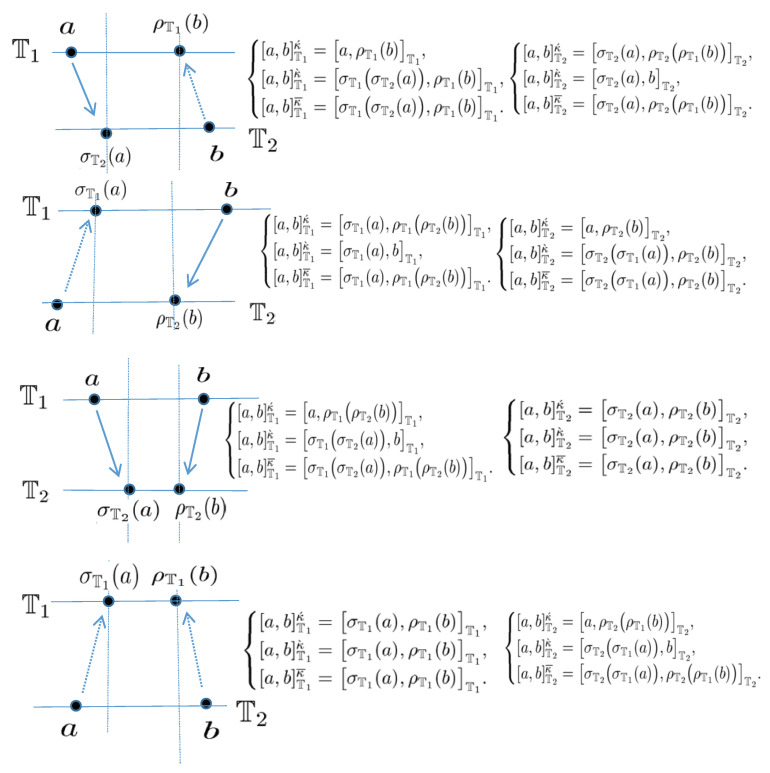
The jump of coupled-jumping points in Remark 13.

**Remark** **13.** *Note that $a,b\in T_{1}\cup T_{2}$ and $\left[a,b\right]\cap T_{j}\neq \emptyset$, for a<b and j=1,2, one can obtain $\left[a,b\right]\cap T_{1}={\left[\sigma_{T_{1}}\left(a\right),\rho_{T_{1}}\left(b\right)\right]}_{T_{1}}$ and $\left[a,b\right]\cap T_{2}={\left[\sigma_{T_{2}}\left(a\right),\rho_{T_{2}}\left(b\right)\right]}_{T_{2}}$. Let $\tilde{a}=\max\left\{\sigma_{T_{1}}\left(a\right),\sigma_{T_{2}}\left(a\right)\right\}$ and $\tilde{b}=\min\left\{\rho_{T_{1}}\left(b\right),\rho_{T_{2}}\left(b\right)\right\}$. Then, ${\left[\sigma_{T_{j}}\left(a\right),\rho_{T_{j}}\left(b\right)\right]}_{T_{j}}^{\overset{´}{\kappa }}=[\sigma_{T_{j}}\left(a\right),$$\rho_{T_{j}}\left(\tilde{b}\right){]}_{T_{j}}$, ${\left[\sigma_{T_{j}}\left(a\right),\rho_{T_{j}}\left(b\right)\right]}_{T_{j}}^{\overset{‘}{\kappa }}={\left[\sigma_{T_{j}}\left(\tilde{a}\right),\rho_{T_{j}}\left(b\right)\right]}_{T_{j}}$, ${\left[\sigma_{T_{j}}\left(a\right),\rho_{T_{j}}\left(b\right)\right]}_{T_{j}}^{\bar{\kappa }}={\left[\sigma_{T_{j}}\left(\tilde{a}\right),\rho_{T_{j}}\left(\tilde{b}\right)\right]}_{T_{j}}$, where j∈{1,2} (see Figure 18). Notice that, for any $\hat{a},\hat{b}\in T_{j}$, the intervals ${\left[\hat{a},\hat{b}\right)}_{T_{j}},\;{\left(\hat{a},\hat{b}\right)}_{T_{j}}$ with $\hat{a}\geq \hat{b}$ are always regarded as the empty sets. According to the *Δ*-measure theory on time scales [25], it is well-known that the *Δ*-integral of a function f(t) equals to zero on the empty set since $\mu_{\Delta }\left(\emptyset \right)=0$.*

**Theorem** **74** ([62])**.** *Let $t_{1}\in T_{1}^{\overset{‘}{\kappa }}$. If $\rho_{T_{2}}\left(\sigma_{1}\left(t_{1}\right)\right)=\sigma_{2}\left(\rho_{T_{2}}\left(t_{1}\right)\right)$ and $\mu_{1}\left(t_{1}\right)=\mu_{2}\left(\rho_{T_{2}}\left(t_{1}\right)\right)$, then $\rho_{T_{2}}\left(t_{1}\right)\leq t_{1}\leq \rho_{T_{2}}\left(\sigma_{1}\left(t_{1}\right)\right)\leq \sigma_{1}\left(t_{1}\right)$.*

**Remark** **14.** *In Theorem 74, if $t_{1}\in T_{1}\cap T_{2}$, then $\rho_{T_{2}}\left(t_{1}\right)=t_{1}$ and $\rho_{T_{2}}\left(\sigma_{1}\left(t_{1}\right)\right)=\sigma_{1}\left(t_{1}\right)$.*

**Figure 19:**
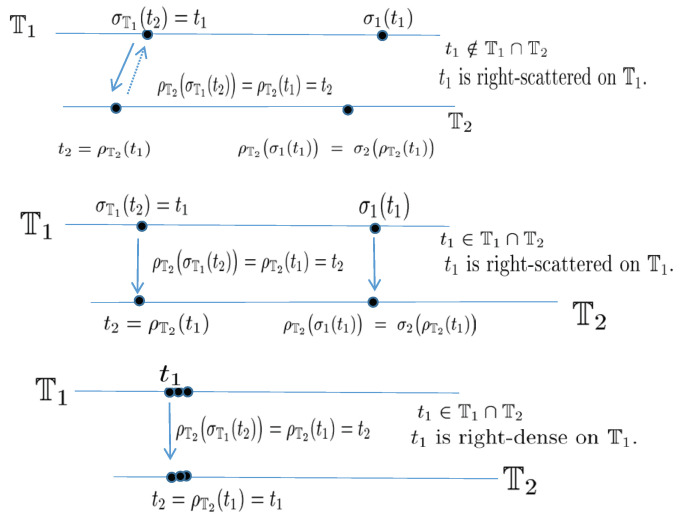
The jump situation for the coupled-jumping points in Theorem 75.

**Theorem** **75** ([62])**.** *Assume $\rho_{T_{2}}\left(\sigma_{1}\left(t_{1}\right)\right)=\sigma_{2}\left(\rho_{T_{2}}\left(t_{1}\right)\right)$ and $\mu_{1}\left(t_{1}\right)=\mu_{2}\left(\rho_{T_{2}}\left(t_{1}\right)\right)$ for any $t_{1}\in T_{1}^{\overset{‘}{\kappa }}$. Then, $\rho_{T_{2}}\left(\sigma_{T_{1}}\left(t_{2}\right)\right)=t_{2}$ for any $t_{2}\in T_{2}^{\overset{´}{\kappa }}$ (see Figure 19).*

**Definition** **75** ([62])**.** *Let $f:T_{1}\cup T_{2}\to R$. We define a hybrid-composition integral (or short for HC-integral) of f(t) on CJTS as follows:*

$$\int_{a}^{b}f\left(\tau \right)\Delta_{m}\tau =\left\{\begin{array}{c} \alpha \int_{{\left[\sigma_{T_{1}}\left(a\right),\rho_{T_{1}}\left(b\right)\right]}_{T_{1}}}f\left(\tau \right)\Delta_{1}\tau +\left(1-\alpha \right)\int_{{\left[\sigma_{T_{2}}\left(a\right),\rho_{T_{2}}\left(b\right)\right]}_{T_{2}}}f\left(\tau \right)\Delta_{2}\tau ,\;\;\;a<b,\\ -\alpha \int_{{\left[\sigma_{T_{1}}\left(b\right),\rho_{T_{1}}\left(a\right)\right]}_{T_{1}}}f\left(\tau \right)\Delta_{1}\tau -\left(1-\alpha \right)\int_{{\left[\sigma_{T_{2}}\left(b\right),\rho_{T_{2}}\left(a\right)\right]}_{T_{2}}}f\left(\tau \right)\Delta_{2}\tau ,\;\;\;a>b, \end{array}\right.$$

*where $a,b\in T_{1}\cup T_{2}$, 0≤α≤1 and α is called the hybrid-composition proportion coefficient.*

**Theorem** **76** ([62])**.** *If $a,b,c\in T_{1}\cup T_{2}$, $\tilde{\alpha }\in R$, $f,g:T_{1}\cup T_{2}\to R$, then*

(*i*) *Let $\left[a_{k},a_{k+1}\right]\cap T_{l}\neq \emptyset$, k,l∈{1,2} and $\left\{a,b,c\right\}=\left\{a_{j}|j=1,2,3,a_{1}<a_{2}<a_{3}\right\}$. Then, $\int_{a}^{b}f\left(\tau \right)\Delta_{m}\tau =\int_{a}^{c}f\left(\tau \right)\Delta_{m}\tau +\int_{c}^{b}f\left(\tau \right)\Delta_{m}\tau$ if $a_{2}\in T_{1}\cap T_{2}$; $\int_{a}^{b}f\left(\tau \right)\Delta_{m}\tau \neq \int_{a}^{c}f\left(\tau \right)\Delta_{m}\tau +\int_{c}^{b}f\left(\tau \right)\Delta_{m}\tau$ if $a_{2}\notin T_{1}\cap T_{2}$;*
(*ii*) *$\int_{a}^{b}\left(f(\tau )+g(\tau )\right)\Delta_{m}\tau =\int_{a}^{b}f\left(\tau \right)\Delta_{m}\tau +\int_{a}^{b}g\left(\tau \right)\Delta_{m}\tau$;*
(*iii*) *$\int_{a}^{b}\tilde{\alpha }f\left(\tau \right)\Delta_{m}\tau =\tilde{\alpha }\int_{a}^{b}f\left(\tau \right)\Delta_{m}\tau$;*
(*iv*) *$\int_{a}^{b}f\left(\tau \right)\Delta_{m}\tau =-\int_{b}^{a}f\left(\tau \right)\Delta_{m}\tau$;*
(*v*) *$\int_{a}^{a}f\left(\tau \right)\Delta_{m}\tau =0$;*
(*vi*) *$\int_{a}^{b}f\left(\tau \right)\Delta_{m}\tau \geq 0$ if f≥0 for all a≤τ<b.*

In the following, we introduce the exponential function on coupled-jumping time scales and describe the basic theory of time-hybrid dynamic equations.

**Definition** **76** ([62])**.** *Let $\overset{ˇ}{t},s\in T_{1}\cup T_{2}$. We introduce the HC-exponential function by*

$$\begin{array}{ccc} & & {\bar{e}}_{f}\left(\overset{ˇ}{t},s\right):=\\ & & \left\{\begin{array}{c} \exp\left\{\alpha \int_{{\left[\sigma_{T_{1}}\left(s\right),\rho_{T_{1}}\left(\overset{ˇ}{t}\right)\right]}_{T_{1}}}\frac{\mathrm{Log}(1+\mu_{1}\left(\tau \right)f\left(\tau \right))}{\mu_{1}\left(\tau \right)}\Delta_{1}\tau +\left(1-\alpha \right)\int_{{\left[\sigma_{T_{2}}\left(s\right),\rho_{T_{2}}\left(\overset{ˇ}{t}\right)\right]}_{T_{2}}}\frac{\mathrm{Log}(1+\mu_{2}\left(\tau \right)f\left(\tau \right))}{\mu_{2}\left(\tau \right)}\Delta_{2}\tau \right\}\\ \;\;\;s<\overset{ˇ}{t},\\ \exp\left\{-\alpha \int_{{\left[\sigma_{T_{1}}\left(\overset{ˇ}{t}\right),\rho_{T_{1}}\left(s\right)\right]}_{T_{1}}}\frac{\mathrm{Log}(1+\mu_{1}\left(\tau \right)f\left(\tau \right))}{\mu_{1}\left(\tau \right)}\Delta_{1}\tau -\left(1-\alpha \right)\int_{{\left[\sigma_{T_{2}}\left(\overset{ˇ}{t}\right),\rho_{T_{2}}\left(s\right)\right]}_{T_{2}}}\frac{\mathrm{Log}(1+\mu_{2}\left(\tau \right)f\left(\tau \right))}{\mu_{2}\left(\tau \right)}\Delta_{2}\tau \right\}\\ \;\;\;s>\overset{ˇ}{t}. \end{array}\right. \end{array}$$

Next, we demonstrate the HC-exponential solution of the homogeneous time-hybrid dynamic equation.

**Theorem** **77** ([62])**.** *Let $t\in T_{1}^{\bar{\kappa }}$, $s\in T_{2}^{\bar{\kappa }}$, t≥s. Then, ${\bar{e}}_{f}\left(t,s\right)$ is the solution of the initial value problem*

(42) $$\mu_{1}\left(t\right)x^{\Delta_{t}}\left(t\right)=\left\{{\left(1+\mu_{1}\left(t\right)f\left(t\right)\right)}^{\alpha }\exp\left\{\left(1-\alpha \right)\int_{\rho_{T_{2}}\left(t\right)}^{\rho_{T_{2}}\left(\sigma_{1}\left(t\right)\right)}\frac{\mathrm{Log}(1+\mu_{2}\left(\tau \right)f\left(\tau \right))}{\mu_{2}\left(\tau \right)}\Delta_{2}\tau \right\}-1\right\}x\left(t\right),$$

*with the initial value x(s)=1, where $x^{\Delta_{t}}\left(t\right)$ denotes the *Δ*-derivative at t on $T_{1}$.*

The theorem below is the existence and uniqueness theorem of the HC-exponential solution to the homogeneous time-hybrid dynamic equation on CJTS.

**Theorem** **78** (Existence and Uniqueness of Solutions, [62])**.** *For the initial value problem of *(42)*, there exists a unique solution $x\left(t\right)=x_{0}{\bar{e}}_{f}\left(t,s\right)$.*

Based on the theory, the time-hybrid dynamic equations, convolution, and Laplace transforms were proposed and studied in [62] in detail.

## 6. Combined Measure Theory on Time Scales

The measure theory on time scales was considered in [64,65]. The combined theory on time scales was initiated in [66], and it was widely used in mathematical analysis. In [67], the authors obtained the non-eigenvalue form of Liouville’s formula and α-matrix exponential solutions for combined matrix dynamic equations on time scales. In 2020, Wang, Qin, Agarwal, and O’Regan (see [68]) established the ${⋄}_{\alpha }$-measurability and combined measure theory on time scales.

### 6.1. ${⋄}_{\alpha }$-Measurability and ${⋄}_{\alpha }$-Measure

**Definition** **77** ([68])**.** *Let T be a time scale, σ and ρ be the forward and back jumping operators, and a combined interval (or α-interval) be*

$${\left[a,b\right]}^{\alpha }:=\left\{\begin{array}{cc} (a,b]\cap T,\;\; & \alpha =0,\\ (a,b)\cap T,\; & 0<\alpha <1,\\ {}[a,b)\cap T,\; & \alpha =1, \end{array}\right.$$

*where (a,b]∩T={t∈T:a<t⩽b,a,b∈T}, (a,b)∩T={t∈T:a<t<b,a,b∈T}, [a,b)∩T={t∈T:a⩽t<b,a,b∈T}. Let K be the family of all combined intervals.*

*Then, we present the set function $m_{{⋄}_{\alpha }}$ corresponding to ${\left[a,b\right]}^{\alpha }$ as*

$$m_{{⋄}_{\alpha }}\left({\left[a,b\right]}^{\alpha }\right)=\left\{\begin{array}{cc} b-a, & \alpha =0,\\ \alpha (b-\sigma (a))+(1-\alpha )(\rho (b)-a), & \alpha \in (0,1),\\ b-a, & \alpha =1. \end{array}\right.$$

*For a=b, we appoint that ${\left[a,b\right]}^{\alpha }=⌀$, and $m_{{⋄}_{\alpha }}\left({\left[a,b\right]}^{\alpha }\right)=0$.*

**Definition** **78** ([68])**.** *Let E⊂T. If there exists at least one finite or countable system of intervals ${\left[a_{n},b_{n}\right]}^{\alpha }\in K\left(n=1,2,...\right)$ such that $E\subset \underset{n\in N_{0}}{⋃}{\left[a_{n},b_{n}\right]}^{\alpha }$, then we call $m_{{⋄}_{\alpha }}^{*}\left(E\right)=\inf\sum_{n\in N_{0}}m_{{⋄}_{\alpha }}\left({\left[a_{n},b_{n}\right]}^{\alpha }\right)$ the outer ${⋄}_{\alpha }$-measure of E, where the infimum is taken over all coverings of E by a finite or countable system of intervals ${\left[a_{n},b_{n}\right]}^{\alpha }\in K$. If there is no such covering of E, we say $m_{{⋄}_{\alpha }}^{*}\left(E\right)=\infty$.*

**Definition** **79** ([68])**.** *We say a property that holds everywhere except for a null set is ${⋄}_{\alpha }$-almost everywhere, briefly ${⋄}_{\alpha }$-a.e. in combined measure theory on time scales.*

**Theorem** **79** ([68])**.** *Let A⊂T, B⊂T and $m_{{⋄}_{\alpha }}^{*}\left(A\right)$, $m_{{⋄}_{\alpha }}^{*}\left(B\right)$ be the outer ${⋄}_{\alpha }$-measure of A and B, respectively. Then,*

(*1*) *$m_{{⋄}_{\alpha }}^{*}\left(A\right)⩾0$, if A=⌀, then $m_{{⋄}_{\alpha }}^{*}\left(E\right)=0$;*
(*2*) *let A⊂B, then $m_{{⋄}_{\alpha }}^{*}\left(A\right)⩽m_{{⋄}_{\alpha }}^{*}\left(B\right)$;*
(*3*) $m_{{⋄}_{\alpha }}^{*}\left(\overset{\infty }{\underset{i=1}{⋃}}A_{i}\right)⩽\sum_{i=1}^{\infty }m_{{⋄}_{\alpha }}^{*}\left(A_{i}\right).$

**Definition** **80** ([68])**.** *A set E⊂T is called ${⋄}_{\alpha }$-measurable (or $m_{{⋄}_{\alpha }}^{*}$-measurable) if*

$$m_{{⋄}_{\alpha }}^{*}\left(P^{\alpha }\right)=m_{{⋄}_{\alpha }}^{*}\left(P^{\alpha }\cap E\right)+m_{{⋄}_{\alpha }}^{*}\left(P^{\alpha }\cap E^{c}\right)$$

*holds for all $P^{\alpha }\in K$, where $E^{c}=T-E$. We let $N(m_{{⋄}_{\alpha }}^{*})$ be the family of all $m_{{⋄}_{\alpha }}^{*}$-measurable sets as*

$$N\left(m_{{⋄}_{\alpha }}^{*}\right)=\left\{E\subset T:E\;\;\mathrm{is}\;\;m_{{⋄}_{\alpha }}^{*}-measurable\right\}.$$

The following sufficient and necessary condition for ${⋄}_{\alpha }$-measurability can be established.

**Theorem** **80** ([68])**.** *Letting E⊂T is ${⋄}_{\alpha }$-measurable if and only if for any $A\subset E,B\subset E^{c}$, we have*

$$m_{{⋄}_{\alpha }}^{*}\left(A\cup B\right)=m_{{⋄}_{\alpha }}^{*}\left(A\right)+m_{{⋄}_{\alpha }}^{*}\left(B\right).$$

**Theorem** **81** ([68])**.** *Let $\left\{E_{i}\right\}$ be a sequence pairwise disjoint ${⋄}_{\alpha }$-measurable sets, then $\overset{\infty }{\underset{i=1}{⋃}}E_{i}$ is ${⋄}_{\alpha }$-measurable, and*

$$m_{{⋄}_{\alpha }}^{*}\left(\overset{\infty }{\underset{i=1}{⋃}}E_{i}\right)=\sum_{i=1}^{\infty }m_{{⋄}_{\alpha }}^{*}\left(E_{i}\right).$$

Now, the Lebesgue ${⋄}_{\alpha }$-measure denoted by $\mu_{{⋄}_{\alpha }}$ is $m_{{⋄}_{\alpha }}^{*}$ restricted to $N(m_{{⋄}_{\alpha }}^{*})$, and it is a countably additive measure.

**Theorem** **82** ([68])**.** *Let $\left\{E_{i}\right\}$ be an increasing sequence of ${⋄}_{\alpha }$-measurable set in T, such that $E_{1}\subset E_{2}\subset \cdots \subset E_{n}\subset \cdots$, then let $E=\overset{\infty }{\underset{i=1}{⋃}}E_{i}=\lim_{n\to \infty }E_{n}$, and we have*

$$\mu_{{⋄}_{\alpha }}\left(E\right)=\lim_{n\to \infty }\mu_{{⋄}_{\alpha }}\left(E_{n}\right).$$

*If $\left\{E_{n}\right\}$ is a decreasing sequence of ${⋄}_{\alpha }$-measurable set in T such that $E_{1}⊃E_{2}⊃\cdots ⊃E_{n}⊃\cdots$, let $E=\overset{\infty }{\underset{i=1}{⋂}}E_{i}=\lim_{n\to \infty }E_{n}$, then, when $\mu_{{⋄}_{\alpha }}\left(E_{1}\right)<\infty$, we have*

$$\mu_{{⋄}_{\alpha }}\left(E\right)=\lim_{n\to \infty }\mu_{{⋄}_{\alpha }}\left(E_{n}\right).$$

Some basic theorems and lemmas were obtained.

**Theorem** **83** ([68])**.** *If a,b∈T−{minT,maxT} and a<b, then*

(*i*) $\mu_{{⋄}_{\alpha }}\left(\left(a,b\right)\right)=\alpha \left(b-\sigma \left(a\right)\right)+\left(1-\alpha \right)\left(\rho \left(b\right)-a\right).$
(*ii*) $\mu_{{⋄}_{\alpha }}\left(\left(a,b\right]\right)=\alpha \left(\sigma \left(b\right)-\sigma \left(a\right)\right)+\left(1-\alpha \right)\left(b-a\right).$
(*iii*) $\mu_{{⋄}_{\alpha }}\left(\left[a,b\right)\right)=\alpha \left(b-a\right)+\left(1-\alpha \right)\left(\rho \left(b\right)-\rho \left(a\right)\right).$
(*iv*) $\mu_{{⋄}_{\alpha }}\left(\left[a,b\right]\right)=\alpha \left(\sigma \left(b\right)-a\right)+\left(1-\alpha \right)\left(b-\rho \left(a\right)\right).$

**Remark** **15.** *Notice that $\mu_{{⋄}_{\alpha }}=\mu_{\nabla }$ when α=0, and $\mu_{{⋄}_{\alpha }}=\mu_{\Delta }$ when α=1, and if α∈(0,1), $\mu_{{⋄}_{\alpha }}$ is a linear combination of $\mu_{\nabla }$ and $\mu_{\Delta }$. Thus, for any interval E⊂T, we can conclude as follows:*

$$\mu_{{⋄}_{\alpha }}\left(E\right)=\alpha \mu_{\Delta }\left(E\right)+\left(1-\alpha \right)\mu_{\nabla }\left(E\right),\;\;\alpha \in \left[0,1\right].$$

### 6.2. Lebesgue Measurable and Lebesgue ${⋄}_{\alpha }$-Measurable Sets

In this subsection, we denote the usual Lebesgue measure on R by *L* and the corresponding outer measure by $L^{*}$, i.e.,

$$L^{*}\left(E\right)=\inf\left\{\sum_{j\in J}\left(\beta_{j}-\alpha_{j}\right):E\subset \underset{j\in J}{⋃}\left(\alpha_{j},\beta_{j}\right),\alpha_{j},\beta_{j}\in R,\alpha_{j}⩽\beta_{j},J\in N_{0}\right\}.$$

From the above, we can easily see that the set of all left-scattered points of T is also countable; then, the set of all isolate points is countable. For the convenience, we define the following sets:

(43) $$\begin{array}{c} \begin{array}{cc} \; & A:=\{t\in T:t\;\;\mathrm{is}\;\mathrm{left}-\mathrm{dense}\;\mathrm{and}\;\mathrm{right}-\mathrm{scattered}\},\\ \; & B:=\{t\in T:t\;\;\mathrm{is}\;\mathrm{left}-\mathrm{scattered}\;\mathrm{and}\;\mathrm{right}-\mathrm{dense}\},\\ \; & C:=\{t\in T:t\;\;\mathrm{is}\;\mathrm{left}-\mathrm{scattered}\;\mathrm{and}\;\mathrm{right}-\mathrm{scattered}\},\\ \; & D:=\{t\in T:t\;\;\mathrm{is}\;\mathrm{left}-\mathrm{dense}\;\mathrm{and}\;\mathrm{right}-\mathrm{dense}\}. \end{array} \end{array}$$

**Theorem** **84** ([68])**.** *If E⊂T−{maxT,minT}, then the following properties are satisfied:*

(a) $L^{*}\left(E\right)⩽m_{{⋄}_{\alpha }}^{*}\left(E\right).$
(b) *If E has no scattered points, then $L^{*}\left(E\right)=m_{{⋄}_{\alpha }}^{*}\left(E\right).$*
(c) *The sets A,B,C,D defined in *(43)* are Lebesgue measurable. Moreover $L^{*}\left(A\right)=L^{*}\left(B\right)=L^{*}\left(C\right)=0$. In addition,*
  $$\begin{array}{c} \begin{array}{c} \mu_{{⋄}_{\alpha }}\left(E\cap A\right)=\alpha \sum_{i\in I_{E\cap A}}\left(\sigma \left(t_{i}\right)-t_{i}\right),\;\;\mu_{{⋄}_{\alpha }}\left(E\cap B\right)=\left(1-\alpha \right)\sum_{i\in I_{E\cap B}}\left(t_{i}-\rho \left(t_{i}\right)\right),\\ \mu_{{⋄}_{\alpha }}\left(E\cap C\right)=\alpha \sum_{i\in I_{E\cap C}}\left(\sigma \left(t_{i}\right)-t_{i}\right)+\left(1-\alpha \right)\sum_{i\in I_{E\cap C}}\left(t_{i}-\rho \left(t_{i}\right)\right), \end{array} \end{array}$$
  *where $I_{E\cap A},I_{E\cap B},I_{E\cap C}$ indicates the indices set for all right-scattered and left-dense points, the indices set for all left-scattered and right-dense points, and the indices set for all left-scattered and right-scattered points in E, respectively.*
(d) $m_{{⋄}_{\alpha }}^{*}\left(E\right)=L^{*}\left(E\right)+\alpha \sum_{i\in I_{E\cap (A\cup C)}}\left(\sigma \left(t_{i}\right)-t_{i}\right)+\left(1-\alpha \right)\sum_{i\in I_{E\cap (B\cup C)}}\left(t_{i}-\rho \left(t_{i}\right)\right).$
(e) *$m_{{⋄}_{\alpha }}^{*}\left(E\right)=\mu_{L}^{*}\left(E\right)$ if and only if E has no scattered points.*

**Theorem** **85** ([68])**.** *Let E⊂T, then E is Lebesgue ${⋄}_{\alpha }$-measurable if and only if it is Lebesgue measurable. In such a case, for E⊂T−{maxT,minT}, the following is true:*

(*i*) *$\mu_{{⋄}_{\alpha }}\left(E\right)=L\left(E\right)+\alpha \sum_{i\in I_{E\cap S_{R}}}\left(\sigma \left(t_{i}\right)-t_{i}\right)+\left(1-\alpha \right)\sum_{i\in I_{E\cap S_{L}}}\left(t_{i}-\rho \left(t_{i}\right)\right)$, where $I_{E\cap S_{R}}$ and $I_{E\cap S_{L}}$ denote the index set of all right-scattered points of E and the index set of all left-scattered points of E, respectively.*
(*ii*) *$L\left(E\right)=\mu_{{⋄}_{\alpha }}\left(E\right)$ if and only if maxT∉E,minT∉E and E has no scattered points.*

**Remark** **16.** *Using Theorem 85, we get*

$$\mu_{{⋄}_{\alpha }}\left(E\right)=\alpha L\left(E^{{⋄}_{1}}\right)+\left(1-\alpha \right)L\left(E^{{⋄}_{2}}\right),$$

*where E⊂T−{minT,maxT}, α∈[0,1] and $E^{{⋄}_{1}},E^{{⋄}_{2}}$ are the extension of E. In fact, through direct calculation, we have*

$$\begin{array}{cc} \mu_{{⋄}_{\alpha }}\left(E\right) & =\alpha \sum_{i\in I_{E\cap (A\cup C)}}\left(\sigma \left(t_{i}\right)-t_{i}\right)+\left(1-\alpha \right)\sum_{i\in I_{E\cap (A\cup C)}}\left(t_{i}-\rho \left(t_{i}\right)\right)+L\left(E\right)\\ \; & =\alpha \sum_{i\in I_{E\cap (A\cup C)}}L\left(\left(t_{i},\sigma \left(t_{i}\right)\right)\right)+\left(1-\alpha \right)\sum_{i\in I_{E\cap (A\cup C)}}L\left(\left(\rho \left(t_{i}\right),t_{i}\right)\right)+\alpha L\left(E\right)+\left(1-\alpha \right)L\left(E\right)\\ \; & =\alpha \left(L\left(\underset{i\in I_{E}}{⋃}\left(t_{i},\sigma \left(t_{i}\right)\right)\right)+L\left(E\right)\right)+\left(1-\alpha \right)\left(L\left(\underset{j\in J_{E}}{⋃}\left(\rho \left(t_{j}\right),t_{j}\right)\right)\right)+L\left(E\right)\\ \; & =\alpha L\left(\underset{i\in I_{E}}{⋃}\left(t_{i},\sigma \left(t_{i}\right)\right)\cup E\right)+\left(1-\alpha \right)L\left(\underset{j\in J_{E}}{⋃}\left(\rho \left(t_{i}\right),t_{j}\right)\cup \left(E\right)\right)=\alpha L\left(E^{{⋄}_{1}}\right)+\left(1-\alpha \right)L\left(E^{{⋄}_{2}}\right). \end{array}$$

**Theorem** **86** ([68])**.** *Let E⊂T, then E is Lebesgue ${⋄}_{\alpha }$-measurable if and only if it is Lebesgue measurable. In such a case, for E⊂T−{maxT,minT}, the following is true:*

(*i*) *$\mu_{{⋄}_{\alpha }}\left(E\right)=L\left(E\right)+\alpha \sum_{i\in I_{E\cap S_{R}}}\left(\sigma \left(t_{i}\right)-t_{i}\right)+\left(1-\alpha \right)\sum_{i\in I_{E\cap S_{L}}}\left(t_{i}-\rho \left(t_{i}\right)\right)$, where $I_{E\cap S_{R}}$ and $I_{E\cap S_{L}}$ denote the index set of all right-scattered points of E and the index set of all left-scattered points of E, respectively.*
(*ii*) *$L\left(E\right)=\mu_{{⋄}_{\alpha }}\left(E\right)$ if and only if maxT∉E,minT∉E and E has no scattered points.*

### 6.3. Lebesgue–Stieltjes ${⋄}_{\alpha }$-measurability

**Definition** **81** ([69])**.** *The function $m_{{⋄}_{\alpha }}^{\beta }:J_{T}\to \left[0,+\infty \right)$ is called a pre-measure if the following equalities are satisfied:*

(*i*) *$m_{{⋄}_{\alpha }}^{\beta }\left([a,b)\right)=\alpha \left(\beta \left(b^{-}\right)-\beta \left(a^{-}\right)\right)+\left(1-\alpha \right)\left(\beta \left(\rho {\left(b\right)}^{-}\right)-\beta \left(\rho {\left(a\right)}^{-}\right)\right)$,*
(*ii*) *$m_{{⋄}_{\alpha }}^{\beta }\left([a,b]\right)=\alpha \left(\beta \left(\sigma {\left(b\right)}^{+}\right)-\beta \left(a^{-}\right)\right)+\left(1-\alpha \right)\left(\beta \left(b^{+}\right)-\beta \left(\rho {\left(a\right)}^{-}\right)\right)$,*
(*iii*) *$m_{{⋄}_{\alpha }}^{\beta }\left((a,b]\right)=\alpha \left(\beta \left(\sigma {\left(b\right)}^{+}\right)-\beta \left(\sigma {\left(a\right)}^{+}\right)\right)+\left(1-\alpha \right)\left(\beta \left(b^{+}\right)-\beta \left(a^{+}\right)\right)$,*
(*iv*) *If b>σ(a), $m_{{⋄}_{\alpha }}^{\beta }\left((a,b)\right)=\alpha \left(\beta \left(b^{-}\right)-\beta \left(\sigma {\left(a\right)}^{+}\right)\right)+\left(1-\alpha \right)\left(\beta \left(\rho {\left(b\right)}^{-}\right)-\beta \left(a^{+}\right)\right)$,*

*where α∈[0,1], $J_{T}$ denotes the family of all intervals of T, β:T→R is a monotone increasing function.*

Then, the notion of Lebesgue–Stieltjes ${⋄}_{\alpha }$-outer measure ${\left(m_{{⋄}_{\alpha }}^{\beta }\right)}^{*}$ was introduced as follows.

**Definition** **82** ([69])**.** *The function ${\left(m_{{⋄}_{\alpha }}^{\beta }\right)}^{*}:J_{T}\to \left[0,+\infty \right)$ associated with β defined by*

$${\left(m_{{⋄}_{\alpha }}^{\beta }\right)}^{*}\left(E\right)=\inf\sum_{i=1}^{\infty }m_{{⋄}_{\alpha }}^{\beta }\left(I_{n}\right),$$

*is called a Lebesgue–Stieltjes ${⋄}_{\alpha }$-outer measure of E if there exists at least one finite or countable covering system of intervals $I_{n}\subset J_{T}$ of E satisfies $E\subset {⋃}_{n=1}^{\infty }I_{n}$. We say ${\left(m_{{⋄}_{\alpha }}^{\beta }\right)}^{*}\left(E\right)=\infty$ if there is no such a covering of E. If*

$${\left(m_{{⋄}_{\alpha }}^{\beta }\right)}^{*}\left(A\right)={\left(m_{{⋄}_{\alpha }}^{\beta }\right)}^{*}\left(A\cap E\right)+{\left(m_{{⋄}_{\alpha }}^{\beta }\right)}^{*}\left(A\cap E^{c}\right)$$

*holds for all A⊂T, then we say E is ${\left(m_{{⋄}_{\alpha }}^{\beta }\right)}^{*}$-measurable (or $\beta_{{⋄}_{\alpha }}$-measurable).*

In the following, the symbol $M\left({\left(m_{{⋄}_{\alpha }}^{\beta }\right)}^{*}\right)$ denotes the family of all ${\left(m_{{⋄}_{\alpha }}^{\beta }\right)}^{*}$-measurable subsets of T, then it forms a σ-algebra. We will use the symbols $\mu_{\Delta }^{\beta }$, $\mu_{\nabla }^{\beta }$ to denote the Lebesgue–Stieltjes Δ-measure and the Lebesgue–Stieltjes ∇-measure, respectively.

**Definition** **83** ([69])**.** *The function ${\left(m_{{⋄}_{\alpha }}^{\beta }\right)}^{*}:J_{T}\to \left[0,+\infty \right)$ restricted to $M\left({\left(m_{{⋄}_{\alpha }}^{\beta }\right)}^{*}\right)$ is called a Lebesgue–Stieltjes ${⋄}_{\alpha }$-measure and denoted by $\mu_{{⋄}_{\alpha }}^{\beta }$.*

We know that each interval on T can be covered by itself, which is the smallest cover, i.e., any interval is $\beta_{{⋄}_{\alpha }}$-measurable, thus for any interval *I*, pre-measure $m_{{⋄}_{\alpha }}^{\beta }\left(I\right)$ and $\beta_{{⋄}_{\alpha }}$-measure $\mu_{{⋄}_{\alpha }}^{\beta }\left(I\right)$ coincide, i.e.,

(i) $\mu_{{⋄}_{\alpha }}^{\beta }\left([a,b)\right)=\alpha \left(\beta \left(b^{-}\right)-\beta \left(a^{-}\right)\right)+\left(1-\alpha \right)\left(\beta \left(\rho {\left(b\right)}^{-}\right)-\beta \left(\rho {\left(a\right)}^{-}\right)\right)$,
(ii) $\mu_{{⋄}_{\alpha }}^{\beta }\left([a,b]\right)=\alpha \left(\beta \left(\sigma {\left(b\right)}^{+}\right)-\beta \left(a^{-}\right)\right)+\left(1-\alpha \right)\left(\beta \left(b^{+}\right)-\beta \left(\rho {\left(a\right)}^{-}\right)\right)$,
(iii) $\mu_{{⋄}_{\alpha }}^{\beta }\left((a,b]\right)=\alpha \left(\beta \left(\sigma {\left(b\right)}^{+}\right)-\beta \left(\sigma {\left(a\right)}^{+}\right)\right)+\left(1-\alpha \right)\left(\beta \left(b^{+}\right)-\beta \left(a^{+}\right)\right)$,
(iv) If b>σ(a), $\mu_{{⋄}_{\alpha }}^{\beta }\left((a,b\right))=\alpha \left(\beta \left(b^{-}\right)-\beta \left(\sigma {\left(a\right)}^{+}\right)\right)+\left(1-\alpha \right)\left(\beta \left(\rho {\left(b\right)}^{-}\right)-\beta \left(a^{+}\right)\right)$.

**Remark** **17.** *Note that the $\mu_{{⋄}_{\alpha }}^{\beta }$ measure value of a set E⊂T is the following combination*

$$\mu_{{⋄}_{\alpha }}^{\beta }\left(E\right)=\alpha \mu_{\Delta }^{\beta }\left(E\right)+\left(1-\alpha \right)\mu_{\nabla }^{\beta }\left(E\right),$$

*and we can obtain the $\mu_{\Delta }^{\beta }$ measure if α=1 and the $\mu_{\nabla }^{\beta }$ measure if α=0.*

**Theorem** **87** ([69])**.** *Let {c}⊂T. Then, it is $\mu_{{⋄}_{\alpha }}^{\beta }$-measurable and*

$$\mu_{{⋄}_{\alpha }}^{\beta }\left(\{c\}\right)=\mu_{{⋄}_{\alpha }}^{\beta }\left([c,c]\right)=\alpha \left(\beta \left(\sigma {\left(c\right)}^{+}\right)-\beta \left(c^{-}\right)\right)+\left(1-\alpha \right)\left(\beta \left(c^{+}\right)-\beta \left(\rho {\left(c\right)}^{-}\right)\right).$$

**Remark** **18.** *There is a fact that [c,c], (ρ(c),c] and [c,σ(c)) all have the same ${⋄}_{\alpha }$-measure, but their $\mu_{{⋄}_{\alpha }}^{\beta }$-measures are completely different. For $\mu_{{⋄}_{\alpha }}^{\beta }$-measure, we need to consider one-sided limits of a monotone increasing function β at the endpoints of a given interval.*

**Example** **5** ([69])**.** *Let T=[0,3]∪{7}∪[8,9], and*

$$\beta \left(t\right)=\left\{\begin{array}{cc} x+1\; & if\;\;0⩽t⩽3,\\ 5 & if\;\;3<t<8,\\ x^{2} & if\;\;8⩽t⩽9. \end{array}\right.$$

*Now, we calculate $\mu_{{⋄}_{\alpha }}$-measure and $\mu_{{⋄}_{\alpha }}^{\beta }$-measure of the following sets:*

$$\left(\rho (7),7\right],\left[7,7\right],\left[7,\sigma (7)\right).$$

*(1)*  Consider the $\mu_{{⋄}_{\alpha }}$-measure of the above sets:

*1*. $\mu_{{⋄}_{\alpha }}\left((\rho (7),7]\right)=\alpha \left(\sigma (7)-7\right)+\left(1-\alpha \right)\left(7-\rho \left(7\right)\right)=4-3\alpha .$
*2*. $\mu_{{⋄}_{\alpha }}\left([7,7]\right)=\alpha \left(\sigma (7)-7\right)+\left(1-\alpha \right)\left(7-\rho \left(7\right)\right)=4-3\alpha .$
*3*. $\mu_{{⋄}_{\alpha }}\left([7,\sigma (7))\right)=\alpha \left(\sigma (7)-7\right)+\left(1-\alpha \right)\left(7-\rho \left(7\right)\right)=4-3\alpha .$

*(2)*  Consider the $\mu_{{⋄}_{\alpha }}^{\beta }$-measure of the above sets:

*1*. $\mu_{{⋄}_{\alpha }}^{\beta }\left((\rho (7),7]\right)=\alpha \left(\beta \left(\sigma {\left(7\right)}^{+}\right)-\beta \left(7^{+}\right)\right)+\left(1-\alpha \right)\left(7^{+}-\beta \left(\rho {\left(7\right)}^{+}\right)\right)=59\alpha .$
*2*. $\mu_{{⋄}_{\alpha }}^{\beta }\left([7,7]\right)=\alpha \left(\beta \left(\sigma {\left(7\right)}^{+}\right)-\beta \left(7^{-}\right)\right)+\left(1-\alpha \right)\left(7^{+}-\beta \left(\rho {\left(7\right)}^{-}\right)\right)=58\alpha +1.$
*3*. $\mu_{{⋄}_{\alpha }}^{\beta }\left([7,\sigma (7))\right)=\alpha \left(\beta \left(\sigma {\left(7\right)}^{-}\right)-\beta \left(7^{-}\right)\right)+\left(1-\alpha \right)\left(7^{-}-\beta \left(\rho {\left(7\right)}^{-}\right)\right)=1-\alpha .$

**Example** **6** ([69])**.** *(1) Let T=R, then $\mu_{{⋄}_{\alpha }}^{\beta }$ and $\mu^{\beta }$ measures coincide since for all t∈T,σ(t)=ρ(t)=t.*

*(2)*  Let T=Z, then

*1*. *$\mu_{{⋄}_{\alpha }}^{\beta }\left([a,b)\right)=\alpha \left(\beta (b)-\beta (a)\right)+\left(1-\alpha \right)\left(\beta (b-1)-\beta (a-1)\right)$,*
*2*. *$\mu_{{⋄}_{\alpha }}^{\beta }\left([a,b]\right)=\alpha \left(\beta (b+1)-\beta (a)\right)+\left(1-\alpha \right)\left(\beta (b)-\beta (a-1)\right)$,*
*3*. *$\mu_{{⋄}_{\alpha }}^{\beta }\left((a,b]\right)=\alpha \left(\beta (b+1)-\beta (a+1)\right)+\left(1-\alpha \right)\left(\beta (b)-\beta (a)\right)$,*
*4*. *For b>a+1, $\mu_{{⋄}_{\alpha }}^{\beta }\left((a,b)\right)=\alpha \left(\beta (b)-\beta (a+1)\right)+\left(1-\alpha \right)\left(\beta (b-1)-\beta (a)\right)$.*

*(3)*  Let β:T→T and β(t)=t, then $\mu_{{⋄}_{\alpha }}^{\beta }$-measure turns into ${⋄}_{\alpha }$-measure as follows:

*1*. *$\mu_{{⋄}_{\alpha }}^{\beta }\left([a,b)\right)=\alpha \left(b-a\right)+\left(1-\alpha \right)\left(\rho (b)-\rho (a)\right)$,*
*2*. *$\mu_{{⋄}_{\alpha }}^{\beta }\left([a,b]\right)=\alpha \left(\sigma (b)-a\right)+\left(1-\alpha \right)\left(b-\rho (a)\right)$,*
*3*. *$\mu_{{⋄}_{\alpha }}^{\beta }\left((a,b]\right)=\alpha \left(\sigma (b)-\sigma (a)\right)+\left(1-\alpha \right)\left(b-a\right)$,*
*4*. *If b>σ(a), $\mu_{{⋄}_{\alpha }}^{\beta }\left((a,b)\right)=\alpha \left(b-\sigma (a)\right)+\left(1-\alpha \right)\left(\rho (b)-a\right)$,*

*which is equivalent to Theorem 80.*

**Example** **7** ([69])**.** *Let T=[0,4]∪{5}∪[7,10] and*

$$\beta \left(t\right)=\left\{\begin{array}{cc} 1+e^{-t}\; & if\;0⩽t⩽2,\\ 5 & if\;2<t<4,\\ 3t-4 & if\;4⩽t<8,\\ 2t^{2}+3 & if\;8⩽t⩽10. \end{array}\right.$$

*Now, we calculate $\mu_{\Delta }^{\beta }$-measure, $\mu_{\nabla }^{\beta }$-measure and $\mu_{{⋄}_{\alpha }}^{\beta }$-measure of the following sets:*

{3},{4},[4,7),(8,9],{5},[0,1).

*(1) Consider the $\mu_{\Delta }^{\beta }$-measures of the above sets:*

*1*. $\mu_{\Delta }^{\beta }\left(\{3\}\right)=\beta \left(\sigma {\left(3\right)}^{+}\right)-\beta \left(3^{-}\right)=\beta \left(3\right)-\beta \left(3\right)=0,$
*2*. $\mu_{\Delta }^{\beta }\left(\{4\}\right)=\beta \left(\sigma {\left(4\right)}^{+}\right)-\beta \left(4^{-}\right)=\beta \left(5^{+}\right)-\beta \left(4^{-}\right)=11-5=6,$
*3*. $\mu_{\Delta }^{\beta }\left([4,7)\right)=\beta \left(7^{-}\right)-\beta \left(4^{-}\right)=17-5=12,$
*4*. $\mu_{\Delta }^{\beta }\left((8,9]\right)=\beta \left(\sigma {\left(9\right)}^{+}\right)-\beta \left(\sigma {\left(8\right)}^{+}\right)=\beta \left(9\right)-\beta \left(8\right)=34,$
*5*. $\mu_{\Delta }^{\beta }\left(\{5\}\right)=\beta \left(\sigma {\left(5\right)}^{+}\right)-\beta \left(5^{-}\right)=\beta \left(7\right)-\beta \left(5\right)=6,$
*6*. *$\mu_{\Delta }^{\beta }\left([0,1)\right)=\beta \left(1^{-}\right)-\beta \left(0^{-}\right)=\infty$, since the limit from the left-hand side of β at t=0 is not defined.*

*(2) Consider the $\mu_{\nabla }^{\beta }$-measure of above sets:*

*1*. $\mu_{\nabla }^{\beta }\left(\{3\}\right)=\beta \left(3^{+}\right)-\beta \left(\rho {\left(3\right)}^{-}\right)=\beta \left(3\right)-\beta \left(3\right)=0,$
*2*. $\mu_{\nabla }^{\beta }\left(\{4\}\right)=\beta \left(4^{+}\right)-\beta \left(\rho {\left(4\right)}^{-}\right)=8-5=3,$
*3*. $\mu_{\nabla }^{\beta }\left([4,7)\right)=\beta \left(\rho {\left(7\right)}^{-}\right)-\beta \left(\rho {\left(4\right)}^{-}\right)=11-5=6,$
*4*. $\mu_{\nabla }^{\beta }\left((8,9]\right)=\beta \left(9^{+}\right)-\beta \left(8^{+}\right)=\beta \left(9\right)-\beta \left(8\right)=34,$
*5*. $\mu_{\nabla }^{\beta }\left(\{5\}\right)=\beta \left(5^{+}\right)-\beta \left(\rho {\left(5\right)}^{-}\right)=11-5=6,$
*6*. *$\mu_{\nabla }^{\beta }\left([0,1)\right)=\beta \left(\rho {\left(1\right)}^{-}\right)-\beta \left(\rho {\left(0\right)}^{-}\right)=\infty$ since the limit from the left-hand side of β at t=0 is not defined.*

*(3) Consider the $\mu_{{⋄}_{\alpha }}^{\beta }$-measure of the above sets:*

*1*. $\mu_{{⋄}_{\alpha }}^{\beta }\left(\{3\}\right)=\mu_{{⋄}_{\alpha }}^{\beta }\left([3,3]\right)=\alpha \left(\beta \left(\sigma {\left(3\right)}^{+}\right)-\beta \left(3^{-}\right)\right)+\left(1-\alpha \right)\left(\beta \left(3^{+}\right)-\beta \left(\rho {\left(3\right)}^{-}\right)\right)=\alpha \left(\beta \left(3^{+}\right)-\beta \left(3^{-}\right)\right)+\left(1-\alpha \right)\left(\beta \left(3^{+}\right)-\beta \left(3^{-}\right)\right)=0,$
*2*. $\mu_{{⋄}_{\alpha }}^{\beta }\left(\{4\}\right)=\mu_{{⋄}_{\alpha }}^{\beta }\left([4,4]\right)=\alpha \left(\beta \left(\sigma {\left(4\right)}^{+}\right)-\beta \left(4^{-}\right)\right)+\left(1-\alpha \right)\left(\beta \left(4^{+}\right)-\beta \left(\rho {\left(4\right)}^{-}\right)\right)=\alpha \left(\beta \left(5^{+}\right)-\beta \left(4^{-}\right)\right)+\left(1-\alpha \right)\left(\beta \left(4^{+}\right)-\beta \left(4^{-}\right)\right)=6\alpha +3\left(1-\alpha \right)=3+3\alpha ,$
*3*. *$\mu_{{⋄}_{\alpha }}^{\beta }\left([4,7)\right)=\alpha \left(\beta \left(7^{-}\right)-\beta \left(4^{-}\right)\right)+\left(1-\alpha \right)(\beta \left(\rho {\left(7\right)}^{-}\right)-\beta \left(\rho {\left(4\right)}^{-})\right)=\alpha \left(\beta \left(7^{-}\right)-\beta \left(4^{-}\right)\right)$$+\left(1-\alpha \right)\left(\beta \left(5^{-}\right)-\beta \left(4^{-}\right)\right)=12\alpha +6\left(1-\alpha \right)=6+6\alpha ,$*
*4*. *$\mu_{{⋄}_{\alpha }}^{\beta }\left((8,9]\right)=\alpha \left(\beta (\sigma {\left(9\right)}^{+}\right)-\beta \left(\sigma {\left(8\right)}^{+})\right)+\left(1-\alpha \right)\left(\beta \left(9^{+}\right)-\beta \left(8^{+}\right)\right)=\alpha \left(\beta \left(9^{+}\right)-\beta \left(8^{+}\right)\right)$$+\left(1-\alpha \right)\left(\beta \left(9^{+}\right)-\beta \left(8^{+}\right)\right)=34,$*
*5*. $\mu_{{⋄}_{\alpha }}^{\beta }\left(\{5\}\right)=\mu_{{⋄}_{\alpha }}^{\beta }\left(\left[5,5\right]\right)=\alpha \left(\beta \left(\sigma {\left(5\right)}^{+}\right)-\beta \left(5^{-}\right)\right)+\left(1-\alpha \right)\left(\beta \left(5^{+}\right)-\beta \left(\rho {\left(5\right)}^{-}\right)\right)=6,$
*6*. *$\mu_{{⋄}_{\alpha }}^{\beta }\left([0,1)\right)=\alpha \left(\beta \left(1^{-}\right)-\beta \left(0^{-}\right)\right)+\left(1-\alpha \right)\left(\beta \left(\rho {\left(1\right)}^{-}\right)-\beta \left(\rho {\left(0\right)}^{-}\right)\right)=\infty$ since the limit from left-hand side of β at t=0 and $\beta (\rho {\left(0\right)}^{-})$ are not defined.*

**Remark** **19.** *Through Example 7, one can obtain that the $\mu_{{⋄}_{\alpha }}^{\beta }$ measure value of a set E⊂T is the following combination*

$$\mu_{{⋄}_{\alpha }}^{\beta }\left(E\right)=\alpha \mu_{\Delta }^{\beta }\left(E\right)+\left(1-\alpha \right)\mu_{\nabla }^{\beta }\left(E\right),$$

*and we can obtain the $\mu_{\Delta }^{\beta }$ measure if α=1 and the $\mu_{\nabla }^{\beta }$ measure if α=0.*

## 7. Conclusions

In this review article, we present a survey of abstract analysis and applied dynamic equations on hybrid time scales. The content is divided into five sections including the almost periodic and almost automorphic theory, the uncertainty theory, the quaternion theory, coupled-jumpping theory, and combined measure theory on hybrid time scales. In each section, we demonstrate the very recent new results on both pure and applied mathematics, which is mainly in function analysis and applied dynamic equations. Moreover, the framework of knowledge and the idea of each section is clearly presented, and the potential future work is illustrated. The results presented in this article can be extended and generalized to study both pure mathematical analysis and real applications such as mathematical physics, biological dynamical models, and neural networks, etc.

## Figures and Tables

**Table 1:** The solution of (38).

***t***		$h_{11}\left(t\right)$	$h_{21}\left(t\right)$	$h_{31}\left(t\right)$	$h_{12}\left(t\right)$	$h_{22}\left(t\right)$	$h_{32}\left(t\right)$	$h_{13}\left(t\right)$	$h_{23}\left(t\right)$	$h_{33}\left(t\right)$	$h_{14}\left(t\right)$	$h_{24}\left(t\right)$	$h_{33}\left(t\right)$
9.7656 × 10^−4^		1	1	1	0	0	0	0	0	0	0	0	0
0.0020		1.0010	1.0000	1.0010	0.0008	0.0068	0.0039	0.0010	0.0029	0.0010	0.0010	0.0000	0.0029
0.0039		1.0020	1.0000	1.0020	0.0016	0.0137	0.0078	0.0020	0.0059	0.0020	0.0020	0.0000	0.0059
0.0078		1.0039	1.0001	1.0039	0.0033	0.0273	0.0156	0.0039	0.0117	0.0039	0.0039	0.0000	0.0117
0.0156		1.0078	1.0004	1.0079	0.0067	0.0547	0.0313	0.0079	0.0235	0.0079	0.0079	0.0001	0.0235
0.0313		1.0156	1.0015	1.0159	0.0135	0.1094	0.0625	0.0161	0.0471	0.0161	0.0161	0.0002	0.0471
0.0625		1.0313	1.0058	1.0322	0.0278	0.2188	0.1250	0.0332	0.0947	0.0332	0.0332	0.0010	0.0948
0.1250		1.0626	1.0233	1.0664	0.0585	0.4375	0.2500	0.0702	0.1914	0.0702	0.0703	0.0039	0.1916
0.2500		1.1260	1.0909	1.1406	0.1284	0.8750	0.5000	0.1550	0.3906	0.1550	0.1562	0.0156	0.3925
0.5000		1.2578	1.3302	1.3119	0.2991	1.7500	1.0000	0.3621	0.8119	0.3621	0.3737	0.0619	0.8275
1		1.5625	1.8754	1.7397	0.7385	3.5000	2.0000	0.8595	1.7397	0.8595	0.9755	0.2397	1.8634
2		2.3818	1.1525	2.8415	1.7508	7.0000	4.0000	1.4496	3.8415	1.4496	2.2232	0.8415	4.6829
4		−1.3459	1.4651	4.8186	2.1008	14.0000	8.0000	−2.3459	7.8186	−2.3459	3.3462	1.8186	6.3050
8		−2.7662	3.8058	1.9728	−6.8629	28.0000	16.0000	1.3429	8.9728	1.3429	−4.4870	−3.0272	7.8212
16		7.1962	3.1082	16.9149	11.2118	56.0000	32.0000	−3.4672	31.9149	−3.4672	7.8551	7.9149	39.2751
32		−30.3099	24.5432	12.3935	−19.9888	112.0000	64.0000	−6.4997	43.3935	−6.4997	3.6509	−4.6065	27.4062
step	***t***	$h_{1}\left(t\right)$				$h_{2}\left(t\right)$				$h_{3}\left(t\right)$			
0	9.7656 × 10^−4^	1				1				1			
1	0.0020	1.0010 + 0.0008*i* + 0.0010*j* + 0.0010*k*				1.0000 + 0.0068*i* + 0.0029*j* + 0.0000*k*				1.0010 + 0.0039*i* + 0.0010*j* + 0.0029*k*			
2	0.0039	1.0020 + 0.0016*i* + 0.0020*j* + 0.0020*k*				1.0000 + 0.0137*i* + 0.0059*j* + 0.0000*k*				1.0020 + 0.0078*i* + 0.0020*j* + 0.0059*k*			
3	0.0078	1.0039 + 0.0033*i* + 0.0039*j* + 0.0039*k*				1.0001 + 0.0273*i* + 0.0117*j* + 0.0000*k*				1.0039 + 0.0156*i* + 0.0039*j* + 0.0117*k*			
4	0.0156	1.0078 + 0.0067*i* + 0.0079*j* + 0.0079*k*				1.0004 + 0.0547*i* + 0.0235*j* + 0.0001*k*				1.0079 + 0.0313*i* + 0.0079*j* + 0.0235*k*			
5	0.0313	1.0156 + 0.0135*i* + 0.0161*j* + 0.0161*k*				1.0015 + 0.1094*i* + 0.0471*j* + 0.0002*k*				1.0159 + 0.0625*i* + 0.0161*j* + 0.0471*k*			
6	0.0625	1.0313 + 0.0278*i* + 0.0332*j* + 0.0332*k*				1.0058 + 0.2188*i* + 0.0947*j* + 0.0010*k*				1.0322 + 0.1250*i* + 0.0332*j* + 0.0948*k*			
7	0.1250	1.0626 + 0.0585*i* + 0.0702*j* + 0.0703*k*				1.0233 + 0.4375*i* + 0.1914*j* + 0.0039*k*				1.0664 + 0.2500*i* + 0.0702*j* + 0.1916*k*			
8	0.2500	1.1260 + 0.1284*i* + 0.1550*j* + 0.1562*k*				1.0909 + 0.8750*i* + 0.3906*j* + 0.0156*k*				1.1406 + 0.5000*i* + 0.1550*j* + 0.3925*k*			
9	0.5000	1.2578 + 0.2991*i* + 0.3621*j* + 0.3737*k*				1.3302 + 1.7500*i* + 0.8119 *j* + 0.0619*jk*				1.3119 + 1.0000*i* + 0.3621*j* + 0.8275*k*			
10	1	1.5625 + 0.7385*i* + 0.8595*j* + 0.9755*k*				1.8754 + 3.5000*i* + 1.7397*j* + 0.2397*k*				1.7397 + 2.0000*i* + 0.8595*j* + 1.8634*k*			
11	2	2.3818 + 1.7508*i* + 1.4496*j* + 2.2232*k*				1.1525 + 7.0000*i* + 3.8415*j* + 0.8415*k*				2.8415 + 4.0000*i* + 1.4496*j* + 4.6829*k*			
12	4	−1.3459 + 2.1008*i*− 2.3459*j* + 3.3462*k*				1.4651 + 14.0000*i* + 7.8186*j* + 1.8186*k*				4.8186 + 8.0000*i*− 2.3459*j* + 6.3050*k*			
13	8	−2.7662 − 6.8629*i* + 1.3429*j*− 4.4870*k*				3.8058 + 28.0000*i* + 8.9728*j*− 3.0272*k*				1.9728 + 16.0000*i* + 1.3429*j* + 7.8212*k*			
14	16	7.1962 + 11.2118*i*− 3.4672*j* + 7.8551*k*				3.1082 + 56.0000*i* + 31.9149*j* + 7.9149*k*				16.9149 + 32.0000*i*− 3.4672*j* + 39.2751*k*			
15	32	−30.3099 − 19.9888*i*− 6.4997*j* + 3.6509*k*				24.5432 + 112.0000*i* + 43.3935*j*− 4.6065*k*				12.3935 + 64.0000*i*− 6.4997*j* + 27.4062*k*			

**Table 2:** The solution of (41).

step	***t***	$h_{11}\left(t\right)$	$h_{21}\left(t\right)$	$h_{12}\left(t\right)$	$h_{22}\left(t\right)$	$h_{13}\left(t\right)$	$h_{23}\left(t\right)$	$h_{14}\left(t\right)$	$h_{24}\left(t\right)$
0	0	1	1	0	0	0	0	0	0
1	1	1.0000	1.0000	−14.9712	3.8000	0	0	−29.9425	4.0000
2	2	−22.1986	6.8883	−14.8956	3.7808	−1.2036	2.5922	−12.4595	1.6645
3	3	−24.9918	7.5973	11.8954	−3.0193	−0.4930	1.0618	3.6375	−0.4859
4	4	−1.4109	1.6119	16.9130	−4.2929	−0.0013	0.0028	0.1483	−0.0198
5	5	3.7555	0.3006	2.3799	−0.6041	0.2434	−0.5242	3.3894	−0.4528
6	6	1.5897	0.8503	−0.1744	0.0443	1.1430	−2.4619	−5.4514	0.7283
7	7	4.0143	0.2349	-10.3584	2.6292	0.5086	−1.0954	−28.1773	3.7642
8	8	−15.2980	5.1367	−18.7021	4.7470	−1.0700	2.3046	−19.7960	2.6445
9	9	−28.4662	8.4791	4.3334	−1.0999	−0.7850	1.6908	1.8614	−0.2487
10	10	−7.7127	3.2114	19.2623	−4.8892	−0.0340	0.0732	1.2122	−0.1619
11	11	4.7138	0.0574	5.7280	−1.4539	0.0813	−0.1751	2.0216	−0.2701
12	12	1.0001	1.0000	−0.0000	0.0000	0.9327	−2.0088	−0.0666	0.0089
13	13	4.7228	0.0551	−5.8547	1.4860	0.9187	−1.9787	−23.2944	3.1119
14	14	−7.9334	3.2675	−19.2938	4.8972	7 − 0.7442	1.6029	−25.9138	3.4618
15	15	−28.5219	8.4933	−4.0750	1.0343	−1.0456	2.2521	−2.3270	0.3109
step		***t***	$h_{1}\left(t\right)$				$h_{2}\left(t\right)$		
0		0	1				1		
1		1	1.0000 − 14.9712*i*− 29.9425*k*				1.0000 + 3.8000*i* + 4.0000*k*		
2		2	−22.1986 − 14.8956*i*− 1.2036*j*− 12.4595*k*				6.8883 + 3.7808*i* + 2.5922*j* + 1.6645*k*		
3		3	−24.9918 + 11.8954*i*− 0.4930*j* + 3.6375*k*				7.5973 − 3.0193*i* + 1.0618*j*− 0.4859*k*		
4		4	−1.4109 + 16.9130*i*− 0.0013*j* + 0.1483*k*				1.6119 − 4.2929*i* + 0.0028*j*− 0.0198*k*		
5		5	3.7555 + 2.3799*i* + 0.2434*j* + 3.3894*k*				0.3006 − 0.6041*i*− 0.5242*j*− 0.4528*k*		
6		6	1.5897 − 0.1744*i* + 1.1430*j*− 5.4514*k*				0.8503 + 0.0443*i*− 2.4619*j* + 0.7283*k*		
7		7	4.0143 −10.3584*i* + 0.5086*j*− 28.1773*k*				0.2349 + 2.6292*i*− 1.0954*j* + 3.7642*k*		
8		8	−15.2980 − 18.7021*i*− 1.0700*j*− 19.7960*k*				5.1367 + 4.7470*i* + 2.3046*j* + 2.6445*k*		
9		9	−28.4662 + 4.3334*i*− 0.7850*j* + 1.8614*k*				8.4791 − 1.0999*i* + 1.6908*j*− 0.2487*k*		
10		10	−7.7127 + 19.2623*i*− 0.0340*j* + 1.2122*k*				3.2114 − 4.8892*i* + 0.0732*j*− 0.1619*k*		
11		11	4.7138 + 5.7280*i* + 0.0813*j* + 2.0216*k*				0.0574 − 1.4539*i*− 0.1751*j*− 0.2701*k*		
12		12	1.0001 − 0.0000*i* + 0.9327*j*− 0.0666*k*				1.0000 + 0.0000*i*− 2.0088*j* + 0.0089*k*		
13		13	4.7228 − 5.8547*i* + 0.9187*j*− 23.2944*k*				0.0551 + 1.4860*i*− 1.9787*j* + 3.1119*k*		
14		14	−7.9334 − 19.2938*i*− 0.7442*j*− 25.9138*k*				3.2675 + 4.8972*i* + 1.6029*j* + 3.4618*k*		
15		15	−28.5219 − 4.0750*i*− 1.0456*j*− 2.3270*k*				8.4933 + 1.0343*i* + 2.2521*j* + 0.3109*k*		

## Data Availability

Data sharing not applicable.
